# Supplementary material for: SCRQE: Subjective comparative relation quintuple extraction from questions in product domain
Source: PLoS One. 2025 May 27;20(5):e0319824. doi: 10.1371/journal.pone.0319824 (PMC12112347; doi:10.1371/journal.pone.0319824)
Supplement: S1 Appendix A — (DOCX) [file pone.0319824.s001.docx]

# **Appendix A: Auxiliary Sentence Generation Algorithm**

In this section, we detail Algorithm 1, which is central to preparing data for the Entity Role Identification (ERI) task. This algorithm is designed to systematically construct auxiliary sentences that are pivotal for the accurate classification of entities within comparative questions, thereby facilitating a detailed and structured analysis of comparative relations. For example, by analyzing the entities and aspects of the question below, we intend to generate all possible pseudo-sentences alongside their related labels:

**Question:** "Is the Samsung Galaxy M31 superior to the Samsung Galaxy A50 in terms of software performance?"

- **Entities:** Samsung Galaxy M31, Samsung Galaxy A50
- **Aspect:** Software performance
- **Auxiliary Sentences:**
  - "Samsung Galaxy M31 - software performance" (**Label:** Subject)
  - "Samsung Galaxy A50 - software performance" (**Label:** Object)

This example illustrates the process of utilizing identified entities and aspects from a given question to generate auxiliary sentences. These sentences explicitly articulate the comparative relationship implied within the question, thus serving as foundational inputs for the sentence-pair classification task inherent to the ERI process.

Algorithm 1: Data Preparation for ERI Task via Augmented Auxiliary Sentence Construction

Inputs: Dict: Dictionary of questions to entity, aspect, and comparative relation lists.

Outputs:

- question_NLI_Pair_List: List of tuples, each a question paired with an NLI statement.

- label_List: List of corresponding labels for the NLI statements.

Method:

1: Init question_NLI_Pair_List, label_List as empty.

2: For each (question, [entities, aspects, compRelations]) in Dict do

3: If aspects empty then

4: question += ", in terms of features?" // Enhance question

5: aspects = ["features"] // Add generic aspect

6: End if

7: For each aspect in aspects do

8: If entities empty then

9: Append (question, aspect), "None" to question_NLI_Pair_List, label_List.

10: Else

11: For each entity in entities do

12: NLI = entity + "–" + aspect // Construct NLI

13: label = "None" // Initialize label as "None"

14: For each compRel in compRelations do

15: If aspect is in compRel and entity is the "Subject Entity" then

16: label = "Subject" // Assign "Subject" label

17: ElseIf aspect is in compRel and entity is the "Object Entity" then

18: label = "Object" // Assign "Object" label

19: End if

20: End for

21: Append (question, NLI), label to question_NLI_Pair_List, label_List.

22: End for

23: End if

24: End for

25: End for

The core objective of this algorithm is to generate auxiliary sentences from the dataset's questions. These sentences are instrumental in identifying the comparative roles of entities, enabling a nuanced understanding of the dataset's intricate comparative structures.

- **Input:** A dataset of questions, each with identified entities and aspects.
- **Output:** A structured set of auxiliary sentences for each question, indicating the comparative roles implied within.

To achieve this, the algorithm follows a structured procedure:

1. **Identify and extract entities (E) and aspects (A)** from each question in the dataset. This step involves parsing the question to delineate the components critical for comparative analysis.
2. **Generate auxiliary sentences** utilizing the identified entities and aspects. This process ensures that each generated sentence encapsulates the comparative analysis implied by the original question, making explicit the relationships that may only be implicitly stated.
3. **Assign a label (Subject, Object, or None)** to each auxiliary sentence, reflecting the comparative role each entity assumes within the context of the question. This labeling is crucial for the subsequent sentence-pair classification task, allowing for a nuanced understanding of the dataset's comparative structures.

These steps ensure a methodical approach to transforming raw questions into structured formats conducive to comparative analysis, facilitating the accurate identification and categorization of comparative roles.

For a more understanding of the workings of Algorithm 1, Table 1-A showcases its application on two sample questions.

**Table 1-A.** Demonstrative Execution of Algorithm 1 across Varied Comparative Question Types

| Example 1 | **Key** | | “*Does the Oppo F19 Pro have the same sound quality as the Realme X7?*” |
| --- | --- | --- | --- |
|  | **Values** | **entities** | [Oppo F19, Realme X7] |
|  |  | **aspects** | [sound quality] |
|  |  | **compRelations** | [[[Oppo F19, Realme X7], sound quality, [], E]] |
|  | **question_NLI_Pair_List** | | [ [*'Does the Oppo F19 Pro have the same sound quality as the Realme X7?*', 'Oppo F19 Pro - sound quality'],  [*'Does the Oppo F19 Pro have the same sound quality as the Realme X7?*', 'Realme X7 - sound quality'] ] |
|  | **label_List** | | [Subject, Subject] |
| Example 2 | **Key** | | “*Does OnePlus 6 has been able to beat its ZenFone 5Z?*” --->  *'Does OnePlus 6 has been able to beat its ZenFone 5Z , in terms of features?* |
|  | **Values** | **entities** | [OnePlus 6, ZenFone 5Z] |
|  |  | **aspects** | [features] |
|  |  | **compRelations** | [[[OnePlus 6], features, [ZenFone 5Z], SB]] |
|  | **question_NLI_Pair_List** | | [ [*'Does OnePlus 6 has been able to beat its ZenFone 5Z , in terms of features?*', 'OnePlus 6 - features'],  [*'Does OnePlus 6 has been able to beat its ZenFone 5Z , in terms of features?*', 'ZenFone 5Z - features'] ] |
|  | **label_List** | | [Subject, Object] |
