# Supplementary material for: SCRQE: Subjective comparative relation quintuple extraction from questions in product domain
Source: PLoS One. 2025 May 27;20(5):e0319824. doi: 10.1371/journal.pone.0319824 (PMC12112347; doi:10.1371/journal.pone.0319824)
Supplement: S2 Appendix B — (DOCX) [file pone.0319824.s002.docx]

# **Appendix B: Analysis of XOR and X-Type Preferences in Comparative Questions**

In this section of the appendix, we delve into the expression of preferences within subjective comparative questions, which can vary considerably based on the context and the specifics of the comparison. This analysis is instrumental in understanding the nuanced ways preferences are articulated by users.

**Table 1-B.** Exploring XOR-type preferences within subjective comparative questions

| **Subjective Comparative Question** | **Identified Preference** |
| --- | --- |
| Which phone is better overall performance, Xiaomi Mi Max or Lenovo Phab 2 plus? | XOR-Better |
| Should I buy one out of Samsung Galaxy Note 4, Samsung Galaxy K Zoom, and Samsung NX Mini? I want the best camera with the best results in all conditions. | XOR-Strong Better |
| Which smartphone has the more powerful processor between the Samsung Galaxy A70S and the Realme XT? | XOR-Strong Better |
| Samsung Galaxy A80 or iPhone 10, which one has lower quality? | XOR-Worse |
| Samsung Galaxy A80 or iPhone 10, which one has awful quality? | XOR-Strong Worse |
| What phone should I never buy, Samsung Galaxy s18 iPhone 12? | XOR-Strong Worse |

**Table 2- B.** Exploring X-type preferences within subjective comparative questions

| **Subjective Comparative Question** | **Identified Preference** |
| --- | --- |
| How is the quality of the iPhone 11 compared to the Samsung Galaxy Note 10 Plus? | X |
| What is your review of the Samsung Galaxy S10 and S10+ comparison? |  |
| Is it better or not to upgrade my LG V20 to Samsung Galaxy S7 Edge? |  |
| What is the satisfaction level of the Xiaomi Mi3 compared to Samsung and Apple? | X-Strong Better |
| How much better was the battery of the iPhone 13 mini compared to the iPhone 12 mini? |  |
| How powerful is the camera shooting function of iPhone XS Max rather than iPhone 13 Pro Max? |  |
| How much worse is the camera resolution in the Samsung Galaxy S21 FE compared to OnePlus 10 Pro? | X-Strong Worse |
| How weak is iPhone X graphics compared to Oppo Find X3 Pro? |  |
| How bad is the iPhone 13 mini battery than iPhone 13? |  |

These tables are formatted to provide a direct reference to the XOR and X-type preferences identified within the subjective
