# Supplementary material for: SCRQE: Subjective comparative relation quintuple extraction from questions in product domain
Source: PLoS One. 2025 May 27;20(5):e0319824. doi: 10.1371/journal.pone.0319824 (PMC12112347; doi:10.1371/journal.pone.0319824)
Supplement: Attachment — Submitted filename: Detailed Response to Reviewers.docx [file pone.0319824.s004.docx]

**View Letter**

| **Date:** | Feb 19 2024 05:18PM |
| --- | --- |
| **To:** | "Afsaneh Fatemi" a_fatemi@eng.ui.ac.ir |
| **From:** | "PLOS ONE" plosone@plos.org |
| **Subject:** | PLOS ONE Decision: Revision required [PONE-D-23-39359] |

PONE-D-23-39359

SCRQE: Subjective Comparative Relation Quintuple Extraction from Questions in Product Domain

PLOS ONE

Dear Dr. Fatemi,

Thank you for submitting your manuscript to PLOS ONE. After careful consideration, we feel that it has merit but does not fully meet PLOS ONE&rsquo;s publication criteria as it currently stands. Therefore, we invite you to submit a revised version of the manuscript that addresses the points raised during the review process.

**Please note that we have only been able to secure a single reviewer to assess your manuscript. We are issuing a decision on your manuscript at this point to prevent further delays in the evaluation of your manuscript. Please be aware that the editor who handles your revised manuscript might find it necessary to invite additional reviewers to assess this work once the revised manuscript is submitted. However, we will aim to proceed on the basis of this single review if possible.**

**The reviewer has raised a range of concerns, including methodological clarity, the context and overall justification for this work compared to other work in the field, and appropriate comparisons with state-of-the-art reports on similar methods. Please ensure you address each of the reviewers&#39; comments when revising your manuscript.**

Please submit your revised manuscript by Apr 15 2024 11:59PM. If you will need more time than this to complete your revisions, please reply to this message or contact the journal office at [plosone@plos.org](mailto:plosone@plos.org). When you&#39;re ready to submit your revision, log on to https://www.editorialmanager.com/pone/ and select the &#39;Submissions Needing Revision&#39; folder to locate your manuscript file.

Please include the following items when submitting your revised manuscript:

- A rebuttal letter that responds to each point raised by the academic editor and reviewer(s). You should upload this letter as a separate file labeled &#39;Response to Reviewers&#39;.
- A marked-up copy of your manuscript that highlights changes made to the original version. You should upload this as a separate file labeled &#39;Revised Manuscript with Track Changes&#39;.
- An unmarked version of your revised paper without tracked changes. You should upload this as a separate file labeled &#39;Manuscript&#39;.

If you would like to make changes to your financial disclosure, please include your updated statement in your cover letter. Guidelines for resubmitting your figure files are available below the reviewer comments at the end of this letter.

If applicable, we recommend that you deposit your laboratory protocols in protocols.io to enhance the reproducibility of your results. Protocols.io assigns your protocol its own identifier (DOI) so that it can be cited independently in the future. For instructions see: <https://journals.plos.org/plosone/s/submission-guidelines#loc-laboratory-protocols>. Additionally, PLOS ONE offers an option for publishing peer-reviewed Lab Protocol articles, which describe protocols hosted on protocols.io. Read more information on sharing protocols at [https://plos.org/protocols?utm_medium=editorial-email&amp;utm_source=authorletters&amp;utm_campaign=protocols](https://plos.org/protocols?utm_medium=editorial-email&utm_source=authorletters&utm_campaign=protocols).

We look forward to receiving your revised manuscript.

Kind regards,

Hugh Cowley
Staff Editor
PLOS ONE

**Response to Editor's Invitation for Manuscript Revision**

Dear Editor,

We extend our deepest appreciation for the thorough evaluation of our manuscript and the opportunity to refine and resubmit it for consideration in PLOS ONE. The insightful comments and suggestions from the review process are invaluable, and we are confident that addressing them will substantially elevate the impact and clarity of our research.

Upon a meticulous examination of the feedback provided, we have implemented comprehensive revisions to our manuscript. These modifications particularly focus on enhancing methodological clarity, providing a more robust context and justification for our work, and making more pertinent comparisons with the latest developments in our field, as advised.

In alignment with your submission instructions, we are prepared to submit the revised manuscript by the stipulated deadline, along with the necessary documents:

1. **Rebuttal Letter:** A meticulously crafted document addressing each comment made by the academic editor and reviewers. This will be uploaded as a separate file under the label 'Response to Reviewers'.
2. **Marked-Up Manuscript:** This version will display all changes made to the original manuscript, clearly marked to facilitate review. It will be submitted as 'Revised Manuscript with Track Changes'.
3. **Unmarked Manuscript:** A clean version of the revised manuscript will also be provided, without the tracked changes, labeled 'Manuscript'.

We will also re-examine our financial disclosure statement and update it as necessary in our cover letter.

Regarding the deposit of our laboratory protocols on protocols.io, we recognize the importance of this practice in enhancing research reproducibility and transparency. Thus, we have decided to deposit our protocols as recommended. We are committed to contributing to the scientific community's efforts to enhance research integrity and accessibility.

We are eager to advance our contribution to the PLOS ONE community and are grateful for the guidance and support provided by your esteemed journal throughout this process. Should you need any further information or clarification, please feel free to reach out to us.

Warm regards,

Dr. Afsaneh Fatemi

Journal Requirements:

1. When submitting your revision, we need you to address these additional requirements.

Please ensure that your manuscript meets PLOS ONE&#39;s style requirements, including those for file naming. The PLOS ONE style templates can be found at 
https://journals.plos.org/plosone/s/file?id=wjVg/PLOSOne_formatting_sample_main_body.pdf and 
<https://journals.plos.org/plosone/s/file?id=ba62/PLOSOne_formatting_sample_title_authors_affiliations.pdf>

**Response to Editorial Comment 1: Adherence to PLOS ONE Style Guidelines:**

Dear Editor,

We are grateful for your guidance regarding the style requirements of PLOS ONE. Your direction has been instrumental in ensuring the conformity of our manuscript to the journal's formatting standards.

In response to your feedback, we have meticulously examined the PLOS ONE style templates and implemented the necessary adjustments to our manuscript to ensure complete compliance. These adjustments encompass:

1. **Manuscript Formatting**: We have restructured the manuscript layout according to the guidelines outlined in the PLOS ONE formatting sample for the main body. This involved modifying the text arrangement, font dimensions, heading styles, and other formatting aspects as prescribed by the template.
2. **Title, Authors, and Affiliations:** Furthermore, we have reorganized the title, authors, and affiliations section of our manuscript in accordance with the PLOS ONE formatting sample for title, authors, and affiliations. We have ensured the clear and accurate presentation of all pertinent information in the appropriate sequence.
3. **File Naming Convention**: Additionally, we have adjusted the file naming of our manuscript and accompanying documents to adhere to PLOS ONE's specifications. We have ensured that all submitted files are appropriately named to facilitate easy identification and organization.

We are confident that these revisions have brought our manuscript into full alignment with PLOS ONE's style guidelines. We remain committed to upholding the highest standards of presentation and are appreciative of your invaluable assistance throughout this process.

Please find the revised manuscript attached for your review. We eagerly anticipate any further feedback or instructions you may have.

2. You indicated that ethical approval was not necessary for your study. We understand that the framework for ethical oversight requirements for studies of this type may differ depending on the setting and we would appreciate some further clarification regarding your research. Could you please provide further details on why your study is exempt from the need for approval and confirmation from your institutional review board or research ethics committee (e.g., in the form of a letter or email correspondence) that ethics review was not necessary for this study? Please include a copy of the correspondence as an &quot;Other&quot; file.

**Response to Editorial Comment 2: Addressing Ethical Considerations**

Dear Editor,

Thank you for your attention to the ethical aspects of our study, as outlined in your recent communication. We understand the importance of this issue and are committed to ensuring the highest standards of ethical conduct in our research.

Our study, titled "SCRQE: Subjective Comparative Relation Quintuple Extraction from Questions in Product Domain" involves the development of the SCRQD dataset, which comprises 1,275 subjective comparative questions extracted from publicly available sources, specifically focusing on the smartphone domain. These questions were sourced from online platforms such as Amazon and Quora, where users freely post their opinions and inquiries regarding various products, including smartphones.

Given the nature of our work, which solely utilizes publicly available data that does not involve human subjects or personal identifiable information, we initially assessed that formal ethical approval was not required. The dataset compilation involved the following ethical considerations:

- **Data Collection:** The questions were collected from public domains, ensuring no infringement on privacy or intellectual property rights. Our methodology explicitly avoids the use of personally identifiable information, aligning with ethical standards for public data use.
- **Annotation Process:** Our annotators, skilled in computational linguistics, were engaged in analyzing and annotating these publicly available questions. The process was conducted with a clear understanding of the ethical guidelines, ensuring the integrity of the data analysis without involving human subjects in a manner that requires ethical oversight.
- **Compliance and Ethical Measures:** Throughout the dataset's development and analysis, strict adherence to the terms and conditions of the source platforms was maintained. This included the ethical consideration of using publicly shared information for academic research purposes without breaching privacy or copyright policies.

We do not have human or animal subjects, nor do we utilize proprietary organizational information in our research. Based on the guidelines set forth by the ethics committee of our institution, our study does not require ethical approval due to its use of publicly available data and lack of direct interaction with individuals. However, should the journal deem it necessary, we are prepared to seek formal ethical approval and will provide documentation upon request.

We hope this response adequately addresses your request for further clarification on the ethical considerations of our study. We remain dedicated to upholding the highest ethical standards in our research and are available to provide any additional information or clarification as needed.We hope this response adequately addresses your request for further clarification on the ethical considerations of our study. We remain dedicated to upholding the highest ethical standards in our research and are available to provide any additional information or clarification as needed.

Thank you for your consideration and guidance on this matter. We look forward to your feedback.

3. Please provide additional details regarding participant consent. In the ethics statement in the Methods and online submission information, please ensure that you have specified (a) whether consent was informed and (b) what type you obtained (for instance, written or verbal, and if verbal, how it was documented and witnessed). If your study included minors, state whether you obtained consent from parents or guardians. If the need for consent was waived by the ethics committee, please include this information.
If you are reporting a retrospective study of medical records or archived samples, please ensure that you have discussed whether all data were fully anonymized before you accessed them and/or whether the IRB or ethics committee waived the requirement for informed consent. If patients provided informed written consent to have data from their medical records used in research, please include this information.

**Response to Editorial Comment 3: Clarification on Ethical Exemption**

Dear Editor,

Thank you for highlighting the ethical considerations of our study titled "SCRQE: Subjective Comparative Relation Quintuple Extraction from Questions in Product Domain." Our research compiles the SCRQD dataset from publicly available questions on platforms like Amazon and Quora, focusing on the smartphone domain without involving human or animal subjects, personal identifiable information, or medical records. Given the nature of our data collection and the guidelines from our institution's ethics committee, our project was deemed exempt from formal ethical approval. However, we are fully prepared to seek and provide such approval if deemed necessary by the journal. We trust this clarifies our position and demonstrates our commitment to upholding the highest standards of ethical research.

Thank you for your attention and advice regarding this issue. We anticipate hearing your thoughts on it.

4. In your Methods section, please include additional information about your dataset and ensure that you have included a statement specifying whether the collection and analysis method complied with the terms and conditions for the source of the data.

**Response to Editor's Comment on Dataset Enhancement and Compliance**

Dear Editor,

Thank you for your insightful feedback and the chance to refine our manuscript. We have paid particular attention to your request for more details on our dataset, as well as ensuring our methods align with the data source's terms and conditions.

As a result, **we've introduced a new segment in our “Methodology” section, named " 3.2.2. Data Quality and Integrity,"** dedicated to addressing these points. Here's a summary of what this addition covers, responding directly to the reviewer’s feedback:

- **Foundation and Gathering of the Dataset:** This part elucidates the origin of our dataset, drawing from user contributions on platforms like Quora and Amazon within the smartphone domain, and our adherence to specific selection criteria.
- **Enhancement and Generation of the SCQRD Dataset:** Facing a lack of questions that fully met our criteria (subjective comparative questions that clearly mention the entities being compared), we modified existing contributions and formulated new ones, enhancing the dataset's variety. This includes sentence transformations, content adjustments, and the invention of questions that, while fictional, remain realistic and expand our dataset’s coverage.
- **Adherence to Ethical and Compliance Standards:** We underscore our commitment to the data sources' terms, prioritizing ethical data handling, privacy, and intellectual property. We elaborate on our compliance strategy and the steps taken to omit any personally identifiable information.

The inclusion of "3.2.2. Data Quality and Integrity " in our methodology section aims to transparently convey the steps taken in dataset development, our ethical stance, and adherence to legal requirements. We trust this update fully addresses the concerns raised and adds to our manuscript's clarity and integrity.

We are thankful for this opportunity to elucidate these critical aspects of our research and appreciate the valuable guidance from the reviewers. We are open to and eager for any additional feedback.

**To ensure we fully honor the editor’s suggestions and our commitment to detailed responses, we have also included this new section in our letter of reply.**

**Revised Section 3.2.2. Data Quality and Integrity**

Maintaining high standards for data quality and integrity is crucial for the creation and effectiveness of the dataset. This section discusses the methods used to develop a dataset that supports the needs of NLP research effectively. By employing thorough dataset preparation techniques, incorporating distinctive attributes, implementing a meticulously planned annotation procedure, and applying rigorous criteria for assessing inter-annotator consistency, we adopt a strategy that emphasizes accuracy, dependability, and ethical practices in constructing the dataset.

**Dataset Creation: Detailed Preparation Methods and Specifications**

This section offers a thorough explanation of the careful steps and criteria involved in creating the SCQRD dataset, which is fundamental to its construction. It delves into the methodology used, the selection and modification process of data, and the strategic planning behind compiling the dataset to guarantee its integrity and usefulness for intended research purposes.

- **Origin and Assembly of Data:** We started with data inspired by user contributions on platforms like Amazon (www.amazon.com) and Quora (www.quora.com), focusing on the smartphone domain. Finding questions that met our specific needs (subjective comparative questions) was difficult, as we required questions to clearly mention the entities being compared, be precisely phrased, and fit within our set categories.
- **Development and Enhancement of the SCQRD Dataset:** Due to the scarcity of questions that exactly matched our research criteria, we modified existing questions and formulated new ones to diversify our dataset. This process involved:
  - **Sentence Modification:** Changing existing sentences into questions that fit our research.
  - **Adjusting Content:** Adding, deleting, or modifying parts of questions to increase their specificity and applicability.
  - **Inventing Data:** Adding fictional yet realistic questions to expand the dataset’s coverage of potential scenarios and queries.
- **Verification and Endorsement Process:** The revised and newly formulated questions underwent a strict validation process, managed by experts with knowledge in the smartphone field's writing and conceptual aspects. Their endorsement confirmed the dataset's relevance and alignment with our research aims.
- **Strategies for Reducing Bias in Dataset Assembly:** To minimize bias in question selection and presentation, we adopted several strategies tailored to question classification tasks. First, we gathered questions from a variety of online sources, not just mainstream platforms, to ensure a diverse range of question styles and subjects, thereby reducing selection bias. Next, in modifying and generating questions, we made sure to cover various smartphone categories to reflect a wide array of user inquiries and needs, aiming to lessen content bias. To combat annotator bias, detailed guidelines and regular calibration sessions were employed to ensure consistent and neutral question classification.
- **Ethical and Compliance Measures:** All processes of data collection, modification, and creation were performed in strict adherence to the terms and conditions of the original platforms. Ethical considerations were a top priority, including the avoidance of any personally identifiable information to protect the privacy and confidentiality of individuals involved in the initial data gathering.

**Annotation Process and Inter-Annotator Agreement Analysis**

This study leveraged the expertise of three annotators with backgrounds in computational linguistics and specializations in product domain sentiment analysis. Prior to annotation, the team underwent a comprehensive training session focused on the intricacies of subjective comparative questions. The training included an extensive review of key concepts and definitions relevant to comparative relations, such as entities, aspects, constraints, and comparative preferences. This ensured a shared understanding and application of the annotation guidelines. The complexity of identifying subject entities, object entities, and compared aspects within multiple comparative relations was emphasized due to their critical role in the task.

The annotation process was designed to be iterative, allowing annotators to independently label a shared set of questions before convening to discuss discrepancies and refine their interpretations of the guidelines. This collaborative approach facilitated a dynamic refinement of annotation practices, enhancing consistency across the dataset.

To quantify the level of agreement among the annotators, we employed a multifaceted approach, utilizing several inter-annotator agreement (IAA) metrics tailored to the specific aspects of the annotation task:

- **Fleiss' Kappa [24]:** This metric was chosen to assess the agreement on the identification of entities and aspects, which are foundational to constructing the comparative relations. Given its suitability for categorical data and its accommodation of multiple raters, Fleiss' Kappa provided a robust measure of consensus for these critical components.
- **Weighted Kappa [25]:** To account for the ordinal nature of comparative preferences, encompassing categories like "Better" to "XOR-Strong Worse," Weighted Kappa was employed. This metric assigns different weights to disagreements based on the severity within preference categories, offering a more nuanced assessment of annotator consistency in this intricate judgment domain.
- **Alpha U [26]:** To evaluate the agreement on extracting entire comparative relations, including Subject Entity, Object Entity, Compared Aspect, Constraint, and Comparative Preference, Alpha U was chosen. This metric is an adaptation of Krippendorff's Alpha [27] specifically designed for tasks involving segmenting and classifying text. By employing Alpha U, the study accurately measured annotator agreement on both the identification and contextual classification of these relations.

High inter-annotator agreement is essential for validating the reliability of the annotated dataset, especially in the complex domain of subjective comparative questions. This section summarizes the agreement metrics, reflecting the effectiveness of the annotation process across various components.

- **Entity and Aspect Identification:** The annotation process yielded a Fleiss' Kappa score of 0.94 for entity identification and 0.89 for aspect identification. These scores, indicating almost perfect agreement, attest to the effectiveness of our annotation training and guidelines in achieving a high degree of consistency among annotators for these fundamental components.
- **Constraint Recognition:** The Fleiss' Kappa score for constraint identification was 0.78, signifying substantial agreement. The slightly lower score compared to entity and aspect identification reflects the inherent challenges in recognizing and categorizing constraints, which often require a deeper inference of the question context.
- **Subject Entity, Object Entity, and Compared Aspect Identification:** The IAA metrics underscore the consistency in annotator judgments across key components, with Fleiss' Kappa scores revealing: Subject Entities at 95%, indicating almost perfect agreement; Object Entities at 92%, demonstrating substantial consensus; and Compared Aspects at 89%, reflecting significant agreement. These scores collectively highlight the effectiveness of our annotator training and guidelines in ensuring high-quality, consistent annotations for the foundational elements of comparative relation extraction.
- **Comparative Preference Classification:** The application of Weighted Kappa resulted in a score of 0.76 for comparative preferences. This substantial agreement level highlights the annotators' ability to navigate the intricate spectrum of preferences, supported by the detailed categorization scheme developed for this task.
- **Comparative Relation Extraction:** The comprehensive approach to annotating complete comparative relations was reflected in an Alpha U score of 0.80. This high level of agreement among annotators underscores the effectiveness of our iterative annotation process and collaborative discussions in clarifying complex relations, including the accurate identification of Subject Entities, Object Entities, and Compared Aspects.

The inter-annotator agreement analysis revealed a high level of consensus across all components of the annotation task, validating the reliability of the annotated dataset for extracting subjective comparative relations. While the process identified areas for improvement, particularly in constraint recognition, the overall results affirm the robustness of the annotation methodology. The collaborative and iterative nature of the annotation process, along with the targeted use of multiple IAA metrics, has established a strong foundation for subsequent analyses and model development based on this dataset.

**Addressing Disagreements among Annotators**

Despite the overall high levels of agreement, instances of disagreement among annotators served as valuable opportunities for further refining our annotation process and guidelines. Disagreements primarily arose in two areas: constraint recognition and the classification of nuanced comparative preferences.

To address these disagreements, we implemented a multifaceted resolution strategy, which included:

- **Guideline Refinement:** Disagreement analysis identified areas for improved clarity within the annotation guidelines. For constraint recognition, additional examples of explicit constraints were incorporated to ensure consistent identification. Similarly, comparative preferences received more detailed definitions and examples, particularly for complex categories like "X-types," "XOR-types," and "Non-Gradable." These refinements aim to achieve a shared understanding among annotators.
- **Focused Annotator Training:** Following guideline refinement, additional training sessions were held, focusing specifically on areas where disagreements were most prevalent. These sessions included interactive discussions on challenging examples, allowing annotators to explore and reconcile different interpretations of the guidelines.
- **Consensus Meetings:** For questions that continued to pose challenges after the initial annotation and review, consensus meetings were convened. During these meetings, annotators discussed their rationales for their classifications, with a senior member of the research team facilitating a consensus decision. This collaborative approach not only resolved individual instances of disagreement but also contributed to the continuous improvement of the annotation process.

These resolution strategies led to a marked decrease in the rate of disagreement in subsequent annotation rounds. Specifically:

- **Constraint Recognition:** The refinement of guidelines and focused training resulted in an increase in the Fleiss' Kappa score for constraint recognition from 0.78 to 0.82, indicating enhanced agreement and a more consistent application of the guidelines.
- **Comparative Preference Classification:** The consensus meetings and enriched annotation examples for comparative preferences improved the Weighted Kappa score from 0.76 to 0.81, reflecting a higher level of annotator consistency in this complex judgment area.
- **Holistic Enhancement across Annotations:** The improvements in Comparative Preference Classification (CPC) and Constraint Extraction, coupled with the meticulous clarification of Subject Entities, Object Entities, and Compared Aspects, collectively led to an overarching enhancement in SCRQD dataset's annotation quality. The observed improvement in the Alpha U score from 0.80 to 0.84 highlights the significant impact of the implemented resolution strategies. These strategies have facilitated the more accurate, reliable, and comprehensive extraction of comparative relations.

The iterative process of addressing annotator disagreements through guideline refinement, focused training, and consensus discussions proved instrumental in enhancing the reliability of our annotated dataset. Not only did this approach resolve specific instances of disagreement, but it also fostered a deeper collective understanding among the annotators, contributing to the overall quality and coherence of the dataset. These efforts underscore the dynamic nature of the annotation process and highlight the importance of continuous engagement and adaptation in achieving high-quality annotations for complex NLP tasks.

**Methodology for Assessing Annotator Agreement on Comparative Preferences**

To quantify the inter-annotator agreement on the Comparative Preference Classification (CPC) within the SCRQD dataset, we employed the Weighted Kappa statistic. This statistical method is particularly effective due to its ability to account for the ordinal nature of the data and the varying degrees of disagreement between different types of comparative preferences.

**Table 12.** Construction of the Weighted Disagreement Matrix

|  | **SW** | **W** | **E** | **B** | **SB** | **XOR-SW** | **XOR-W** | **XOR-E** | **XOR-B** | **XOR-SB** | **X-SW** | **X** | **X-SB** | **NG** |
| --- | --- | --- | --- | --- | --- | --- | --- | --- | --- | --- | --- | --- | --- | --- |
| **SW** | 0 | 1 | 5 | 5 | 5 | 1 | 2 | 5 | 5 | 5 | 2 | 5 | 5 | 5 |
| **W** | 1 | 0 | 5 | 5 | 5 | 2 | 1 | 5 | 5 | 5 | 2 | 5 | 5 | 5 |
| **E** | 5 | 5 | 0 | 5 | 5 | 5 | 5 | 1 | 5 | 5 | 5 | 5 | 5 | 5 |
| **B** | 5 | 5 | 5 | 0 | 1 | 5 | 5 | 5 | 1 | 2 | 5 | 5 | 2 | 5 |
| **SB** | 5 | 5 | 5 | 1 | 0 | 5 | 5 | 5 | 2 | 1 | 5 | 5 | 2 | 5 |
| **XOR-SW** | 1 | 2 | 5 | 5 | 5 | 0 | 1 | 5 | 5 | 5 | 2 | 5 | 5 | 5 |
| **XOR-W** | 2 | 1 | 5 | 5 | 5 | 1 | 0 | 5 | 5 | 5 | 3 | 5 | 5 | 5 |
| **XOR-E** | 5 | 5 | 1 | 5 | 5 | 5 | 5 | 0 | 5 | 5 | 5 | 5 | 5 | 5 |
| **XOR-B** | 5 | 5 | 5 | 1 | 2 | 5 | 5 | 5 | 0 | 1 | 5 | 5 | 3 | 5 |
| **XOR-SB** | 5 | 5 | 5 | 2 | 1 | 5 | 5 | 5 | 1 | 0 | 5 | 5 | 2 | 5 |
| **X-SW** | 1 | 2 | 5 | 5 | 5 | 2 | 3 | 5 | 5 | 5 | 0 | 5 | 5 | 5 |
| **X** | 5 | 5 | 5 | 5 | 5 | 5 | 5 | 5 | 5 | 5 | 5 | 0 | 5 | 5 |
| **X-SB** | 5 | 5 | 5 | 2 | 1 | 5 | 5 | 5 | 3 | 2 | 5 | 5 | 0 | 5 |
| **NG** | 5 | 5 | 5 | 5 | 5 | 5 | 5 | 5 | 5 | 5 | 5 | 5 | 5 | 0 |

The matrix lists various comparative preference categories such as SW (Strong Worse), W (Worse), E (Equal), B (Better), SB (Strong Better), and several XOR and X modified categories along with NG (Non-Gradable). Each cell in the matrix shows a weight from 0 to 5, indicating the level of disagreement between pairs of categories:

- **0** indicates no disagreement (same category).
- **1-2** indicates slight to moderate disagreement.
- **3-4** indicates significant disagreement.
- **5** indicates maximum disagreement (completely opposite or unrelated categories).

Below are detailed analyses of the disagreements and alignments within the preference categories as outlined in the Matrix:

- **Direct Comparisons (SW, W, B, SB):** Comparisons within this group (e.g., SW vs. W, B vs. SB) show low to moderate disagreements (1-2) except when compared with their direct opposites (e.g., SW vs. B, W vs. SB) where the disagreement is maximum (5). This reflects the clear conceptual differences in the direction of preference.
- **Neutral Category (E):** E (Equal) consistently has high disagreement (5) with all directional preferences (SW, W, B, SB) indicating its distinct nature as a neutral category where no comparative preference is implied.
- **XOR Categories:** These categories (XOR-SW, XOR-W, XOR-B, XOR-SB) typically show moderate disagreements with their non-XOR counterparts (1-2) and high disagreements (5) with categories that have opposite directions. The slight disagreements between similar XOR and non-XOR categories suggest that XOR adds a layer of conditionality but does not entirely change the fundamental comparative nature.
- **X Categories:** X and X-SW, X-SB show a consistent pattern of maximum disagreement (5) with all other categories except when compared to themselves (0) or similar types (low to moderate). This indicates the complex nature of these categories in comparative contexts.
- **Non-Gradable (NG):** NG shows maximum disagreement (5) with all other categories, reflecting its role in capturing comparative questions that do not imply any hierarchical or qualitative comparison.

This matrix serves as the foundation for calculating the Weighted Kappa statistic, facilitating a nuanced analysis of annotator consistency that goes beyond mere agreement, to also consider the relative impact of different types of disagreements on the overall reliability of the data.

By applying the Weighted Kappa statistic grounded in this comprehensive weighted disagreement matrix, we offer a sophisticated measure of agreement that aligns with the complex, multi-dimensional nature of comparative preferences in subjective questions. This methodology not only highlights the high degree of consensus among annotators in identifying and classifying a wide array of preferences but also underscores the robustness of SCRQD dataset for subsequent analyses of comparative relations.

5. Please note that PLOS ONE has specific guidelines on code sharing for submissions in which author-generated code underpins the findings in the manuscript. In these cases, all author-generated code must be made available without restrictions upon publication of the work. Please review our guidelines at https://journals.plos.org/plosone/s/materials-and-software-sharing#loc-sharing-code and ensure that your code is shared in a way that follows best practice and facilitates reproducibility and reuse.

**Response to Editorial Comment on Code Sharing Guidelines:**

Dear Editor,

We sincerely appreciate your clarification regarding PLOS ONE's specific guidelines concerning the sharing of author-generated code. Recognizing the significance of code sharing in ensuring the reproducibility and utilization of our research outcomes, we have taken prompt action to align with these guidelines.

In adherence to PLOS ONE's code sharing policy, detailed at https://journals.plos.org/plosone/s/materials-and-software-sharing#loc-sharing-code, we have implemented the following measures:

1. **Code and Data Availability and Repository:** Our code and data have been uploaded to a GitHub repository, a widely recognized and accessible platform for code sharing within the scientific community. **The dataset and codes can be accessed at** **https://github.com/mahsamb/ SCRQD.** This includes comprehensive scripts for our model, thereby ensuring complete transparency and facilitating the reproducibility of our findings.
2. **Documentation and Licensing:** Our code is thoroughly documented to facilitate its comprehension and utilization by others. Additionally, we have included a clear license specifying the terms under which our code can be utilized, thereby ensuring compliance with PLOS ONE's guidelines for unrestricted access.
3. **Best Practices for Code Sharing:** We have meticulously reviewed and adhered to best practices in code sharing to bolster the reproducibility of our work. This encompasses detailed comments within the code, a comprehensive README file, and ensuring the code is structured and organized for clarity and accessibility.

We firmly believe that by making our code readily accessible, we are contributing to the openness and reproducibility of scientific research. We remain steadfast in our commitment to upholding PLOS ONE's standards and are grateful for the journal's dedication to transparent and open science practices.

Thank you for your guidance on this matter. We stand prepared to make any further adjustments necessary to ensure full compliance with the journal's requirements.

6. We note that your Data Availability Statement is currently as follows: All relevant data are within the manuscript and its Supporting Information files.

Please confirm at this time whether or not your submission contains all raw data required to replicate the results of your study. Authors must share the &ldquo;minimal data set&rdquo; for their submission. PLOS defines the minimal data set to consist of the data required to replicate all study findings reported in the article, as well as related metadata and methods (https://journals.plos.org/plosone/s/data-availability#loc-minimal-data-set-definition).

For example, authors should submit the following data:

- The values behind the means, standard deviations and other measures reported;
- The values used to build graphs;
- The points extracted from images for analysis.

Authors do not need to submit their entire data set if only a portion of the data was used in the reported study.

If your submission does not contain these data, please either upload them as Supporting Information files or deposit them to a stable, public repository and provide us with the relevant URLs, DOIs, or accession numbers. For a list of recommended repositories, please see https://journals.plos.org/plosone/s/recommended-repositories.

If there are ethical or legal restrictions on sharing a de-identified data set, please explain them in detail (e.g., data contain potentially sensitive information, data are owned by a third-party organization, etc.) and who has imposed them (e.g., an ethics committee). Please also provide contact information for a data access committee, ethics committee, or other institutional body to which data requests may be sent. If data are owned by a third party, please indicate how others may request data access.

**Response to Editorial Comment 6: Dataset Description and Compliance:**

Dear Editor,

We appreciate your attention to the guidelines outlined by PLOS for data transparency and replicability. In accordance with these guidelines, we have taken substantial measures to ensure the comprehensive deposition and public availability of all raw data supporting the findings of our study.

Demonstrating our commitment to promoting open science practices, we have chosen to host the dataset on GitHub, a platform widely recognized for its accessibility and reliability within the scientific community. This repository serves as a repository for not only the raw data essential for replicating the results presented in our manuscript but also detailed metadata and methodological descriptions, facilitating thorough comprehension and reuse.

**For easy access, the dataset, along with pertinent scripts and resources, is accessible via our dedicated GitHub repository at https://github.com/mahsamb/ SCRQD.** We firmly believe that such transparency and openness will catalyze further research and collaboration within our field.

We are grateful for your guidance in refining our manuscript, and we eagerly anticipate any additional suggestions and feedback you may offer.

7. Please include captions for your Supporting Information files at the end of your manuscript, and update any in-text citations to match accordingly. Please see our Supporting Information guidelines for more information: http://journals.plos.org/plosone/s/supporting-information.

**Responding to an editor's comment regarding the inclusion of captions for Supporting Information files**

Dear Editor,

Thank you for your guidance on enhancing the reproducibility of our results. In accordance with your recommendations, we have uploaded the relevant datasets and code to GitHub to further support transparency and facilitate the reproducibility of our findings. This GitHub repository provides access to all computational scripts, data files, and detailed documentation necessary to replicate our analyses and experiments. The repository can be accessed at the following URL: https://github.com/mahsamb/ SCRQD.

We trust that these efforts align with the journal's requirements for supporting information and data sharing. Please inform us if there are any additional modifications or information required.

Thank you once again for the opportunity to enhance our manuscript.

**Conclusion Response to the Editor:**

In conclusion, we have diligently revised our manuscript, meticulously considering each of the constructive comments provided by Reviewer #1. These revisions have not only effectively addressed the concerns raised but have also enhanced the depth and clarity of our research. We are confident that our manuscript now offers a more comprehensive and lucid contribution to the field of Natural Language Processing (NLP) and Question Answering (QA).

Thank you for your valuable feedback and guidance throughout this process.

//////////////////////////////////////////////////////////////////////////////////////////////////////////////////////

**Opening Response to the Reviewers:**

Dear Reviewer,

Thank you for your insightful comments and the constructive critique provided. We're grateful for your thorough review and the chance to refine the sections you've highlighted for improvement. Our dedication lies in improving our manuscript's quality and transparency, and we have accordingly updated it based on your suggestions.

[Note: HTML markup is below. Please do not edit.]

Reviewers&#39; comments:

Reviewer&#39;s Responses to Questions

**Comments to the Author**

1. Is the manuscript technically sound, and do the data support the conclusions?

The manuscript must describe a technically sound piece of scientific research with data that supports the conclusions. Experiments must have been conducted rigorously, with appropriate controls, replication, and sample sizes. The conclusions must be drawn appropriately based on the data presented.

Reviewer #1: Partly

**Response to Reviewer #1's Comment on Technical Soundness and Data Support**

Dear Reviewer,

Thank you for your feedback. We will thoroughly review our experimental methodologies and data presentation to ensure they robustly support our conclusions.

2. Has the statistical analysis been performed appropriately and rigorously?

Reviewer #1: No

**Response to Reviewer's Comment on Statistical Analysis**

Dear Reviewer,

Thank you for your comment. We will re-evaluate and strengthen our statistical analysis to ensure its appropriateness and rigor, addressing your concerns in the revision.

3. Have the authors made all data underlying the findings in their manuscript fully available?

The [PLOS Data policy](http://www.plosone.org/static/policies.action#sharing) requires authors to make all data underlying the findings described in their manuscript fully available without restriction, with rare exception (please refer to the Data Availability Statement in the manuscript PDF file). The data should be provided as part of the manuscript or its supporting information, or deposited to a public repository. For example, in addition to summary statistics, the data points behind means, medians and variance measures should be available. If there are restrictions on publicly sharing data&mdash;e.g. participant privacy or use of data from a third party&mdash;those must be specified.

Reviewer #1: Yes

**Response to Reviewer's Comment on Data Availability**

Dear Reviewer,

Thank you for recognizing the completeness of the data supporting our study's results. We value your acknowledgment, which reflects our dedication to complying with the PLOS Data policy.

4. Is the manuscript presented in an intelligible fashion and written in standard English?

PLOS ONE does not copyedit accepted manuscripts, so the language in submitted articles must be clear, correct, and unambiguous. Any typographical or grammatical errors should be corrected at revision, so please note any specific errors here.

Reviewer #1: Yes

**Response to Reviewer's Comment on Manuscript Presentation and Language Quality**

Dear Reviewer,

We appreciate your positive feedback on the clarity and language quality of our manuscript. It is encouraging to hear that our work meets the linguistic standards set by PLOS ONE.

////////////////////////////////////////////////////////////////////////////////////////////////////////////

5. Review Comments to the Author

Please use the space provided to explain your answers to the questions above. You may also include additional comments for the author, including concerns about dual publication, research ethics, or publication ethics. (Please upload your review as an attachment if it exceeds 20,000 characters)

Reviewer #1: The authors present a new dataset, denoted by the acronym SCRQD, containing
1,275 subjective comparative questions from the smartphone domain. Inspired
by (Liu et al, 2021), the questions are translated into (sets of)
quintuples denoting subject entity, object entity, compared aspect,
constraint, and preference. Together with the SCRQD dataset, a new SCRQE
system for extracting these quintuples from the input questions is
presented and evaluated.

The SCRQE system is designed as a pipeline with three stages that are
evaluated separately as well as in combination. The performance is partly
compared to other similar approaches although the details of the comparison
are not always clear.

Dear Reviewer,

Thank you for summarizing the core contributions of our manuscript, including the introduction of the SCRQD dataset and the SCRQE system for quintuple extraction from subjective comparative questions within the smartphone domain. We acknowledge your point regarding the clarity of our comparative analysis with similar approaches. In response, we will revisit this section to enhance the clarity of the comparisons, ensuring a more transparent and detailed presentation of how our system stands in relation to existing methodologies. We are committed to providing a clearer context and justification for our approach's effectiveness and uniqueness.

////////////////////////////////////////////////////////////////////////////////////////////////////////////

A major drawback of the text lies in exaggerative claims about the
uniqueness of the presented approach which then result in the fuzzy
comparison. The authors claim that &quot;there is no publicly available dataset
to derive subjective comparative relations from the questions&quot;. This is
definitely not true as comparatives in NLP are studied for decades and
simple search reveals tens of works in this regard.

Dear Reviewer,

Thank you for your valuable feedback. We appreciate you raising this critical point.

We acknowledge that our initial statement regarding the non-existence of relevant datasets might be an overstatement and apologize for any confusion it may have caused.

Here's a more nuanced perspective on existing datasets:

- **Focus on Subjective Comparison:** While research on comparative question answering in NLP is extensive, identifying publicly available datasets that specifically capture subjective comparisons within the questions remains a challenge. Many existing datasets:
  - **Lack a focus on subjectivity:** They not capture the user's preference intensity of the comparison.
  - **Don't target the smartphone domain:** Their focus on general topics makes them unsuitable for our specific research area.
- **Addressing the Shortcomings of the Relevant Dataset:** The existing dataset by Yu et al. [2] while relevant, has limitations:
  - **Limited Size**: It only includes 35 subjective comparative questions, making it statistically insignificant for our analysis.
  - **Lack of Annotations:** It lacks annotations for crucial elements like preference intensity and constraints, hindering our ability to extract the necessary information.
  - **Single-Sentence Structure:** The dataset is confined to single-sentence questions, potentially failing to capture the complexity of real-world user queries.
  - **Lack of Support for Multiple Relations:** It does not accommodate multiple relations per question, limiting its usefulness for analyzing the complex comparative interactions our research aims to address."
- **The Need for High-Quality Annotation:** Given the fact that subjective comparative relation extraction is inherently subjective, high inter-annotator agreement scores are crucial for ensuring the reliability and quality of the datasets. As an initial step and considering the costs associated with detailed annotation, we have built a small but high-quality dataset specifically designed to push the boundaries of analysis in this area of research. This strategic focus on quality over quantity ensures that our dataset provides a solid foundation for developing and testing our model, addressing a critical gap in the current research landscape.

Therefore, while acknowledging the broader field of comparative question answering, we believe that focusing on subjective comparative questions presents a unique challenge. There is a scarcity of publicly available annotated datasets that fully meet our specific research needs. In response to the concerns raised, we have taken proactive steps to enhance the rigor and transparency of our approach.

- **Moving Forward:** We will revise our manuscript to:
  - Modify our statement to accurately reflect the limitations of existing datasets in addressing our research needs (**in revised section: 2. Related Work**).
  - Highlight the uniqueness of our approach by emphasizing that our work addresses the limitations of existing datasets and offers new insights within the focused area of subjective comparisons in the smartphone domain (**in revised section: 2. Related Work**)
  - Inter-rater agreement annotations were conducted for each part of our dataset during the initial stages of our research. While these were not explicitly detailed in the first version of our manuscript, based on your valuable feedback, we will now include these details in the revised manuscript to highlight the reliability and validity of our data collection process (**in revised sub-section: Annotation Process and Inter-Annotator Agreement Analysis**).

We appreciate your feedback and believe it will significantly improve the clarity and accuracy of our research. We are committed to providing a comprehensive examination of the dataset landscape while accurately reflecting the novelty and contribution of our work.

**Revised Section 3.2.2. Data Quality and Integrity**

Maintaining high standards for data quality and integrity is crucial for the creation and effectiveness of the dataset. This section discusses the methods used to develop a dataset that supports the needs of NLP research effectively. By employing thorough dataset preparation techniques, incorporating distinctive attributes, implementing a meticulously planned annotation procedure, and applying rigorous criteria for assessing inter-annotator consistency, we adopt a strategy that emphasizes accuracy, dependability, and ethical practices in constructing the dataset.

**Dataset Creation: Detailed Preparation Methods and Specifications**

This section offers a thorough explanation of the careful steps and criteria involved in creating the SCQRD dataset, which is fundamental to its construction. It delves into the methodology used, the selection and modification process of data, and the strategic planning behind compiling the dataset to guarantee its integrity and usefulness for intended research purposes.

- **Origin and Assembly of Data:** We started with data inspired by user contributions on platforms like Amazon (www.amazon.com) and Quora (www.quora.com), focusing on the smartphone domain. Finding questions that met our specific needs (subjective comparative questions) was difficult, as we required questions to clearly mention the entities being compared, be precisely phrased, and fit within our set categories.
- **Development and Enhancement of the SCQRD Dataset:** Due to the scarcity of questions that exactly matched our research criteria, we modified existing questions and formulated new ones to diversify our dataset. This process involved:
  - **Sentence Modification:** Changing existing sentences into questions that fit our research.
  - **Adjusting Content:** Adding, deleting, or modifying parts of questions to increase their specificity and applicability.
  - **Inventing Data:** Adding fictional yet realistic questions to expand the dataset’s coverage of potential scenarios and queries.
- **Verification and Endorsement Process:** The revised and newly formulated questions underwent a strict validation process, managed by experts with knowledge in the smartphone field's writing and conceptual aspects. Their endorsement confirmed the dataset's relevance and alignment with our research aims.
- **Strategies for Reducing Bias in Dataset Assembly:** To minimize bias in question selection and presentation, we adopted several strategies tailored to question classification tasks. First, we gathered questions from a variety of online sources, not just mainstream platforms, to ensure a diverse range of question styles and subjects, thereby reducing selection bias. Next, in modifying and generating questions, we made sure to cover various smartphone categories to reflect a wide array of user inquiries and needs, aiming to lessen content bias. To combat annotator bias, detailed guidelines and regular calibration sessions were employed to ensure consistent and neutral question classification.
- **Ethical and Compliance Measures:** All processes of data collection, modification, and creation were performed in strict adherence to the terms and conditions of the original platforms. Ethical considerations were a top priority, including the avoidance of any personally identifiable information to protect the privacy and confidentiality of individuals involved in the initial data gathering.

**Annotation Process and Inter-Annotator Agreement Analysis**

This study leveraged the expertise of three annotators with backgrounds in computational linguistics and specializations in product domain sentiment analysis. Prior to annotation, the team underwent a comprehensive training session focused on the intricacies of subjective comparative questions. The training included an extensive review of key concepts and definitions relevant to comparative relations, such as entities, aspects, constraints, and comparative preferences. This ensured a shared understanding and application of the annotation guidelines. The complexity of identifying subject entities, object entities, and compared aspects within multiple comparative relations was emphasized due to their critical role in the task.

The annotation process was designed to be iterative, allowing annotators to independently label a shared set of questions before convening to discuss discrepancies and refine their interpretations of the guidelines. This collaborative approach facilitated a dynamic refinement of annotation practices, enhancing consistency across the dataset.

To quantify the level of agreement among the annotators, we employed a multifaceted approach, utilizing several inter-annotator agreement (IAA) metrics tailored to the specific aspects of the annotation task:

- **Fleiss' Kappa [24]:** This metric was chosen to assess the agreement on the identification of entities and aspects, which are foundational to constructing the comparative relations. Given its suitability for categorical data and its accommodation of multiple raters, Fleiss' Kappa provided a robust measure of consensus for these critical components.
- **Weighted Kappa [25]:** To account for the ordinal nature of comparative preferences, encompassing categories like "Better" to "XOR-Strong Worse," Weighted Kappa was employed. This metric assigns different weights to disagreements based on the severity within preference categories, offering a more nuanced assessment of annotator consistency in this intricate judgment domain.
- **Alpha U [26]:** To evaluate the agreement on extracting entire comparative relations, including Subject Entity, Object Entity, Compared Aspect, Constraint, and Comparative Preference, Alpha U was chosen. This metric is an adaptation of Krippendorff's Alpha [27] specifically designed for tasks involving segmenting and classifying text. By employing Alpha U, the study accurately measured annotator agreement on both the identification and contextual classification of these relations.

High inter-annotator agreement is essential for validating the reliability of the annotated dataset, especially in the complex domain of subjective comparative questions. This section summarizes the agreement metrics, reflecting the effectiveness of the annotation process across various components.

- **Entity and Aspect Identification:** The annotation process yielded a Fleiss' Kappa score of 0.94 for entity identification and 0.89 for aspect identification. These scores, indicating almost perfect agreement, attest to the effectiveness of our annotation training and guidelines in achieving a high degree of consistency among annotators for these fundamental components.
- **Constraint Recognition:** The Fleiss' Kappa score for constraint identification was 0.78, signifying substantial agreement. The slightly lower score compared to entity and aspect identification reflects the inherent challenges in recognizing and categorizing constraints, which often require a deeper inference of the question context.
- **Subject Entity, Object Entity, and Compared Aspect Identification:** The IAA metrics underscore the consistency in annotator judgments across key components, with Fleiss' Kappa scores revealing: Subject Entities at 95%, indicating almost perfect agreement; Object Entities at 92%, demonstrating substantial consensus; and Compared Aspects at 89%, reflecting significant agreement. These scores collectively highlight the effectiveness of our annotator training and guidelines in ensuring high-quality, consistent annotations for the foundational elements of comparative relation extraction.
- **Comparative Preference Classification:** The application of Weighted Kappa resulted in a score of 0.76 for comparative preferences. This substantial agreement level highlights the annotators' ability to navigate the intricate spectrum of preferences, supported by the detailed categorization scheme developed for this task.
- **Comparative Relation Extraction:** The comprehensive approach to annotating complete comparative relations was reflected in an Alpha U score of 0.80. This high level of agreement among annotators underscores the effectiveness of our iterative annotation process and collaborative discussions in clarifying complex relations, including the accurate identification of Subject Entities, Object Entities, and Compared Aspects.

The inter-annotator agreement analysis revealed a high level of consensus across all components of the annotation task, validating the reliability of the annotated dataset for extracting subjective comparative relations. While the process identified areas for improvement, particularly in constraint recognition, the overall results affirm the robustness of the annotation methodology. The collaborative and iterative nature of the annotation process, along with the targeted use of multiple IAA metrics, has established a strong foundation for subsequent analyses and model development based on this dataset.

**Addressing Disagreements among Annotators**

Despite the overall high levels of agreement, instances of disagreement among annotators served as valuable opportunities for further refining our annotation process and guidelines. Disagreements primarily arose in two areas: constraint recognition and the classification of nuanced comparative preferences.

To address these disagreements, we implemented a multifaceted resolution strategy, which included:

- **Guideline Refinement:** Disagreement analysis identified areas for improved clarity within the annotation guidelines. For constraint recognition, additional examples of explicit constraints were incorporated to ensure consistent identification. Similarly, comparative preferences received more detailed definitions and examples, particularly for complex categories like "X-types," "XOR-types," and "Non-Gradable." These refinements aim to achieve a shared understanding among annotators.
- **Focused Annotator Training:** Following guideline refinement, additional training sessions were held, focusing specifically on areas where disagreements were most prevalent. These sessions included interactive discussions on challenging examples, allowing annotators to explore and reconcile different interpretations of the guidelines.
- **Consensus Meetings:** For questions that continued to pose challenges after the initial annotation and review, consensus meetings were convened. During these meetings, annotators discussed their rationales for their classifications, with a senior member of the research team facilitating a consensus decision. This collaborative approach not only resolved individual instances of disagreement but also contributed to the continuous improvement of the annotation process.

These resolution strategies led to a marked decrease in the rate of disagreement in subsequent annotation rounds. Specifically:

- **Constraint Recognition:** The refinement of guidelines and focused training resulted in an increase in the Fleiss' Kappa score for constraint recognition from 0.78 to 0.82, indicating enhanced agreement and a more consistent application of the guidelines.
- **Comparative Preference Classification:** The consensus meetings and enriched annotation examples for comparative preferences improved the Weighted Kappa score from 0.76 to 0.81, reflecting a higher level of annotator consistency in this complex judgment area.
- **Holistic Enhancement across Annotations:** The improvements in Comparative Preference Classification (CPC) and Constraint Extraction, coupled with the meticulous clarification of Subject Entities, Object Entities, and Compared Aspects, collectively led to an overarching enhancement in SCRQD dataset's annotation quality. The observed improvement in the Alpha U score from 0.80 to 0.84 highlights the significant impact of the implemented resolution strategies. These strategies have facilitated the more accurate, reliable, and comprehensive extraction of comparative relations.

The iterative process of addressing annotator disagreements through guideline refinement, focused training, and consensus discussions proved instrumental in enhancing the reliability of our annotated dataset. Not only did this approach resolve specific instances of disagreement, but it also fostered a deeper collective understanding among the annotators, contributing to the overall quality and coherence of the dataset. These efforts underscore the dynamic nature of the annotation process and highlight the importance of continuous engagement and adaptation in achieving high-quality annotations for complex NLP tasks.

**Methodology for Assessing Annotator Agreement on Comparative Preferences**

To quantify the inter-annotator agreement on the Comparative Preference Classification (CPC) within the SCRQD dataset, we employed the Weighted Kappa statistic. This statistical method is particularly effective due to its ability to account for the ordinal nature of the data and the varying degrees of disagreement between different types of comparative preferences.

**Table 12.** Construction of the Weighted Disagreement Matrix

|  | **SW** | **W** | **E** | **B** | **SB** | **XOR-SW** | **XOR-W** | **XOR-E** | **XOR-B** | **XOR-SB** | **X-SW** | **X** | **X-SB** | **NG** |
| --- | --- | --- | --- | --- | --- | --- | --- | --- | --- | --- | --- | --- | --- | --- |
| **SW** | 0 | 1 | 5 | 5 | 5 | 1 | 2 | 5 | 5 | 5 | 2 | 5 | 5 | 5 |
| **W** | 1 | 0 | 5 | 5 | 5 | 2 | 1 | 5 | 5 | 5 | 2 | 5 | 5 | 5 |
| **E** | 5 | 5 | 0 | 5 | 5 | 5 | 5 | 1 | 5 | 5 | 5 | 5 | 5 | 5 |
| **B** | 5 | 5 | 5 | 0 | 1 | 5 | 5 | 5 | 1 | 2 | 5 | 5 | 2 | 5 |
| **SB** | 5 | 5 | 5 | 1 | 0 | 5 | 5 | 5 | 2 | 1 | 5 | 5 | 2 | 5 |
| **XOR-SW** | 1 | 2 | 5 | 5 | 5 | 0 | 1 | 5 | 5 | 5 | 2 | 5 | 5 | 5 |
| **XOR-W** | 2 | 1 | 5 | 5 | 5 | 1 | 0 | 5 | 5 | 5 | 3 | 5 | 5 | 5 |
| **XOR-E** | 5 | 5 | 1 | 5 | 5 | 5 | 5 | 0 | 5 | 5 | 5 | 5 | 5 | 5 |
| **XOR-B** | 5 | 5 | 5 | 1 | 2 | 5 | 5 | 5 | 0 | 1 | 5 | 5 | 3 | 5 |
| **XOR-SB** | 5 | 5 | 5 | 2 | 1 | 5 | 5 | 5 | 1 | 0 | 5 | 5 | 2 | 5 |
| **X-SW** | 1 | 2 | 5 | 5 | 5 | 2 | 3 | 5 | 5 | 5 | 0 | 5 | 5 | 5 |
| **X** | 5 | 5 | 5 | 5 | 5 | 5 | 5 | 5 | 5 | 5 | 5 | 0 | 5 | 5 |
| **X-SB** | 5 | 5 | 5 | 2 | 1 | 5 | 5 | 5 | 3 | 2 | 5 | 5 | 0 | 5 |
| **NG** | 5 | 5 | 5 | 5 | 5 | 5 | 5 | 5 | 5 | 5 | 5 | 5 | 5 | 0 |

The matrix lists various comparative preference categories such as SW (Strong Worse), W (Worse), E (Equal), B (Better), SB (Strong Better), and several XOR and X modified categories along with NG (Non-Gradable). Each cell in the matrix shows a weight from 0 to 5, indicating the level of disagreement between pairs of categories:

- **0** indicates no disagreement (same category).
- **1-2** indicates slight to moderate disagreement.
- **3-4** indicates significant disagreement.
- **5** indicates maximum disagreement (completely opposite or unrelated categories).

Below are detailed analyses of the disagreements and alignments within the preference categories as outlined in the Matrix:

- **Direct Comparisons (SW, W, B, SB):** Comparisons within this group (e.g., SW vs. W, B vs. SB) show low to moderate disagreements (1-2) except when compared with their direct opposites (e.g., SW vs. B, W vs. SB) where the disagreement is maximum (5). This reflects the clear conceptual differences in the direction of preference.
- **Neutral Category (E):** E (Equal) consistently has high disagreement (5) with all directional preferences (SW, W, B, SB) indicating its distinct nature as a neutral category where no comparative preference is implied.
- **XOR Categories:** These categories (XOR-SW, XOR-W, XOR-B, XOR-SB) typically show moderate disagreements with their non-XOR counterparts (1-2) and high disagreements (5) with categories that have opposite directions. The slight disagreements between similar XOR and non-XOR categories suggest that XOR adds a layer of conditionality but does not entirely change the fundamental comparative nature.
- **X Categories:** X and X-SW, X-SB show a consistent pattern of maximum disagreement (5) with all other categories except when compared to themselves (0) or similar types (low to moderate). This indicates the complex nature of these categories in comparative contexts.
- **Non-Gradable (NG):** NG shows maximum disagreement (5) with all other categories, reflecting its role in capturing comparative questions that do not imply any hierarchical or qualitative comparison.

This matrix serves as the foundation for calculating the Weighted Kappa statistic, facilitating a nuanced analysis of annotator consistency that goes beyond mere agreement, to also consider the relative impact of different types of disagreements on the overall reliability of the data.

By applying the Weighted Kappa statistic grounded in this comprehensive weighted disagreement matrix, we offer a sophisticated measure of agreement that aligns with the complex, multi-dimensional nature of comparative preferences in subjective questions. This methodology not only highlights the high degree of consensus among annotators in identifying and classifying a wide array of preferences but also underscores the robustness of SCRQD dataset for subsequent analyses of comparative relations.

////////////////////////////////////////////////////////////////////////////////////////////////////////////

Moreover, the presented approach is not specific to questions at all
despite the authors stating the opposite. They do not link or offer linkage
to possible answers and the RoBERTa-based analysis does not use anything
different from standard comparative sentence analysis. Other (mostly)
ignored sources of comparison are numerous aspect identification datasets
and approaches, although some are briefly mentioned in the evaluation of
the first stage of the system.

Dear Reviewer,

Thank you for your valuable insights and for highlighting the areas of concern regarding the perceived specificity of our approach to processing subjective comparative questions, as well as the apparent overlap with standard comparative sentence analysis methodologies. Additionally, your comments on the potential underutilization of aspect identification datasets and methodologies in our study have prompted a thorough reassessment and subsequent enhancement of our approach.

To address the critiques raised and further clarify our methodology:

- **Refining Our Approach for Enhanced Specificity in Comparative Question Analysis:** In the revised version of our manuscript, we directly address the need for enhanced model specificity towards analyzing subjective comparative questions **by integrating the "AdapterHub/roberta-base-pf-qnli" adapter into our SCRQE model (Revised Section 4.2. Element Extraction (EE) Evaluation).** This adaptation, specifically designed for question-answering tasks, significantly boosts the model's capability to discern the complexities and subtleties of comparative queries. It not only amplifies the model's adaptability across various question formulations but also sharpens its focus on extracting and analyzing comparative elements accurately. Additionally, this integration enhances the model's efficiency, streamlining the analysis process without extensive retraining, thereby optimizing computational resources. This strategic enhancement is pivotal in advancing our model's performance, underscoring our commitment to achieving high precision in the nuanced field of comparative question analysis.
- **Specialization in Question Analysis:** We have specialized our question analysis through the strategic use of the adapter and a focused Comparative Preference Classification (CPC) analysis, tailored specifically to question contexts rather than sentences. This specialization is evident in our incorporation of question-specific classifications, such as x-types and xor-types, which are uniquely relevant to the domain of subjective comparative questions. This approach not only demonstrates our commitment to addressing the specific challenges posed by subjective comparative analysis but also significantly distinguishes our methodology from standard comparative sentence analysis.
- **Incorporating Aspect Identification Resources**: In our pursuit to develop a robust model for aspect extraction targeted at subjective comparative questions, we initially utilized the RoBERTa framework. It's crucial to note that for comparing our model's aspect extraction capabilities with sentence-based datasets, we specifically relied on the foundational aspects of our model, excluding enhancements from multi-task learning and adapters. This decision was driven by the need to establish a baseline using datasets like SemEval 2014, which, despite their relevance for aspect extraction, lack detailed annotations for comprehensive element extraction in question domains. Thus, our comparison aimed to showcase the inherent strengths of the RoBERTa model in its basic configuration. This expansion not only enriches our evaluation but also positions our methodology within a more diverse analytical landscape, emphasizing our model's capability to address the intricacies of subjective comparative questions even without advanced modifications.

These methodological enhancements and clarifications, inspired by your insightful feedback, are aimed at reinforcing the distinctiveness of our approach, delineating its capabilities beyond conventional comparative sentence analysis, and underscoring our dedication to methodological refinement and expansion. We are immensely grateful for the opportunity to refine our manuscript and methodology, and we eagerly anticipate further guidance and feedback.

**To honor the reviewer's input and ensure the thoroughness of our response, we also include this addition here in our response letter:**

**Revised Section 4.2. Element Extraction (EE) Evaluation**

We assessed the Element Extraction (EE) task, encompassing three subtasks: Entity Extraction ($T_{1}$), Aspect Extraction ($T_{2}$), and Constraint Extraction ($T_{3}$). Each subtask is initially evaluated individually, followed by the evaluation of the multi-task learning (MTL) EE model and a subsequent comparison with other methods. Evaluations were performed using the SCQRD dataset, employing stratified 5-fold cross-validation to ensure unbiased analysis. The results, which focus on Precision, Recall, and F-score metrics, are detailed in Table 17. This table shows comparisons between single-task learning (STL), MTL, and the adapter-enhanced MTL (MTL + Adapter) models.

The STL model, serving as our baseline with a fine-tuned RoBERTa-base configuration, demonstrates robust performance with F-scores of 94.9% in Entity Extraction, 81.2% in Aspect Extraction, and 73.1% in Constraint Extraction. These results indicate that while the model provides a solid foundation, there is potential for further enhancement in managing the complex interdependencies among Entity, Aspect, and Constraint tasks.

In contrast, the MTL model showed mixed performance changes compared to the STL model: a decrease of 2.5% for Entity Extraction, an increase of 8% for Aspect Extraction, and a decrease of 0.7% for Constraint Extraction. This underscores the MTL approach's general effectiveness, particularly in enhancing Aspect Extraction, though some challenges remain in Entity and Constraint Extraction.

Table 17 also outlines the performance enhancements of the MTL model with the integration of an adapter. The adapter-enhanced MTL model (MTL + Adapter) exhibited further improvements: an increase of 1.3% for Entity Extraction, a slight decrease of 0.6% for Aspect Extraction, and an increase of 7.1% for Constraint Extraction. These results highlight the critical role of the adapter in refining the model’s capabilities, particularly in handling complex comparative element extractions more efficiently and effectively. The strategic integration of the AdapterHub/roberta-base-pf-qnli adapter, specifically designed for question-answering tasks, significantly bolsters the model’s ability to discern the intricacies and subtleties of comparative queries, leading to notable improvements in adaptability and accuracy across various question formulations.

In Fig 5, we present the average F-scores for each approach—STL, MTL, and MTL with Adapter—across the three subtasks of Element Extraction: Entity Extraction, Aspect Extraction, and Constraint Extraction. This averaging method provides a comprehensive view of overall performance in Element Extraction, illustrating the progressive enhancements achieved with each approach. The graph demonstrates that the MTL approach improves upon the STL results, and integrating an adapter into the MTL framework (MTL + Adapter) further enhances the model’s effectiveness, resulting in the highest performance metrics across all evaluated tasks.

The STL Aspect Extraction model is further validated using the SemEval 2014 dataset across the Laptop and Restaurant domains. The results, presented in Tables 18 and 19, indicate the model's state-of-the-art performance in these domains.

Lastly, we briefly mention the methods compared:

- Augustyniak et al. [23] used GloVe embeddings with BiLSTM enhanced by CRF.
- Tran et al. [32] introduced stacked BiGRU-CRF for sentiment features.
- Da’u and Salim [33] implemented a multi-channel CNN using word and POS embeddings.
- Feng et al. [34] developed PECSMT: a model combining BERT, Contextual Summary, and a Transmission network.
- Wu et al. [35] presented DeepWMaxSAT, merging logic knowledge with deep learning for Aspect Extraction.
- Zschornack et al. [36] used a POS-AttWD-BLSTM-CRF architecture.
- Chen and Qian [37] focused on aspect and context term distributions, proposing SoftProto for word associations.
- Wei et al. [38] employed the DE-CNN method for aspect pre-extraction.
- Shi et al. [39] merged pre-trained embeddings with BiLSTM and CRF for aspect term extraction.
- Pour and Jalili [40] proposed CNN+WIN, focusing on specific aspect term windows.
- Wang et al. [41] improved sequence tagger performance with post-processing methods.

**Table 17.** Performance Analysis of Entity Extraction, Aspect Extraction, and Constraint Extraction across Five Runs on the SCQRD Dataset.

| **Task** | **Approach** | **Precision** | **Recall** | **F-score** |
| --- | --- | --- | --- | --- |
| Entity Extraction | STL | 0.943 | 0.955 | 0.949 |
|  | MTL | 0.930 | 0.917 | 0.924 |
|  | MTL + Adapter | 0.938 | 0.934 | **0.937** |
| Aspect Extraction | STL | 0.821 | 0.805 | 0.812 |
|  | MTL | 0.888 | 0.897 | 0.892 |
|  | MTL + Adapter | 0.835 | 0.934 | **0.876** |
| Constraint Extraction | STL | 0.793 | 0.691 | 0.731 |
|  | MTL | 0.764 | 0.747 | 0.724 |
|  | MTL + Adapter | 0.796 | 0.843 | **0.795** |

**Fig 5.** Evaluation of Element Extraction Techniques: STL, MTL, and MTL with Adapter.

////////////////////////////////////////////////////////////////////////////////////////////////////////////

The Related works section offers relatively good overview of the approaches
(although concentrating on methods from 2021 and older) but these
approaches are not compared to the proposed system and the dataset
and no explanation of possible differences is offered.

Dear Reviewer,

Thank you for your constructive feedback regarding the Related Works section of our manuscript. We appreciate your observation about the current scope of literature review, primarily focusing on methods up to 2021, and the lack of direct comparison with our proposed system and dataset.

In response, we will update the Related Works section to include a more comprehensive analysis of recent approaches and explicitly compare them with our model and dataset. This update will also provide insights into the unique contributions and potential differences between our work and existing methods. We aim to ensure that this comparison not only highlights our methodology's novelty but also situates it within the broader context of ongoing research advancements.

**To honor the reviewer's input and ensure the thoroughness of our response, we also include this addition here in our response letter:**

**Revised Section 2. Related Work**

On product review platforms, subjective comparative questions significantly influence user choices. The exploration of Subjective Comparative Relation Extraction (SCRE) has been explored extensively, yet previous research has often leaned more towards sentence-level analyses than questions. This section examines the prominent studies in the field, systematically divided into two main subsections: "Question-level" and "Sentence-level" examinations. To facilitate a comprehensive view, a summary of the related works, categorized by various attributes like language, analysis level, and extraction types, is provided in Section 2.3.

**2.1. Question-Level**

Within the SCRE domain, the extraction of comparative information from questions presents a unique set of challenges. Here, we navigate through foundational works that have explored the complex task of interpreting and understanding comparative subtleties embedded within questions.

Pioneering works on question-level SCRE, such as the AQA system by Moghaddam and Ester [1], introduced a novel approach to opinion-based questions with an emphasis on pattern-matching capabilities, though it encounters difficulties with multiple comparative relations. Similarly, the QA model by Yu et al. [2] enhanced comparative question analysis by utilizing Stanford CRF-NER and SVM but shared a set of challenges with the AQA system, particularly in handling complex comparative dynamics. Our system advances these methodologies by integrating a more sophisticated neural network approach that handles multiple comparative relations more dynamically and reduces the manual effort required for pattern matching and entity recognition. Furthermore, the dataset of Yu et al. [2] is the only existing question-level dataset that bears relevance to ours; however, it exhibits significant limitations. It comprises only 35 subjective comparative questions, which is statistically insignificant for a comprehensive examination. Additionally, it lacks annotations for crucial elements such as preference intensity and constraints, which are essential for extracting the detailed comparative insights our model is designed to analyze. The dataset is confined to single-sentence questions, potentially failing to capture the complexity and nuances of real-world user queries. Most importantly, it does not support multiple comparative relations within a single question or accommodate annotations for preference intensity and constraints, limiting its usefulness for analyzing the complex comparative interactions that our research aims to address. In contrast, our dataset is meticulously curated to include a comprehensive range of comparative questions with detailed annotations, supporting a richer and more nuanced understanding of comparative dynamics across various product categories.

Diverse methodological approaches such as Li et al. [8], which utilize class sequential rules and semantic role labeling, have set a new path for Comparable Entity Extraction. Similarly, REDDY and Mahesh Babu [9] approach, characterized by weakly supervised bootstrapping, makes strides in sequential pattern learning and offers a unique perspective on entity recognition. Compared to these studies, our approach leverages deep learning to automatically infer comparative structures from question data, which improves accuracy and reduces the need for extensive rule-based configurations.

Insights from global research, such as Saelan et al. [10, 11] studies based in the Indonesian context, highlight the subtleties of constraints in comparative questions. While they utilize tools effectively, they encounter challenges with certain aspects of comparative relations. These studies are notable as they are among the few that introduce constraint extraction in comparative analysis. Similar to them, our method also incorporates constraint extraction. However, unlike their model, which does not support the extraction of multiple relations within a single question or the distinct identification of subject and object entities, our model is equipped to handle these tasks, enhancing the depth and accuracy of our comparative analysis. In a similar Liu et al. [12] research provides a comprehensive combination of user-generated content to shed light on the Chinese product competition scene. Although their methodology is impressive, it requires intensive manual effort. In contrast, our model automates much of the process, reducing the need for manual input significantly.

**2.2. Sentence-Level**

While questions offer their complexities, sentence-level analyses stand as a foundation in the SCRE research field. This subsection examines the significant contributions that have influenced the understanding of comparative relations found within comprehensive sentences, extracting elements, and identifying patterns.

Foundational studies in the field include Jindal and Liu [13] focus on SCRE in English product reviews, where they introduced a pre-defined set of comparative quintuples. Their method employed CSR and LSR to extract comparative relations from language patterns. Building on this, Xu et al. [14] proposed a two-level CRF model that accounts for interdependencies, designed to extract comparative relations from diverse sources like Amazon and blogs, and capable of considering multiple relations within a single sentence. Further advancing the field, Arora et al. [15] introduced a deep learning technique utilizing a one-layer BiLSTM with GloVe embeddings to extract comparative elements from 27,000 Amazon comparative sentences, demonstrating BiLSTM’s superiority over traditional Semantic Role Labeling (SRL). Our model employs a more recent deep learning approach by leveraging the robust, transformer-based architecture of RoBERTa, which is finely tuned for complex natural language understanding tasks. This allows our system to effectively discern and classify nuanced comparative preferences across varied linguistic contexts, ensuring a high level of precision in comparative preference classification.

Innovative approaches have significantly advanced the field, as exemplified by Liu et al. [5] introduction of the Comparative Opinion Quintuple Extraction (COQE) task. They developed a sophisticated multi-stage neural network model tailored for comparative opinion mining. Their methodology particularly excelled in identifying multiple comparative relations within a single sentence. For instance, Table 1 demonstrates the COQE task using the example sentence: “G6 has a worse zoom than G7, but G6’s battery is more reliable than G7.” The resulting output comprises two comparative opinion quintuples, delineating <Subject Entity, Object Entity, Comparative Aspect, Comparative Opinion, and Comparative Preference>. Our methodology extends this foundational work by incorporating linguistic analysis techniques that enhance both entity recognition and the extraction of comparative constraints, enabling a more nuanced understanding of comparative preferences and contexts. A significant addition to our approach is the integration of Constraint Extraction and the analysis of intensity preferences—elements not addressed in the Liu et al. [5] work. The inclusion of Constraint Extraction allows for a more nuanced understanding of contextual limitations and criteria influencing comparative relations, thus enhancing the comprehensiveness of our analysis. Furthermore, by analyzing intensity preferences, our approach provides a deeper and more precise understanding of user preferences across varied contexts and comparative scenarios. This significantly broadens the application in CPC. This research notably stands out for its adeptness in recognizing and parsing several comparative relations from a solitary sentence, providing a foundation upon which our enhancements build to deliver a more robust and versatile comparative analysis framework.

Liu et al. [5] also introduced three datasets—Car-COQE, Ele-COQE, and Camera-COQE—with the first two in Chinese and the last one in English, catering to specific product categories within those languages. Our dataset provides substantial enhancements over the Camera-COQE [5] dataset, which is primarily focused on comparative opinion mining within camera-related contexts. Unlike Camera-COQE, our dataset is not limited to a single product category or context; it encompasses subjective comparative questions across a broad spectrum of product categories. This allows for a much richer and diversified analysis of comparative preferences. Our dataset includes detailed annotations for preference intensity and constraints—key elements that are not addressed in the Camera-COQE dataset. These annotations enable a deeper understanding of the contextual factors that influence comparative judgments, enhancing the comprehensiveness and accuracy of our comparative analysis. Furthermore, our dataset supports the examination of multiple comparative relations within a single sentence and incorporates aspects alongside the entities involved, significantly broadening the analytical capabilities of our model. By integrating a wide range of linguistic patterns and enabling robust model training and evaluations across different languages and domains, our dataset not only complements but significantly expands the scope and depth of comparative analysis within the field of natural language processing, surpassing the limitations of the Camera-COQE dataset.

Recent research conducted by Gao et al. [16] introduces a novel approach to Comparative Opinion Quintuple Extraction (COQE) by developing an efficient and highly effective end-to-end solution that enhances the encoding, decoding, and learning aspects of the process. Their method incorporates external syntactic dependency structural features and a dynamic structural pruning (DSP) mechanism to refine and optimize raw syntax structures, thereby improving the modeling of opinion elements. For decoding, they formulate COQE as a set prediction problem and employ a non-autoregressive decoding scheme, which generates all possible quintuples simultaneously in parallel. This approach increases decoding speed while retaining the advantages of a generative model. Advanced training targets such as bipartite matching learning loss, cross-element contrastive loss, and mention boundary learning loss are introduced to enhance interaction learning between quadruples and elements, resulting in significant performance improvements over both pipeline baselines and state-of-the-art joint systems by 2 to 3 points on benchmark datasets. However, disadvantages include the potential complexity of implementing and optimizing the dynamic structural pruning mechanism, reliance on syntactic dependency features which may not always be readily available or accurate—especially in languages with less robust NLP tools—and the increased computational resources required for the non-autoregressive decoding scheme and advanced training losses, which could limit its application in resource-constrained environments. In contrast to Gao et al. [16], our approach incorporates Constraint Extraction and the analysis of intensity preferences, features not addressed in their study.

International perspectives have played a pivotal role in advancing comparative analysis. Wang et al. [17] analysis of Chinese online restaurant reviews led to the proposal of a hybrid model that assigns weights to comparative relations based on preference intensity, providing nuanced insights into customer preferences. In a subsequent development, Liu, Wang, and Shao [18] adopted a BERT-CRF model for quintuple extraction from Chinese reviews. Their innovative integration of Comparative Sentences Recognition (CSR) and Comparative Element Extraction (CEE) tasks significantly enhanced the performance of CEE, indicating the potential of combining advanced language models with sequence labeling techniques. Compared to these methodologies, our SCRQE model, built upon a deep learning pipeline, not only focuses on extracting quintuple elements but also integrates constraint recognition and intensity analysis of preferences, aspects not covered in the models by Wang et al. [17] and Liu, Wang, and Shao [18]. Unlike their models, which primarily rely on conventional language models and sequence labeling, ours leverages a fine-tuned RoBERTa framework. This enhancement allows for a better understanding of nuanced comparative dynamics within questions, enabling a more granular analysis of comparative context and preferences.

Focused Comparative Preference Classification (CPC) Research has witnessed significant advancements through varied methodologies. Younis et al. [19] conducted multi-class CPC using a suite of machine learning classifiers across four datasets, with Random Forest outperforming others. Table 2 illustrates this with sample sentences and their corresponding preference labels from their study. In contrast to this method, our model employs an integration of NLP techniques and a refined comparative analysis algorithm. Specifically, our model harnesses the capabilities of the transformer-based RoBERTa model, which has been fine-tuned to identify and classify a wide range of comparative preferences directly from textual inputs. This enables our system to dynamically adapt to various comparative contexts and preferences without the need for manual entity identification or dependency on specific datasets. Our approach not only reduces the manual labor typically required in comparative preference classification but also enhances the accuracy and scalability of the process. By automating the extraction and classification of comparative elements, our model achieves a higher level of precision in detecting nuanced comparative relations and preferences across diverse questions and contexts.

Complementing this, Panchenko et al. [6] tackled the CPC task by introducing the CompSent-19 dataset and employing an XGBoost model augmented with InferSent embedding, as exemplified in Table 3, which includes sample sentences and preference labels. Compared to this, our SCRQE model utilizes a multi-layered approach that leverages the RoBERTa transformer model to enhance the depth and accuracy of preference classification. By integrating the advanced capabilities of RoBERTa with a comprehensive sentence-pair classification framework, our SCRQE model goes beyond traditional feature extraction and machine learning techniques. This allows for a more refined analysis of the subtleties in comparative sentences, significantly improving the recognition of comparative preferences across complex sentence structures and varied linguistic expressions. Additionally, our model's application of deep learning extends the potential for understanding and classifying nuanced preferences within a broader array of comparative contexts, setting a new benchmark for precision and adaptability in Comparative Preference Classification.

In comparison to the CompSent-19 dataset, our dataset offers extensive enhancements by supporting a more comprehensive scope of comparative questions. Unlike CompSent-19, which is limited to Comparative Preference Classification (CPC) without involving aspects, typically featuring only two product entities per sentence and not supporting multiple preferences in a single sentence, our dataset is meticulously curated to encompass a wider variety of comparative dynamics. It includes detailed annotations on preference intensity and constraints, which are crucial for a deeper and more nuanced understanding of comparative preferences. Additionally, our dataset facilitates the analysis of multiple comparative relations within a single question and includes aspects alongside the entities involved in comparisons. This allows for a richer exploration of comparative scenarios, significantly enhancing the granularity and accuracy of our comparative preference classifications. Moreover, by incorporating diverse linguistic patterns and supporting model training and evaluations across different languages and domains, our dataset not only complements but significantly extends existing resources, broadening the scope and depth of comparative analysis within natural language processing.

Further, Ma et al. [20] advanced the field with the Entity-aware Dependency-based Deep Graph Attention Network classifier, leveraging the same CompSent-19 dataset [6] to achieve state-of-the-art results. Notably, while these studies necessitated manual identification of compared entities, this research circumvents that constraint by proposing an automated deep learning-based CPC model. In comparison, our model automates the identification and classification process entirely, utilizing an advanced NLP framework that integrates the RoBERTa transformer architecture for real-time entity and preference detection. This integration significantly reduces the manual effort and improves the scalability and precision of the comparative analyses, offering a more dynamic and adaptable solution for handling complex comparative data across various domains.

////////////////////////////////////////////////////////////////////////////////////////////////////////////

The decisions for specific annotations and labels are in some cases unclear
even with the provided definitions and examples. For example, why
&quot;reasonable&quot; (line 275) is Non-Gradable? Is it different from
&quot;good/better&quot;? The definitions of preference types (lines 532-580) are
insufficiently explained. What is &quot;a comparison without a clear reference
point&quot; (XOR) or &quot;a degree of uncertainty&quot; (X)? For example, what are the
differences in meaning between the following four different annotations
from the text:

Is it better or not to upgrade A to B? (X)
What is the satisfaction level of A compared to B? (X-Strong Better)
Which phone is better overall performance, A or B? (XOR-Better)
Does A have better quality than B? (Better)

Dear Reviewer,

Thank you for your insightful feedback concerning the clarity of specific annotations and labels in our manuscript. Your comments have prompted us to conduct a thorough review and subsequent revision of our classification system for comparative preferences, particularly addressing the definitions and examples provided for each category.

We have refined our manuscript to more clearly articulate the nuances and distinctions between different types of comparative questions. This revision includes a more detailed explanation of each category, ensuring that the logic behind our annotations is transparent and comprehensible.

Here's how we've addressed your concerns:

- **Clarified Definitions**: We have expanded the definitions of our preference categories, particularly focusing on differentiating between 'Non-Gradable', 'XOR-type', and 'X-type' preferences. For example:
- **Non-Gradable questions** are now defined as those that do not imply a hierarchical ranking or explicit comparative roles, focusing instead on exploring unique characteristics or categorical differences without asserting superiority.
- **XOR-type questions** are clarified as those involving a clear preference direction (e.g., better or worse) but where the roles of subject and object are not fixed, allowing for a fluid interpretation of entity roles within the context of the comparison.
- **X-type questions** are those where the direction of preference remains undefined, although the roles of the entities (subject/object) are clearly identified, often involving hypothetical scenarios or conditions that explore potential rather than definitive outcomes.
- **Enhanced Examples:** We have included additional examples and expanded the context for each category to illustrate how these types of questions operate within our framework. This is intended to provide a clearer understanding of how different types of comparative questions are categorized and analyzed in our Comparative Preference Classification (CPC) task.

**Direct Responses to Your Queries:**

- **Reasonable vs. Good/Better:** In our initial manuscript, we classified the question "Which series of Samsung smartphones has a reasonable price, A series or S series?" as Non-Gradable. We understand that using 'reasonable' might have led to confusion because it does not directly compare entities on a hierarchical scale as 'good' or 'better' does. To clarify, 'reasonable' implies a qualitative assessment that does not inherently rank one entity over another, which aligns with the characteristics of a Non-Gradable comparison. However, acknowledging the potential for ambiguity, we have removed this example to ensure clarity in our comparative analysis framework. As a replacement, we will include the question "How does the user experience vary between the OnePlus 8T and the Google Pixel 5?" This example more clearly represents a Non-Gradable query as it explores distinctiveness without implying a hierarchy or direct comparison between the models, thus eliminating any confusion regarding the comparative context. This adjustment will enhance the accuracy of our manuscript in demonstrating Non-Gradable comparisons.
- **Comparative Directions in X and XOR-types:** We elucidate that 'X-type' questions handle situations with ambiguous directional preferences while the compared entities' roles are clear. Conversely, 'XOR-type' questions have a specified direction of preference (e.g., better, worse) but maintain fluidity and interchangeability in the compared entities' roles, allowing for a flexible assessment of the entities within the comparative context.

**Revision of Preference** **Definitions Section: The revised section, now titled "Defining Question Preference Types in Subjective Comparative Analysis,"** delves into how the clarity of preference direction and the definition of subject and object roles are assessed, enhancing the robust framework for analyzing comparative questions. We ensure that the distinctions between complete and incomplete preference types, such as Basic Preferences and more complex X-types or XOR-types, are clearly laid out. This includes specifying that in Basic and Intensity Preference types both the preference direction and the roles of entities are identified, while in X-types and XOR-types, one of these elements is typically left ambiguous.

We believe these revisions and enhancements address your concerns and significantly improve the clarity and scholarly rigor of our manuscript. We are grateful for the opportunity to refine our work based on your valuable insights and look forward to any further suggestions you may have.

We appreciate your feedback and hope that the revised manuscript meets your expectations and those of the scholarly community.

**To honor the reviewer's input and ensure the thoroughness of our response, we also include this addition here in our response letter:**

**Revised Section: Defining Question Preference Types in Subjective Comparative Analysis**

In this section, we delve into the nuanced classification of question types that our Comparative Preference Classification (CPC) framework can distinguish. Our approach categorizes questions based on the clarity of preference direction and the definition of subject and object roles, providing a robust framework for analyzing the subtleties of comparative questions.

- **Basic Preferences (Better/Worse/Equal):** This category includes straightforward queries where the comparative preference between two entities is explicitly stated and directly inferable from the question. These are the foundational comparative categories used to evaluate clear, direct comparisons without any implied complexity or subtlety. In these types of questions, both the direction of preference (e.g., <, >, =) and the roles of the compared entities as subject or object are clearly identified. To illustrate this point, consider the following examples:
  - "*Does the iPhone 12 have better quality than the iPhone 9*?" (labeled as 'Better') – Clearly identifies the iPhone 12 as the subject and the iPhone 9 as the object with a preference direction of 'better'.
  - "*Does the iPhone 8 have worse quality than the iPhone 9?*" (labeled as 'Worse') – Identifies the iPhone 8 as the subject and the iPhone 9 as the object with a preference direction of 'worse'.
  - "*Is the build quality of the S22 Ultra and the iPhone 14 Pro Max the same?*" (labeled as 'Equal') – Equates the two entities without assigning superiority, indicating an equal preference.
- **Preference Intensity (Strong Better/Strong Worse):** This category is used when the question emphasizes the degree or intensity of the comparison, going beyond simple better or worse to indicate a significant difference in quality or capability. These labels help distinguish questions that convey a stronger, more decisive comparative judgment, reflecting a deeper level of analysis in the user's query. Like Basic Preferences, these questions also clearly identify both the direction of preference and the roles of subject/object entities. Here are a few cases to clarify the concept:
  - "*Why is Huawei's 5G technology much better compared to Nokia's?*" (labeled as 'Strong Better') – This indicates a strong preference for Huawei's technology over Nokia's, with Huawei clearly positioned as the subject and Nokia as the object.
  - "*Is the appearance of the iPhone 10 significantly inferior to the iPhone 13?*" (labeled as 'Strong Worse”') – This suggests a strong negative preference for the iPhone 10's appearance in comparison to the iPhone 13, identifying the iPhone 10 as the subject and the iPhone 13 as the object."
- **XOR-type Preferences:** This category is designed for questions where the comparative direction (e.g., better, worse) is explicitly stated, yet the roles of the subject and object entities are left ambiguous or interchangeable. XOR-type questions typically involve a direct comparison where the entities are not fixed in specific roles, thereby allowing for a fluid and context-dependent interpretation of which entity is preferred. This flexibility in entity roles, paired with a clear comparative preference, characterizes XOR-type preferences as particularly adept at handling comparisons that require a flexible assessment of entity positions within the comparative context. Below are some illustrative examples:
  - "*Which phone has better overall performance, Xiaomi Mi Max or Lenovo Phab 2 plus?*" (XOR-Better) – This query clearly states that one phone performs better overall but leaves open which phone is the subject and which is the object, encouraging an evaluative comparison without fixed roles.
  - "*Samsung Galaxy A80 or iPhone 10, which one has lower quality?*" (XOR-Worse) – While it is clear that one phone is considered to have lower quality, the query does not assign specific roles to the devices, reflecting an open-ended assessment where either phone could be the subject or the object depending on the context.
- **X-type Preferences:** This category is applied to questions where the comparative direction of preference (e.g., better, worse) remains undefined or ambiguous, although the roles of the subject and object entities are clearly identified. These preferences often capture scenarios that involve hypothetical or conditional comparisons, where the actual comparative outcome is left open or undecided. Such questions typically explore potential comparisons rather than confirming definitive attributes, making the assessment more about weighing possibilities and less about establishing concrete judgments. This category addresses the inherent uncertainty in comparative analysis by allowing for a nuanced exploration of comparative relations without a predetermined preference direction. To elucidate, here are several examples:
  - "*How is the quality of the iPhone 11 compared to the Samsung Galaxy Note 10 Plus?*" (X) – This question invites an analysis of quality without specifying which phone is superior, keeping the comparative judgment open-ended and focusing on exploring the potential differences rather than concluding definitively.
  - "*How much better was the battery of the iPhone 13 mini compared to the iPhone 12 mini?*" (X-Strong Better) – This inquiry explores the potential improvement in battery performance between two models, without explicitly stating that one is definitively better, thus leaving room for interpretation based on user experience or other factors.
- **Non-Gradable Preferences:** This category encompasses comparative questions where the language used does not suggest a hierarchical ranking or scale of preference and does not specify the roles of subject or object entities in a traditional comparative context. These preferences are key for identifying comparisons that emphasize unique or distinguishing features without implying superiority, inferiority, or explicit comparative roles. In Non-Gradable questions, both the direction of preference (e.g., less than, greater than) and the roles of subject/object entities are typically undefined. This setup facilitates discussions focused on exploring unique characteristics or differences that do not categorically rank one entity above another. Such an approach is invaluable for highlighting distinctiveness or categorical differences rather than evaluating entities based on a conventional metric of comparison. Here are some examples to solidify the concept:
  - "*Is the Realme 8i in competition with Samsung A50?*" – This question does not establish a preference for either phone nor does it designate one as the subject or the other as the object, focusing instead on their market relationship.
  - "*Is the quality of the iPhone X different from that of Samsung Y?*" – Queries such as this seek to identify qualitative differences without suggesting that one is better or worse, or defining a comparative role for the devices.
  - "*Which iPhone series appearance looks distinguishable from the others?*" – This question is aimed at identifying which series is uniquely different in appearance, without ranking them or assigning roles of comparison.
  - "*What is the replacement phone for my Xiaomi Redmi Note 10 Lite among Oppo A54 and Vivo Y20? It must cost less than $1000.*" – This seeks a replacement based on a specific criterion (price) rather than comparing performance or quality, and does not set a comparative preference or define the roles of the phones involved.

In the classification of question types for our CPC task, we distinctly outline how the comparative questions are categorized based on the clarity of preference direction and the roles of the entities involved. Basic preference types, such as Better, Worse, Equal, Strong Better, and Strong Worse, clearly define both the direction of preference (e.g., <, >) and the roles of the subject and object entities, allowing for straightforward and explicit comparative analyses. However, the more complex X-types, XOR-types, and Non-Gradable categories, which we classify as incomplete preference types, either leave the direction of preference or the roles of the entities ambiguous or undefined. In these categories, one or more of these elements—either the direction of preference or the roles of the entities—are typically left ambiguous or undefined. Non-Gradable questions particularly do not imply any hierarchical comparison or clearly defined roles, focusing instead on highlighting distinctions without comparative rankings. This deliberate categorization accommodates the inherent variability and subtlety in subjective comparative questions, providing a flexible yet structured approach to understanding and classifying the nuances of user inquiries within our CPC framework.

////////////////////////////////////////////////////////////////////////////////////////////////////////////

What are the inter-annotator agreement values for these fuzzy categories?

Dear Reviewer,

Thank you for your insightful comments and for highlighting the importance of detailing the Inter-Annotator Agreement (IAA) values for the fuzzy categories within our Comparative Preference Classification (CPC) analysis. We recognize the significance of this aspect in demonstrating the robustness and reliability of our annotation process, especially given the subjective nature of these categories.

In this regard, **we have added a detailed section titled "Methodology for Assessing Annotator Agreement on Comparative Preferences"** to our manuscript. This section elaborates on our systematic approach to quantifying inter-annotator agreement, particularly focusing on the nuanced and subjective categories within the Comparative Preference Classification (CPC) analysis. Utilizing the Weighted Kappa statistic, grounded in a meticulously designed weighted disagreement matrix, we aim to offer a refined understanding of annotator consensus. This matrix accounts for the ordinal nature of the data and the variable degrees of disagreement across different comparative preferences, enabling a more accurate and nuanced measure of agreement. Through this addition, we demonstrate the rigor of our annotation process and the reliability of the resulting data, especially in handling the subjective complexities inherent in the CPC task.

**To honor the reviewer's input and ensure the thoroughness of our response, we also include this addition here in our response letter:**

**Revised Section: Annotation Process and Inter-Annotator Agreement Analysis**

This study leveraged the expertise of three annotators with backgrounds in computational linguistics and specializations in product domain sentiment analysis. Prior to annotation, the team underwent a comprehensive training session focused on the intricacies of subjective comparative questions. The training included an extensive review of key concepts and definitions relevant to comparative relations, such as entities, aspects, constraints, and comparative preferences. This ensured a shared understanding and application of the annotation guidelines. The complexity of identifying subject entities, object entities, and compared aspects within multiple comparative relations was emphasized due to their critical role in the task.

The annotation process was designed to be iterative, allowing annotators to independently label a shared set of questions before convening to discuss discrepancies and refine their interpretations of the guidelines. This collaborative approach facilitated a dynamic refinement of annotation practices, enhancing consistency across the dataset.

To quantify the level of agreement among the annotators, we employed a multifaceted approach, utilizing several inter-annotator agreement (IAA) metrics tailored to the specific aspects of the annotation task:

- **Fleiss' Kappa [24]:** This metric was chosen to assess the agreement on the identification of entities and aspects, which are foundational to constructing the comparative relations. Given its suitability for categorical data and its accommodation of multiple raters, Fleiss' Kappa provided a robust measure of consensus for these critical components.
- **Weighted Kappa [25]:** To account for the ordinal nature of comparative preferences, encompassing categories like "Better" to "XOR-Strong Worse," Weighted Kappa was employed. This metric assigns different weights to disagreements based on the severity within preference categories, offering a more nuanced assessment of annotator consistency in this intricate judgment domain.
- **Alpha U [26]:** To evaluate the agreement on extracting entire comparative relations, including Subject Entity, Object Entity, Compared Aspect, Constraint, and Comparative Preference, Alpha U was chosen. This metric is an adaptation of Krippendorff's Alpha [27] specifically designed for tasks involving segmenting and classifying text. By employing Alpha U, the study accurately measured annotator agreement on both the identification and contextual classification of these relations.

High inter-annotator agreement is essential for validating the reliability of the annotated dataset, especially in the complex domain of subjective comparative questions. This section summarizes the agreement metrics, reflecting the effectiveness of the annotation process across various components.

- **Entity and Aspect Identification:** The annotation process yielded a Fleiss' Kappa score of 0.94 for entity identification and 0.89 for aspect identification. These scores, indicating almost perfect agreement, attest to the effectiveness of our annotation training and guidelines in achieving a high degree of consistency among annotators for these fundamental components.
- **Constraint Recognition:** The Fleiss' Kappa score for constraint identification was 0.78, signifying substantial agreement. The slightly lower score compared to entity and aspect identification reflects the inherent challenges in recognizing and categorizing constraints, which often require a deeper inference of the question context.
- **Subject Entity, Object Entity, and Compared Aspect Identification:** The IAA metrics underscore the consistency in annotator judgments across key components, with Fleiss' Kappa scores revealing: Subject Entities at 95%, indicating almost perfect agreement; Object Entities at 92%, demonstrating substantial consensus; and Compared Aspects at 89%, reflecting significant agreement. These scores collectively highlight the effectiveness of our annotator training and guidelines in ensuring high-quality, consistent annotations for the foundational elements of comparative relation extraction.
- **Comparative Preference Classification:** The application of Weighted Kappa resulted in a score of 0.76 for comparative preferences. This substantial agreement level highlights the annotators' ability to navigate the intricate spectrum of preferences, supported by the detailed categorization scheme developed for this task.
- **Comparative Relation Extraction:** The comprehensive approach to annotating complete comparative relations was reflected in an Alpha U score of 0.80. This high level of agreement among annotators underscores the effectiveness of our iterative annotation process and collaborative discussions in clarifying complex relations, including the accurate identification of Subject Entities, Object Entities, and Compared Aspects.

The inter-annotator agreement analysis revealed a high level of consensus across all components of the annotation task, validating the reliability of the annotated dataset for extracting subjective comparative relations. While the process identified areas for improvement, particularly in constraint recognition, the overall results affirm the robustness of the annotation methodology. The collaborative and iterative nature of the annotation process, along with the targeted use of multiple IAA metrics, has established a strong foundation for subsequent analyses and model development based on this dataset.

**Addressing Disagreements among Annotators**

Despite the overall high levels of agreement, instances of disagreement among annotators served as valuable opportunities for further refining our annotation process and guidelines. Disagreements primarily arose in two areas: constraint recognition and the classification of nuanced comparative preferences.

To address these disagreements, we implemented a multifaceted resolution strategy, which included:

- **Guideline Refinement:** Disagreement analysis identified areas for improved clarity within the annotation guidelines. For constraint recognition, additional examples of explicit constraints were incorporated to ensure consistent identification. Similarly, comparative preferences received more detailed definitions and examples, particularly for complex categories like "X-types," "XOR-types," and "Non-Gradable." These refinements aim to achieve a shared understanding among annotators.
- **Focused Annotator Training:** Following guideline refinement, additional training sessions were held, focusing specifically on areas where disagreements were most prevalent. These sessions included interactive discussions on challenging examples, allowing annotators to explore and reconcile different interpretations of the guidelines.
- **Consensus Meetings:** For questions that continued to pose challenges after the initial annotation and review, consensus meetings were convened. During these meetings, annotators discussed their rationales for their classifications, with a senior member of the research team facilitating a consensus decision. This collaborative approach not only resolved individual instances of disagreement but also contributed to the continuous improvement of the annotation process.

These resolution strategies led to a marked decrease in the rate of disagreement in subsequent annotation rounds. Specifically:

- **Constraint Recognition:** The refinement of guidelines and focused training resulted in an increase in the Fleiss' Kappa score for constraint recognition from 0.78 to 0.82, indicating enhanced agreement and a more consistent application of the guidelines.
- **Comparative Preference Classification:** The consensus meetings and enriched annotation examples for comparative preferences improved the Weighted Kappa score from 0.76 to 0.81, reflecting a higher level of annotator consistency in this complex judgment area.
- **Holistic Enhancement across Annotations:** The improvements in Comparative Preference Classification (CPC) and Constraint Extraction, coupled with the meticulous clarification of Subject Entities, Object Entities, and Compared Aspects, collectively led to an overarching enhancement in SCRQD dataset's annotation quality. The observed improvement in the Alpha U score from 0.80 to 0.84 highlights the significant impact of the implemented resolution strategies. These strategies have facilitated the more accurate, reliable, and comprehensive extraction of comparative relations.

The iterative process of addressing annotator disagreements through guideline refinement, focused training, and consensus discussions proved instrumental in enhancing the reliability of our annotated dataset. Not only did this approach resolve specific instances of disagreement, but it also fostered a deeper collective understanding among the annotators, contributing to the overall quality and coherence of the dataset. These efforts underscore the dynamic nature of the annotation process and highlight the importance of continuous engagement and adaptation in achieving high-quality annotations for complex NLP tasks.

**Methodology for Assessing Annotator Agreement on Comparative Preferences**

To quantify the inter-annotator agreement on the Comparative Preference Classification (CPC) within the SCRQD dataset, we employed the Weighted Kappa statistic. This statistical method is particularly effective due to its ability to account for the ordinal nature of the data and the varying degrees of disagreement between different types of comparative preferences.

**Table 12.** Construction of the Weighted Disagreement Matrix

|  | **SW** | **W** | **E** | **B** | **SB** | **XOR-SW** | **XOR-W** | **XOR-E** | **XOR-B** | **XOR-SB** | **X-SW** | **X** | **X-SB** | **NG** |
| --- | --- | --- | --- | --- | --- | --- | --- | --- | --- | --- | --- | --- | --- | --- |
| **SW** | 0 | 1 | 5 | 5 | 5 | 1 | 2 | 5 | 5 | 5 | 2 | 5 | 5 | 5 |
| **W** | 1 | 0 | 5 | 5 | 5 | 2 | 1 | 5 | 5 | 5 | 2 | 5 | 5 | 5 |
| **E** | 5 | 5 | 0 | 5 | 5 | 5 | 5 | 1 | 5 | 5 | 5 | 5 | 5 | 5 |
| **B** | 5 | 5 | 5 | 0 | 1 | 5 | 5 | 5 | 1 | 2 | 5 | 5 | 2 | 5 |
| **SB** | 5 | 5 | 5 | 1 | 0 | 5 | 5 | 5 | 2 | 1 | 5 | 5 | 2 | 5 |
| **XOR-SW** | 1 | 2 | 5 | 5 | 5 | 0 | 1 | 5 | 5 | 5 | 2 | 5 | 5 | 5 |
| **XOR-W** | 2 | 1 | 5 | 5 | 5 | 1 | 0 | 5 | 5 | 5 | 3 | 5 | 5 | 5 |
| **XOR-E** | 5 | 5 | 1 | 5 | 5 | 5 | 5 | 0 | 5 | 5 | 5 | 5 | 5 | 5 |
| **XOR-B** | 5 | 5 | 5 | 1 | 2 | 5 | 5 | 5 | 0 | 1 | 5 | 5 | 3 | 5 |
| **XOR-SB** | 5 | 5 | 5 | 2 | 1 | 5 | 5 | 5 | 1 | 0 | 5 | 5 | 2 | 5 |
| **X-SW** | 1 | 2 | 5 | 5 | 5 | 2 | 3 | 5 | 5 | 5 | 0 | 5 | 5 | 5 |
| **X** | 5 | 5 | 5 | 5 | 5 | 5 | 5 | 5 | 5 | 5 | 5 | 0 | 5 | 5 |
| **X-SB** | 5 | 5 | 5 | 2 | 1 | 5 | 5 | 5 | 3 | 2 | 5 | 5 | 0 | 5 |
| **NG** | 5 | 5 | 5 | 5 | 5 | 5 | 5 | 5 | 5 | 5 | 5 | 5 | 5 | 0 |

The matrix lists various comparative preference categories such as SW (Strong Worse), W (Worse), E (Equal), B (Better), SB (Strong Better), and several XOR and X modified categories along with NG (Non-Gradable). Each cell in the matrix shows a weight from 0 to 5, indicating the level of disagreement between pairs of categories:

- **0** indicates no disagreement (same category).
- **1-2** indicates slight to moderate disagreement.
- **3-4** indicates significant disagreement.
- **5** indicates maximum disagreement (completely opposite or unrelated categories).

Below are detailed analyses of the disagreements and alignments within the preference categories as outlined in the Matrix:

- **Direct Comparisons (SW, W, B, SB):** Comparisons within this group (e.g., SW vs. W, B vs. SB) show low to moderate disagreements (1-2) except when compared with their direct opposites (e.g., SW vs. B, W vs. SB) where the disagreement is maximum (5). This reflects the clear conceptual differences in the direction of preference.
- **Neutral Category (E):** E (Equal) consistently has high disagreement (5) with all directional preferences (SW, W, B, SB) indicating its distinct nature as a neutral category where no comparative preference is implied.
- **XOR Categories:** These categories (XOR-SW, XOR-W, XOR-B, XOR-SB) typically show moderate disagreements with their non-XOR counterparts (1-2) and high disagreements (5) with categories that have opposite directions. The slight disagreements between similar XOR and non-XOR categories suggest that XOR adds a layer of conditionality but does not entirely change the fundamental comparative nature.
- **X Categories:** X and X-SW, X-SB show a consistent pattern of maximum disagreement (5) with all other categories except when compared to themselves (0) or similar types (low to moderate). This indicates the complex nature of these categories in comparative contexts.
- **Non-Gradable (NG):** NG shows maximum disagreement (5) with all other categories, reflecting its role in capturing comparative questions that do not imply any hierarchical or qualitative comparison.

This matrix serves as the foundation for calculating the Weighted Kappa statistic, facilitating a nuanced analysis of annotator consistency that goes beyond mere agreement, to also consider the relative impact of different types of disagreements on the overall reliability of the data.

By applying the Weighted Kappa statistic grounded in this comprehensive weighted disagreement matrix, we offer a sophisticated measure of agreement that aligns with the complex, multi-dimensional nature of comparative preferences in subjective questions. This methodology not only highlights the high degree of consensus among annotators in identifying and classifying a wide array of preferences but also underscores the robustness of SCRQD dataset for subsequent analyses of comparative relations.

////////////////////////////////////////////////////////////////////////////////////////////////////////////

From the stylistic point of view, the text is flooded with too many
acronyms, making the comprehension and navigation in some parts quite
difficult.

Dear Reviewer,

Thank you for your insightful feedback concerning the extensive use of acronyms in our manuscript. We acknowledge that the overuse of acronyms might have hindered the text's readability and made navigating through the content challenging.

In response to your comments, we have thoroughly revised the manuscript to enhance clarity and ease of comprehension. We have eliminated the acronyms B, SB, E, W, SW, XOR-SB, XOR-B, XOR-E, XOR-W, XOR-SW, X-SB, X, X-SW, and Non-Grad, and now use their full expressions: Better, Strong Better, Worse, Strong Worse, Equal, XOR-Better, XOR-Strong Better, XOR-Equal, XOR-Worse, XOR-Strong Worse, X, X-Strong Better, X-Strong Worse, and Non-Gradable throughout the text. This change ensures that each term is immediately clear to all readers without needing to recall the meaning of each acronym.

Furthermore, to simplify and clarify the presentation of our comparative framework, we have replaced the acronyms SE, OE, CA, CO, and CP with their full terms Subject Entity, Object Entity, Compared Aspect, Constrained Phrase, and Comparative Preference, respectively. This modification not only aids in better understanding but also reduces the cognitive load on our readers, making the manuscript more accessible.

However, we have retained the acronyms for the three distinct tasks of our model: EE (Element Extraction), CEI (Compared Elements Identification), and CPC (Comparative Preference Classification). We have also kept the acronyms SCRE (Subjective Comparative Relation Extraction) for our model, SCRQE (Subjective Comparative Relation Quintuple Extraction) for the model name, and SCRQD (Subjective Comparative Relation Quintuple Dataset) for our dataset. These acronyms are essential to maintain consistency and avoid redundancy in the detailed technical discussions within our work.

We believe these adjustments will substantially improve the manuscript's readability and navigability, allowing readers to engage more effectively with the content without the interruption of frequent acronym decoding.

We are grateful for your constructive criticism, which has significantly contributed to enhancing the presentation and accessibility of our research. Thank you for helping us improve our manuscript.

////////////////////////////////////////////////////////////////////////////////////////////////////////////

Another example of &quot;exaggeration&quot; can be seen in the emphasis on the
Multi-Task Learning (MTL) approach used in the system where the actual
difference is just between multiple binary classifiers (here single task
approaches) and one multi-class classifier. Multi-class token
classification is the most standard way of named entity recognition which
exactly corresponds to the underlying task of the presented system. Here
using three separate single-task or binary classifiers would be more
unusual.

Dear Reviewer,

Thank you for your insightful observations regarding our use of the Multi-Task Learning (MTL) approach within the Element Extraction (EE) phase of our research, particularly focusing on the nuanced application of multiple binary classifiers versus our adoption of a unified multi-class token classification strategy through MTL in the context of named entity recognition tasks.

The choice of MTL over separate single-task classifiers was driven by the complex interdependencies among Entity, Aspect, and Constraint extraction tasks within our dataset. This approach was predicated on the hypothesis that a unified model could better leverage shared features across tasks, thereby enhancing performance and reducing the risk of overfitting, as evidenced by literature in the field (Worsham and Kalita, [28]).

This complexity is further exemplified in our dataset by quesions where a single token may fulfill multiple roles. For instance, in the query "What are the best smartphones with a built-in stylus feature with a good quality display and RAM, other than Samsung?", the term "Samsung" serves as both an Entity and a Constraint, illustrating its dual function in the query. Similarly, "built-in stylus feature" spans both Aspect and Constraint categories, highlighting the intricate nature of classification within our dataset.

Moreover, in our case, the inclusion of constraints necessitates a framework that allows for the nuanced classification of tokens, which precludes assigning a unique label to each token. Instead, we require a more sophisticated label scheme that encompasses the multi-dimensional roles tokens can play. This necessitates expanding beyond the traditional nine labels to adequately represent the complexity of our data. This delineation ensures each token's multi-faceted role is captured, underscoring the need for an MTL approach to effectively manage the classification challenge.

We appreciate your continued guidance and support in improving our manuscript. We eagerly await any additional feedback you may have.

////////////////////////////////////////////////////////////////////////////////////////////////////////////

The schema in Figure 2 of the model for the entity extraction task is
misleading, displaying three separate label outputs from the input
question. The correct form is a sequence-to-sequence process resulting in
token labels (one to one for each token in the question).

Dear Reviewer,

Thank you for pointing out the ambiguity in the depiction of our model's schema within Figure 2, particularly regarding the entity extraction task. Your observation is invaluable, highlighting a discrepancy between the illustrative representation and the actual operational mechanics of our approach, which indeed aligns with the sequence-to-sequence process you've accurately described.

In response to your feedback, we recognize the necessity to rectify this misrepresentation. The intent of Figure 2 was to convey the Multi-Task Learning (MTL) framework's capacity to consider the nuanced roles that tokens play within the context of Entity, Aspect, and Constraint extraction tasks. However, we understand that the figure inadequately communicated the sequence-to-sequence nature of the token labeling process, wherein each token in the question is assigned a corresponding label, reflecting a one-to-one mapping essential for accurate entity extraction.

To address this, **we revised Figure 2 to more accurately illustrate the sequence-to-sequence labeling process.** This correction ensured the figure aligns with the described methodology, where the model processes input tokens and outputs a label for each token, effectively capturing the intricate interdependencies and multi-dimensional roles highlighted in our dataset. We also provided additional clarification in the accompanying text to ensure there was no ambiguity in interpreting the model's operational framework.

We appreciate your keen eye and the opportunity to clarify this aspect of our work. This feedback is crucial for improving the clarity and accuracy of our manuscript, and we are committed to making the necessary revisions to better communicate our research approach.

Thank you again for your guidance and support in enhancing the quality of our manuscript. We look forward to your further feedback.

**To honor the reviewer's input and ensure the thoroughness of our response, we also include this addition here in our response letter:**

**Fig 2.** Proposed MTL model for EE task.

////////////////////////////////////////////////////////////////////////////////////////////////////////////

The description of the second phase is unnecessarily long (lines 364-500,
more than 5 pages). The text mostly repeats the same (relatively simple)
procedure several times. For example, Tables 8 and (later) 11 are not
justified as the other 3 methods are not used at all here. Algorithm 1 does
not offer anything new to the text. One page should be enough here.
What is left unexplained, is the distinction between Subject and Object
(Table 9) where in some sentences there are only multiple subjects and in
others subjects with objects without explanation.

Dear Reviewer,

Thank you for your invaluable feedback which has significantly contributed to refining the clarity and focus of our manuscript, particularly regarding the Compared Elements Identification (CEI) phase. Your constructive critique has been instrumental in guiding our efforts to enhance both the presentation and methodological transparency of our work.

In response to your insightful suggestions, we have made the following precise revisions to address the concerns raised:

- **Streamlined CEI Phase Description:** We've condensed the narrative around the CEI phase, effectively eliminating redundancies while accentuating the NLI-M technique's role in auxiliary sentence generation. This refinement has allowed us to present the sentence-pair classification task and the selection rationale for NLI-M with greater clarity and brevity, ensuring the focus remains on our method's novel contributions.
- **Focused Methodological Discussion:** In line with your observation, discussions surrounding the QA-M, QA-B, and NLI-B methods have been removed from the primary text. This change underscores our methodological focus on the NLI-M approach, which our analysis confirms as most appropriate for capturing the nuanced comparative preferences within our dataset.
- **Rearrangement of Supplementary Material:** Heeding your recommendation, **we relocated Algorithm 1 and Tables 10, 12, and 13 to appendix section,** providing a detailed guide to automatic pseudo-sentence generation. This adjustment aims to declutter the main text, ensuring a streamlined narrative flow. Furthermore, **we have excised Table 11,** recognizing it as superfluous given our focused application of NLI-M.
- **Clarified Entity Role Distinction:** We have now clearly delineated the roles of 'Subject' and 'Object' within comparative questions, addressing previously noted ambiguities. This added clarity elucidates the functional dynamics of subjects and objects within comparative frameworks, rectifying the confusion surrounding scenarios with multiple subjects or combined subject and object entities without explicit comparative relationships.

These targeted revisions seek not only to refine the description of the CEI phase but also to bolster the overall coherence and accessibility of our methodological exposition. Through these improvements, we aim to more effectively communicate the innovative facets of our research.

We deeply appreciate the opportunity to refine our manuscript in light of your feedback and remain eager for any further guidance. We are optimistic that these adjustments will markedly elevate the manuscript's contribution to the field.

**To honor the reviewer's input and ensure the thoroughness of our response, we also include this addition here in our response letter:**

**3.3.2. Compared Elements Identification (CEI)**

The CEI phase is a fundamental component of our analytical framework, employing a sentence-pair classification strategy to delineate the comparative relationships within each question. This meticulous process identifies Subject Entities, Object Entities, Compared Aspects, and Constraints, offering a comprehensive understanding of the comparative interactions present. This phase is indispensable due to the inherent complexities and ambiguities of subjective comparative questions, where entities may not consistently assume subject or object roles across different comparative relations within the same question.

**3.2.2.1. Entity Role Identification (ERI)**

At the core of the CEI phase, the ERI process is instrumental in assigning roles to entities as Subject, Object, or None. This classification is crucial for accurately distinguishing between subject and object entities, which cannot be solely achieved through BIO labeling used in the first phase for the following reasons:

1) **Variability in Entity Roles:** Entities within a single question may not always serve as subjects or objects in the comparative relation, necessitating a dedicated mechanism for role identification.

2) **Multiple Comparative Relations:** Subjective comparative questions often involve multiple comparative relations within a single context, requiring a method to differentiate entity roles across these relations.

3**) Entities with No Subject/Object Role:** Some entities mentioned within a question do not serve as subjects or objects in any comparative relation, such as "Samsung" with respect to aspects like a built-in stylus feature, quality display, and RAM.

The ERI process leverages contextual cues and linguistic patterns specific to subjective comparative questions to ensure accurate role identification. This enhances the model’s ability to navigate the intricacies of comparative relations and deliver more accurate and nuanced results. This classification establishes a framework for mapping the structure of comparative relations within the dataset, encompassing:

- **Systematic Input Structuring for RoBERTa:** We methodically structure inputs for the RoBERTa model by concatenating each question with auxiliary sentences, interspersed with RoBERTa's designated separator tokens, denoted as "<S> + Q_1 + <\S> + auxiliary sentence + <\S>". This structuring ensures data clarity and context preservation, utilizing "<S>" and "</S>" to mark the beginning and separation within RoBERTa's operational framework.
- **Auxiliary Sentence Generation Technique:** Drawing inspiration from Sun et al.'s work [31], there are four auxiliary sentence generation methods for sentence-pair classification tasks: NLI-B (Natural Language Inference with Binary output), NLI-M (Natural Language Inference with Multiple outputs), QA-B (Question Answering with Binary output), and QA-M (Question Answering with Multiple outputs). Each method has its merits; however, NLI-M is particularly suited to our dataset's requirements, accommodating the varied comparative nuances, including 'Strong Better/Worse'. This approach is essential for thorough role identification, where every entity within the question is examined in conjunction with all mentioned aspects. This guideline aids in maintaining clarity and consistency in the classification of entities across a diverse range of comparative contexts. For a detailed overview of our auxiliary sentence generation algorithm, see Appendix A. The utilization of these auxiliary sentence generation techniques lays the foundation for a more nuanced and detailed subjective comparative analysis, leading us to two key strategies for handling the intricacies of our dataset's subjective comparative questions:
  - **Generalization of Aspects in Comparative Analysis:** In scenarios where questions do not specify aspects (as shown in Case 4 and 6 of Table 15), a generalized "features" label is applied. This approach facilitates a nuanced examination of comparative relations, ensuring the dataset's comprehensive representation.
  - **Classifying Comparative Entities Based on Implicit Preferences:** In our approach to classifying comparative entities within questions, we have established that when a relation in a question exhibits preferences beyond the conventional categories of better/worse or strong better/strong worse, such comparative entities are considered "subjects." This is demonstrated in cases 2, 3, and 6 of Table 15, where multiple entities are discussed within a comparative context without a predetermined direction of preference. We adopt this method to embrace a broader spectrum of comparative nuances, acknowledging that certain questions suggest a comparative evaluation toward these entities without explicitly indicating a preference direction. This includes scenarios involving X-types, XOR-types, Equal, and Non-Gradable situations. By adopting this strategy, we aim to accurately reflect the subtleties inherent in natural language expressions of comparison, thus ensuring a comprehensive analysis of the dataset's comparative relations.
- **Adaptation of RoBERTa for ERI:** We utilized a fine-tuned RoBERTa model for the ERI task for sentence-pair classification to discern comparative roles efficiently.
- **Identification of Compared Aspects through ERI:** The ERI process, while categorizing entities into Subject, Object, or None, implicitly facilitates the identification of Compared Aspects. Given that each auxiliary sentence generated for classification includes aspects tied to the entities, determining an entity's role as a Subject or Object inherently also identifies the aspects under comparison. This implicit determination of Compared Aspects is a direct outcome of the structured approach to generating auxiliary sentences, ensuring that each Subject or Object entity is contextually linked to its corresponding aspect within the question.

////////////////////////////////////////////////////////////////////////////////////////////////////////////

Is the second phase actually needed? What if the differentiation between
subject and object was expressed by the BIO labels in the first phase?

Dear reviewer,

Thank you for your insightful comment regarding the necessity of the second phase in our proposed pipeline. Your query touches upon a fundamental aspect of our approach, and we appreciate the opportunity to provide further clarification.

In response to your query, it's crucial to acknowledge the limitations of solely relying on BIO labeling for entity recognition and subject/object differentiation within the context of subjective comparative questions. Your points aptly highlight the inherent complexities and ambiguities present in such questions, where entities may not consistently assume subject or object roles across different comparative relations within the same question.

Considering the intricacies involved, the second phase, CEI, assumes a pivotal role in accurately identifying and distinguishing between subject and object entities. This differentiation cannot be solely achieved through BIO labeling in the first phase due to the following reasons:

1. **Variability in Entity Roles:** A single question may contain entities that do not necessarily serve as subjects or objects in the comparative relation. As seen in your example, entities like "Samsung Galaxy A11" and "iPhone 8" are mentioned but may not always assume subject or object roles.
2. **Multiple Comparative Relations:** Subjective comparative questions often involve multiple comparative relations within a single context. As a result, entities within the same question may participate as subjects in one relation and as objects in another, further complicating the task of subject/object differentiation.
3. **Entities with No Subject/Object Role:** In some cases, entities within a question may not have a subject or object role in the comparative relation. For example, "Samsung" with respect to aspects such as built-in stylus feature, quality display, and RAM, assumes no subject/object role.

Given these challenges, the CEI phase is indispensable as it provides a dedicated mechanism for identifying and disambiguating the roles of entities within the context of each comparative relation. By incorporating contextual cues and linguistic patterns specific to subjective comparative questions, this phase ensures the accurate identification of subject and object entities, thereby enhancing the overall performance and robustness of the model.

In conclusion, while BIO labeling in the first phase serves as a foundational step for entity recognition, the complexities of subjective comparative questions necessitate a separate phase, CEI, for effectively identifying subject and object entities. This approach enables our model to navigate the intricacies of comparative relations and deliver more accurate and nuanced results.

To further clarify this essential aspect of our methodology, **we have included a detailed explanation in Section 3.3.2, 'Compared Elements Identification (CEI),' and its subsection 3.3.2.1, 'Entity Role Identification (ERI),'** of our manuscript. This addition aims to highlight the critical role and underlying rationale of the CEI phase, reinforcing its significance within our analytical approach.

We hope this response effectively addresses your concern and underscores the importance of the CEI phase in our proposed pipeline.

Thank you once again for your constructive feedback, which has been instrumental in enhancing the clarity and comprehensiveness of our manuscript.

**To honor the reviewer's input and ensure the thoroughness of our response, we also include this addition here in our response letter:**

**Revised Section 3.3.2. Compared Elements Identification (CEI)**

The CEI phase is a fundamental component of our analytical framework, employing a sentence-pair classification strategy to delineate the comparative relationships within each question. This meticulous process identifies Subject Entities, Object Entities, Compared Aspects, and Constraints, offering a comprehensive understanding of the comparative interactions present. This phase is indispensable due to the inherent complexities and ambiguities of subjective comparative questions, where entities may not consistently assume subject or object roles across different comparative relations within the same question.

**Revised Section 3.2.2.1. Entity Role Identification (ERI)**

At the core of the CEI phase, the ERI process is instrumental in assigning roles to entities as Subject, Object, or None. This classification is crucial for accurately distinguishing between subject and object entities, which cannot be solely achieved through BIO labeling used in the first phase for the following reasons:

1) **Variability in Entity Roles:** Entities within a single question may not always serve as subjects or objects in the comparative relation, necessitating a dedicated mechanism for role identification.

2) **Multiple Comparative Relations:** Subjective comparative questions often involve multiple comparative relations within a single context, requiring a method to differentiate entity roles across these relations.

3**) Entities with No Subject/Object Role:** Some entities mentioned within a question do not serve as subjects or objects in any comparative relation, such as "Samsung" with respect to aspects like a built-in stylus feature, quality display, and RAM.

The ERI process leverages contextual cues and linguistic patterns specific to subjective comparative questions to ensure accurate role identification. This enhances the model’s ability to navigate the intricacies of comparative relations and deliver more accurate and nuanced results. This classification establishes a framework for mapping the structure of comparative relations within the dataset, encompassing:

- **Systematic Input Structuring for RoBERTa:** We methodically structure inputs for the RoBERTa model by concatenating each question with auxiliary sentences, interspersed with RoBERTa's designated separator tokens, denoted as "<S> + Q_1 + <\S> + auxiliary sentence + <\S>". This structuring ensures data clarity and context preservation, utilizing "<S>" and "</S>" to mark the beginning and separation within RoBERTa's operational framework.
- **Auxiliary Sentence Generation Technique:** Drawing inspiration from Sun et al.'s work [31], there are four auxiliary sentence generation methods for sentence-pair classification tasks: NLI-B (Natural Language Inference with Binary output), NLI-M (Natural Language Inference with Multiple outputs), QA-B (Question Answering with Binary output), and QA-M (Question Answering with Multiple outputs). Each method has its merits; however, NLI-M is particularly suited to our dataset's requirements, accommodating the varied comparative nuances, including 'Strong Better/Worse'. This approach is essential for thorough role identification, where every entity within the question is examined in conjunction with all mentioned aspects. This guideline aids in maintaining clarity and consistency in the classification of entities across a diverse range of comparative contexts. For a detailed overview of our auxiliary sentence generation algorithm, see Appendix A. The utilization of these auxiliary sentence generation techniques lays the foundation for a more nuanced and detailed subjective comparative analysis, leading us to two key strategies for handling the intricacies of our dataset's subjective comparative questions:
  - **Generalization of Aspects in Comparative Analysis:** In scenarios where questions do not specify aspects (as shown in Case 4 and 6 of Table 15), a generalized "features" label is applied. This approach facilitates a nuanced examination of comparative relations, ensuring the dataset's comprehensive representation.
  - **Classifying Comparative Entities Based on Implicit Preferences:** In our approach to classifying comparative entities within questions, we have established that when a relation in a question exhibits preferences beyond the conventional categories of better/worse or strong better/strong worse, such comparative entities are considered "subjects." This is demonstrated in cases 2, 3, and 6 of Table 15, where multiple entities are discussed within a comparative context without a predetermined direction of preference. We adopt this method to embrace a broader spectrum of comparative nuances, acknowledging that certain questions suggest a comparative evaluation toward these entities without explicitly indicating a preference direction. This includes scenarios involving X-types, XOR-types, Equal, and Non-Gradable situations. By adopting this strategy, we aim to accurately reflect the subtleties inherent in natural language expressions of comparison, thus ensuring a comprehensive analysis of the dataset's comparative relations.
- **Adaptation of RoBERTa for ERI:** We utilized a fine-tuned RoBERTa model for the ERI task for sentence-pair classification to discern comparative roles efficiently.
- **Identification of Compared Aspects through ERI:** The ERI process, while categorizing entities into Subject, Object, or None, implicitly facilitates the identification of Compared Aspects. Given that each auxiliary sentence generated for classification includes aspects tied to the entities, determining an entity's role as a Subject or Object inherently also identifies the aspects under comparison. This implicit determination of Compared Aspects is a direct outcome of the structured approach to generating auxiliary sentences, ensuring that each Subject or Object entity is contextually linked to its corresponding aspect within the question.

///////////////////////////////////////////////////////////////////////////////////////////////////////////

Dropout of 0.0 in Table 20 is slightly unusual. Moreover, this parameter is
not included in the list in 4.2. Hyper-Parameter Optimization with other
values.

Dear Reviewer,

Thank you for your insightful feedback regarding the optimization of the dropout rate within our model. Your comments prompted a thorough reassessment of our approach to hyperparameter tuning, which is crucial for model generalization and performance.

In response to your observations, we conducted detailed empirical testing to identify optimal dropout rates that enhance our model’s performance across the EE, CEI, and CPC tasks. This process involved systematically varying the dropout rate and assessing the model’s performance on a validation set to ensure an unbiased evaluation of its generalization capabilities. Through rigorous analysis, we determined that a dropout rate of 0.15 is optimal for the EE task. However, our findings also revealed that the impact of different dropout rates tested on the CEI and CPC tasks was negligible, leading us to maintain the current configuration for these tasks without additional dropout.

To further enhance our manuscript and address your valuable suggestions, we expanded the scope of our hyper-parameter optimization section. This update includes refining the dropout rate based on empirical evidence and exploring other critical hyper-parameters, such as the use of the AdamW optimizer. AdamW is known for its improved handling of weight decay, which often leads to better generalization, and its inclusion in our methodology has further enhanced our model’s performance.

It's important to note that these detailed empirical investigations and the resultant findings were primarily conducted in response to your valuable feedback and to elucidate our methodology. To maintain the focus and coherence of our revised manuscript, we will not include the exhaustive details of this hyperparameter optimization process. Instead, we will update the manuscript to reflect the final refined dropout values for the CEI and CPC tasks, as identified through our empirical testing. This approach ensures that our revised manuscript concisely presents the most relevant and impactful modifications to our model, directly addressing your concerns while preserving the clarity and succinctness of our presentation.

We believe that specifying the optimized dropout rates for the CEI and CPC tasks in the revised manuscript clearly demonstrates our commitment to refining our model based on empirical evidence and reviewer feedback. This refinement significantly contributes to the model’s robustness and its ability to generalize across diverse comparative question analyses.

We are immensely grateful for your feedback, which has been instrumental in enhancing the precision and effectiveness of our model. We look forward to your further guidance and insights.

**To honor the reviewer's input and ensure the thoroughness of our response, we also include this addition here in our response letter.**

**Revised Section 4.1: Hyper-Parameter Optimization**

We conducted thorough experiments to determine optimal hyper-parameters for each component of the model—Element Extraction (EE), Compared Elements Identification (CEI), and Comparative Preference Classification (CPC). The selected hyper-parameter configurations, detailed in Table 16, were consistently employed for the EE, CEI, and CPC models without further changes.

Table 16. Hyper-parameter setup for the proposed model across all tasks.

Parameters	EE	CEI	CPC

Batch Size	8	64	64

Max-Seq Length	100	100	100

Number of Epochs	5	5	5

Optimizer	AdamW	Adam	Adam

Learning Rate	5e-5	3e-5	3e-5

Dropout-Rate	0.15	0.0	0.0

////////////////////////////////////////////////////////////////////////////////////////////////////////////

Table 24 with a Comparison of Aspect Extraction models ignores some works
with better results, e.g.

Wu, Z., &amp; Ong, D. C. (2021). Context-guided bert for targeted
aspect-based sentiment analysis. In Proceedings of the AAAI conference
on artificial intelligence (Vol. 35, No. 16, pp. 14094-14102).

or even the work [24] from the text presenting BERT-pair which was,
however, not included in this comparison table.

Dear Reviewer,

Thank you for your attention to the comprehensiveness of Table 24, where we compare models related to aspect extraction. Your mention of the studies by Wu & Ong (2021) and the BERT-pair work highlighted in reference [24] raises an important point regarding the scope of our review.

Upon reviewing these studies, we acknowledge that both are indeed significant contributions to the field of aspect-based sentiment analysis. However, their primary focus is on aspect categorization, also known as Aspect Category Detection. This process involves assigning predefined categories to aspects that are either mentioned explicitly or implied in the text, which differs from the aspect extraction task that our table aims to outline. Aspect extraction, as defined in our study, specifically involves the identification and extraction of aspect terms from raw text data, a foundational step preceding categorization.

To clarify this distinction and ensure the focus remains aligned with the specific challenges of extraction, we opted not to include studies primarily centered on categorization tasks in Table 24.

Thank you for allowing us the opportunity to clarify these aspects of our study. We appreciate your guidance and look forward to your feedback.

////////////////////////////////////////////////////////////////////////////////////////////////////////////

The details of the comparison evaluation are missing. Did you run the
compared models yourself? What is e.g. the &quot;classification over
single-sentence on the CompSent-19 dataset&quot; compared in Figure 7? What is
the Different label in Figure 8?

Dear Reviewer,

Thank you for your insightful comments requesting further details on our comparative evaluation process. In light of your queries, we have refined our manuscript to better articulate the nuances of our approach, particularly in relation to the datasets employed for comparative analysis.

Our study uniquely addresses the challenge of comparing entities within question-based formats, a complexity not inherently captured by sentence-level datasets like CompSent-19 and Camera-COQE. Given this distinction, our dataset includes specialized classifications such as x-types and xor-types, which are tailored to the intricacies of question-based comparative analysis. These classifications, absent in the aforementioned datasets, underscore our endeavor to explore beyond the conventional binary distinctions of 'Better'/'Worse' typically found in sentence datasets. To align our analysis with these established benchmarks and facilitate a coherent comparison, we adapted our model to focus on the standard comparative categories these datasets support, notwithstanding our model's capability to navigate a broader spectrum of comparative nuances including 'Strong Better'/'Strong Worse'.

**We add a subsection "4.4.1. Alignment with CompSent-19 and Camera-COQE Datasets" in the revised section of our manuscript**. This addition elucidates the strategic adjustments made to align our methodology with the binary comparative frameworks of the CompSent-19 and Camera-COQE datasets:

- We adapted our evaluation framework to reflect the binary distinctions ('Better'/'Worse') prevalent in these datasets, ensuring a coherent comparison despite our annotations' more nuanced categories ('Strong Better'/'Strong Worse').
- We consciously excluded question-specific comparative types (x-types and xor-types) from our analysis when applying our model to these external datasets, highlighting our model's versatility in accommodating diverse analytical contexts.
- We provided clarity on the "Different" label as used in the Camera-COQE dataset, signifying instances where entities possess unique, non-comparable attributes, aligning with our classification of 'non-gradable' comparisons.

This comprehensive approach not only showcases our model’s adaptability but also emphasizes our commitment to advancing the field of comparative preference classification by offering a detailed exploration of complex comparative relations.

**To address your specific inquiries:**

- **Comparison Evaluation:** We independently conducted comparisons using our RoBERTa-pair-NLI-M model against established models referenced in the Camera-COQE and CompSent-19 datasets, ensuring a direct and fair assessment of our methodology's effectiveness.
- **Classification on CompSent-19:** The improvement in identifying comparative relations using our sentence-pair classification approach, as opposed to single-sentence classification, is quantitatively showcased in Figure 7.
- **"Different" Label in Figure 8:** This label is utilized to denote instances diverging from traditional binary comparisons, encapsulating entities with distinct attributes that defy direct comparison, a testament to our model's ability to interpret a wide array of comparative nuances.

We apologize for the oversight in not including these critical evaluation details in our initial submission. The revised manuscript now features these additions, aiming to offer a more thorough and transparent view of our comparative analysis and the model's classification capabilities.

We are grateful for the opportunity to refine our submission based on your valuable feedback and look forward to any further guidance you may offer.

**To honor the reviewer's input and ensure the thoroughness of our response, we also include this addition here in our response letter:**

**Revised Section 4.4.1. Alignment with CompSent-19 and Camera-COQE Datasets**

To demonstrate the adaptability and comprehensive nature of our model in comparative preference classification, it was necessary to align our methodology with the established benchmarks of the CompSent-19 and Camera-COQE datasets. This alignment involved several key adjustments:

- **Adapting to Binary Distinctions:** These datasets primarily focus on binary distinctions of 'Better'/'Worse', without accommodating the nuanced categories of 'Strong Better'/'Strong Worse' present in our annotations. Given this discrepancy, we adapted our model's evaluation framework to match the outputs with those datasets, ensuring a coherent comparison. This adjustment involved mapping our model's more granular preference categories to the broader 'Better' and 'Worse' classifications used in these datasets.
- **Excluding Question-Specific Comparative Types:** Our dataset uniquely incorporates X-types and XOR-types classifications, absent from standard sentence-level datasets. Recognizing this discrepancy, we tailored our comparative analysis to exclude these question-specific categories, focusing instead on the standard comparative types supported by external datasets. This adjustment ensures a balanced comparison and highlights our model’s flexibility in adapting to diverse analytical contexts. It also reflects our commitment to advancing comparative preference classification by accommodating a wide range of comparative inquiries, even when specific question-oriented types are set aside for consistency in cross-dataset evaluations.
- **Clarifying the "Different" Category:** The "Different" label in the Camera-COQE dataset [5] signifies instances where comparative analysis does not neatly align with the traditional 'Better', 'Worse', or 'Equal' comparisons. These are scenarios in which entities possess distinct qualities that make them incomparable on a linear scale of superiority or inferiority. This category was introduced to recognize and categorize comparisons where entities display unique attributes that resist simple comparison, aligning with our model's classification of 'Non-Gradable' comparisons. Including this category aims to provide a comprehensive overview of the spectrum of comparative relations our model is designed to analyze.

Our comprehensive evaluation of the CPC aspect underlines the robustness and versatility of the RoBERTa-pair-NLI-M model in deciphering complex comparative preferences across diverse datasets. This endeavor not only validates the model's efficacy in handling nuanced comparative queries but also paves the way for further explorations into enhancing its interpretative depth and accuracy.

////////////////////////////////////////////////////////////////////////////////////////////////////////////

Table 26 displays relatively low values for the None class accompanied by
high numbers for the other two classes. What are the misclassifications
then? A detailed confusion matrix here could help to clarify.

Dear Editor,

Addressing the concern raised about Table 26 and the classification performance for the 'None' class, it's crucial to acknowledge our dataset's inherent class imbalance, with 85 instances of the 'Object' class, 1067 instances of the 'Subject' class, and only 8 instances of the 'None' class. This distribution presents a significant challenge in achieving equitable classification accuracy across all classes, particularly for the underrepresented 'None' category.

To directly tackle the challenge posed by the class imbalance within our dataset, we combined the implementation of stratified k-fold cross-validation with the strategic application of Focal Loss, a method expressly designed to boost the model's sensitivity to less represented classes. Stratified k-fold cross-validation deviates from the standard approach by ensuring each fold mirrors the dataset's overall class distribution, which is indispensable for the precise classification of the 'None' category. Simultaneously, our customized application of Focal Loss further targets this imbalance. By adjusting the loss contribution from difficult-to-classify examples, Focal Loss significantly enhances the overall classification performance, especially for underrepresented classes. We operationalized this by introducing an alpha tensor, alpha = torch.tensor([1, 1, 2], dtype=torch.float).to(device), thereby increasing the weight for the 'None' class within the learning process. This adjustment is encapsulated in our loss function definition: criterion = FocalLoss(alpha=alpha, gamma=2.0).to(device). This dual strategy—merging stratified k-fold cross-validation with Focal Loss—underlines our comprehensive approach to addressing the nuanced challenges of class imbalance, particularly elevating the model's performance in classifying the infrequent 'None' category.

The Overall Classification Report and Confusion Matrix for one of our execution models, which is accessible via the provided GitHub link, underscores the impact of these methodological refinements:

Overall Classification Report:

precision	recall f1-score	support

0	0.9351	0.8471	0.8889	85

1	0.9897	0.9953	0.9925	1067

2	0.6000	0.7500	0.6667	8

accuracy	0.9828	1160

macro avg	0.8416	0.8641	0.8494	1160

weighted avg	0.9831	0.9828	0.9827	1160

Overall Confusion Matrix:

[[ 72	10	3]

[	4 1062	1]

[	1	1	6]],

These results reflect substantial improvements in classification accuracy, especially for the 'None' class, evidencing our concerted efforts to enhance model performance across all categories.

Despite the effectiveness of focal loss and the transition to stratified k-fold cross-validation in mitigating class imbalance, we recognize the potential benefits of further dataset augmentation. We are prepared to enrich our dataset with additional instances of the 'None' class to optimize classification outcomes and ensure a more balanced representation across classes.

We apologize for not including this critical explanation in our initial manuscript submission. In the revised manuscript, we have added these details, aiming to provide a complete and transparent view of our efforts to address class imbalance and enhance the model's performance across all categories.

We trust this comprehensive response, incorporating our focal loss application, stratified k-fold cross-validation explanation, and associated classification metrics, addresses the initial concerns raised. We appreciate this opportunity to clarify our approach and remain committed to the highest standards of research integrity and transparency.

Thank you for allowing us the opportunity to clarify these aspects of our study. We appreciate your guidance and look forward to your feedback.

**To honor the reviewer's input and ensure the thoroughness of our response, we also include this addition here in our response letter:**

**Revised Section 4.3. Compared Elements Identification (CEI)**

CEI involves three stages: creating auxiliary sentences based on a given question and its Entities and Aspects, adapting the Entity Role Identification (ERI) into a sentence-pair classification and refining the RoBERTa model for ERI. This process concludes by consolidating relations into 4-tuple comparative sets for each question. Assuming accuracy in the Element Extraction (EE) phase, the approach focuses on precise classification. Entity roles in questions are categorized as Subject, Object, or None. To address the inherent class imbalance between the 'Subject' class and the 'None' class, our model implements stratified k-fold cross-validation coupled with Focal Loss. This methodology is specifically designed to enhance classification accuracy for underrepresented classes. Evaluation on the SCQRD dataset yielded a micro-F-score of 0.802% (Table 20). Class analysis indicates superior performance of the "Subject" class over the "None" class, especially in multi-relation questions (Table 21).

Fig 6 compares the CEI model to two other models on the SCQRD and Camera-COQE datasets. Camera-COQE was chosen for its unique annotations. Performance ranks are: Proposed CEI model > COQE > Xu et al. [14]. The model's advantage over COQE comes from RoBERTa's enhanced capabilities and a targeted classification approach.

////////////////////////////////////////////////////////////////////////////////////////////////////////////

Errors in text:

line 50, &quot;we fine-tuned the SCRQD dataset&quot; -&gt; dataset is not fine-tuned

line 54, &quot;superiority over existing model&quot; -&gt; &quot;models&quot;

line 156, &quot;REDDY and KMahesh&quot; -&gt; &quot;Reddy and Mahesh Babu&quot;

line 162, &quot;et al. [11]&quot; -&gt; &quot;Liu et al. [11]&quot;

Table 6, &quot;(Q2, 4-tuple2)&quot; in Example 1 and &quot;(Q1, 4-tuple1)&quot; in Example 2
incorrectly refer to the other question

Table 7. with Illustration of BIO tags is completely mistaken leading to
entity &quot;then iPhone&quot;, aspects &quot;better&quot; and &quot;a&quot;, and constraints &quot;Samsung
smartphone&quot; and &quot;12 with a price below 600&quot;.

line 382, &quot;As highlighted by Sun et al. [23]&quot; - [23] is Devlin et al.

line 576, &quot;questions that assessing the intensity or degree&quot; -&gt; &quot;that assess&quot;

line 769, &quot;p_i intersection g_i&quot; - not all quintuple elements are sets, are they?

line 779, &quot;The Binary matching strategy emerged as the most efficient&quot;
- claiming that the most permissive evaluation is &quot;efficient&quot; is strange

Dear Reviewer,

Thank you very much for your detailed feedback and for highlighting specific areas in the manuscript that require correction or clarification. We appreciate the opportunity to refine our work and enhance its accuracy and clarity. In response to your comments, we have divided our corrections and clarifications into two sections for a structured discussion.

**Section 1: Corrections and Clarifications (Cases 1-8 and 10)**

Dear Reviewer,

Thank you for your meticulous reading of our manuscript and for pointing out the errors. We acknowledge the errors and the need for clarifications you've pointed out in lines 50, 54, 156, 162, as well as the inaccuracies in Table 6, Table 7, line 382, and line 576 of our manuscript. Here are the adjustments we have made:

1. **Line 50 Correction**: We have revised the text to accurately reflect the action performed: "By leveraging a RoBERTa-based transformer architecture, we fine-tuned our model on the SCRQD dataset." This corrects the mistaken notion of fine-tuning a dataset.
2. **Line 54 Correction**: The phrase has been corrected to: "Moreover, experiments on SemEval 2014, Camera-COQE, and CompSent-19 datasets revealed its superiority over existing models." This amendment reflects the comparison with multiple models.
3. **Line 156 Correction**: We have corrected the authors' names to "Reddy and Mahesh Babu" to correctly attribute their work.
4. **Line 162 Correction:** The citation has been updated to "Liu et al. [11]" for proper acknowledgment of the authors.
5. **Table 6 Correction:** The associations in Example 1 and Example 2 were interchanged. We have corrected the references to accurately match "(Q1, 4-tuple1)" with Example 1 and "(Q2, 4-tuple2)" with Example 2.
6. **Table 7 Correction:** We have thoroughly revised the illustration of BIO tags to ensure that it correctly identifies entities, aspects, and constraints without leading to incorrect interpretations such as "then iPhone", "better", "a", "Samsung smartphone", and "12 with a price below 600".
7. **Line 382 Correction:** The incorrect citation " Sun et al." has been replaced with the accurate reference to properly cite the relevant work.
8. **Line 576 Correction:** We have corrected the grammatical error to: "The X-type preference represents another innovative classification, specifically tailored for questions that assess the intensity or degree of preference between two compared entities."

10**. Line 779 Correction:** We have altered the statement regarding the Binary matching strategy to remove any implication of efficiency. The revised statement now reads: "The Binary matching strategy proved to be the most permissive, which in the context of our evaluation, was followed by the Proportional and then the Exact strategies in terms of performance."

We hope that these corrections meet your expectations and improve the clarity and accuracy of our manuscript.

**Section 2: Detailed Discussion on Case 9 (Line 769 and the Use of Intersection)**

Dear Reviewer,

Thank you for your insightful query regarding our use of intersection in the evaluation metrics, particularly in relation to the assumption that all quintuple elements are treated as sets. Your question highlights an important aspect of our methodology that warrants clarification.

In our manuscript, the use of intersection for computing the #correct in Binary and Proportional matches was intended to conceptualize the overlap between predicted and golden quintuple comparative relations. This approach was adopted to accommodate the variability in comparative element representation and to provide a flexible framework for evaluating our model's performance across different matching strategies.

Upon reflection, and considering your valuable feedback, we recognize that describing these elements strictly as sets may inadvertently imply a broader generality than is applicable in our context. To address this and improve clarity:

**We will refine our explanation in section 4.5.1. Evaluation Results** to specify that the intersection operation is metaphorically used to represent the matching process between predicted and golden annotations. The goal is to assess the degree of overlap, rather than to imply a strict set-theoretical operation applicable to all quintuple components.

**Revised Context:**

"In assessing the accuracy of our model's predictions against the golden annotations, we employ a metaphorical interpretation of intersection to evaluate the overlap for Binary and Proportional matches. This approach allows us to flexibly quantify the extent of agreement between the model's predictions and the dataset annotations, accommodating the nuanced representation of comparative relations. Specifically, we calculate #correct as a measure of this overlap, where a 'match' reflects a correspondence that signifies a correct prediction within the scope of each evaluation strategy."

This clarification aims to dispel any ambiguity around the application of intersection in our evaluation metrics and reaffirms our methodological intent to provide a comprehensive and adaptable evaluation framework for SCRE.

We hope this response addresses your concern and appreciate the opportunity to refine our manuscript for greater precision and understanding.

**To honor the reviewer's input and ensure the thoroughness of our response, we also include this addition here in our response letter:**

**Revised Section 4.5.1. Evaluation Results**

The primary task is presenting the results related to SCRE. Table 25 categorizes the scores, focusing on the EE (comprising Entity, Aspect, and Constraint extraction), CEI, and CPC subtasks. It summarizes the F-score of each specific subtask and their overall micro-average F-score, all based on five independent runs on the SCQRD dataset.

The detailed task of SCRE, following the methodologies in [5], requires the computation of Precision, Recall, and F1_score for all Quintuples. The formulas to obtain these metrics are as depicted in (1):

$Precision=\frac{\#correct}{\#predict}$, $Recall =\frac{\#correct}{\#gold}$, $F1score=\frac{2 \times Precision \times Recall}{Precision + Recall} (1)$

Where #predict represents the number of quintuple comparative relations (Subject Entity, Object Entity, Compared Aspect, Constraint, Comparative Preference) predicted by the model, #gold denotes the quintuple comparative relations in the dataset, and #correct is the count of correct quintuple comparative relations in the predictions.

Following the methodology from [5], we employ Precision, Recall, and F-score metrics using three matching strategies: Exact, Proportional, and Binary matching. An Exact match is a strict method where predicted comparative elements must exactly match the golden annotations to be considered correct. Binary and Proportional matches are more lenient. The Binary match checks if all elements of a predicted quintuple overlap with the golden annotation. The Proportional match computes the maximum overlap ratio between predicted and golden elements.

In assessing the accuracy of our model's predictions against the golden annotations, we employ a metaphorical interpretation of intersection to evaluate the overlap for Binary and Proportional matches. This approach allows us to flexibly quantify the extent of agreement between the model's predictions and the dataset annotations, accommodating the nuanced representation of comparative relations. Specifically, we calculate #correct as a measure of this overlap, where a 'match' reflects a correspondence that signifies a correct prediction within the scope of each evaluation strategy.

Accordingly, #correctE, #correctP, and #correctB for Exact, Proportional, and Binary matches are as depicted in (2)-(4):

$\#{correct}_{E}=\left\{ \begin{aligned} 0 \exists\left( p_{i}\neq g_{i} \right); \\ 1 otherwise \end{aligned} \right.$	$(2)$	, $\#{correct}_{B}=\left\{ \begin{aligned} 0 \exists\left( p_{i}\bigcap g_{i} \right)=Ø; \\ 1 otherwise \end{aligned} \right.$	$(3)$

$\#{correct}_{P}=\left\{ \begin{aligned} 0 \exists\left( p_{i}\bigcap g_{i} \right)=Ø \\ \frac{\sum_{i} len\left( p_{i}\bigcap g_{i} \right)}{\sum_{i} len\left( g_{i} \right)} otherwise \end{aligned} \right.$	$(4)$

Here, $p_{i}$ and $g_{i}$ represent the i-th element in the predicted and gold quintuple comparative relations, respectively, while len(·) indicates the length of the comparative element.

Fig 10 plots the performance of the model under these strategies, focusing on Precision, Recall, and F-score, based on five independent runs on the SCQRD dataset. Interestingly, the results suggest a higher recall than precision, indicating a tendency for negative-to-positive misclassifications. The Binary matching strategy proved to be the most permissive, which in the context of our evaluation, was followed by the Proportional and then the Exact strategies in terms of performance.

Separate sub-tasks and the overall SCRE task in a pipeline present contrasting evaluation results. The integrated approach has its limitations, primarily due to the cumulative errors across stages.

6. PLOS authors have the option to publish the peer review history of their article ([what does this mean?](https://journals.plos.org/plosone/s/editorial-and-peer-review-process#loc-peer-review-history)). If published, this will include your full peer review and any attached files.


If you choose &ldquo;no&rdquo;, your identity will remain anonymous but your review may still be made public.


**Do you want your identity to be public for this peer review?** For information about this choice, including consent withdrawal, please see our [Privacy Policy](https://www.plos.org/privacy-policy).

Reviewer #1: No

[NOTE: If reviewer comments were submitted as an attachment file, they will be attached to this email and accessible via the submission site. Please log into your account, locate the manuscript record, and check for the action link &quot;View Attachments&quot;. If this link does not appear, there are no attachment files.]

While revising your submission, please upload your figure files to the Preflight Analysis and Conversion Engine (PACE) digital diagnostic tool, <https://pacev2.apexcovantage.com/>. PACE helps ensure that figures meet PLOS requirements. To use PACE, you must first register as a user. Registration is free. Then, login and navigate to the UPLOAD tab, where you will find detailed instructions on how to use the tool. If you encounter any issues or have any questions when using PACE, please email PLOS at [figures@plos.org](mailto:figures@plos.org). Please note that Supporting Information files do not need this step.

<span style="font-style: italic; font-size: 90%;">In compliance with data protection regulations, you may request that we remove your personal registration details at any time. [(Remove my information/details)](https://www.editorialmanager.com/pone/login.asp?a=r). Please contact the publication office if you have any questions.
