# Supplementary material for: SCRQE: Subjective comparative relation quintuple extraction from questions in product domain
Source: PLoS One. 2025 May 27;20(5):e0319824. doi: 10.1371/journal.pone.0319824 (PMC12112347; doi:10.1371/journal.pone.0319824)
Supplement: Attachment — Submitted filename: Detailed_Response_to_Reviewers_auresp_2.docx [file pone.0319824.s005.docx]

**View Letter**

| **Date:** | Jun 11 2024 09:30PM |
| --- | --- |
| **To:** | "Afsaneh Fatemi" a_fatemi@eng.ui.ac.ir |
| **From:** | "PLOS ONE" plosone@plos.org |
| **Subject:** | PLOS ONE Decision: Revision required [PONE-D-23-39359R1] |

PONE-D-23-39359R1

SCRQE: Subjective Comparative Relation Quintuple Extraction from Questions in Product Domain

PLOS ONE

Dear Dr. Fatemi,

Thank you for submitting your manuscript to PLOS ONE. After careful consideration, we feel that it has merit but does not fully meet PLOS ONE’s publication criteria as it currently stands. Therefore, we invite you to submit a revised version of the manuscript that addresses the points raised during the review process.

Please submit your revised manuscript by Jul 26 2024 11:59PM. If you will need more time than this to complete your revisions, please reply to this message or contact the journal office at <a href="mailto:plosone@plos.org">plosone@plos.org. When you're ready to submit your revision, log on to https://www.editorialmanager.com/pone/ and select the 'Submissions Needing Revision' folder to locate your manuscript file.

Please include the following items when submitting your revised manuscript:

- A rebuttal letter that responds to each point raised by the academic editor and reviewer(s). You should upload this letter as a separate file labeled 'Response to Reviewers'.
- A marked-up copy of your manuscript that highlights changes made to the original version. You should upload this as a separate file labeled 'Revised Manuscript with Track Changes'.
- An unmarked version of your revised paper without tracked changes. You should upload this as a separate file labeled 'Manuscript'.

If you would like to make changes to your financial disclosure, please include your updated statement in your cover letter. Guidelines for resubmitting your figure files are available below the reviewer comments at the end of this letter.

If applicable, we recommend that you deposit your laboratory protocols in protocols.io to enhance the reproducibility of your results. Protocols.io assigns your protocol its own identifier (DOI) so that it can be cited independently in the future. For instructions see: <https://journals.plos.org/plosone/s/submission-guidelines#loc-laboratory-protocols>. Additionally, PLOS ONE offers an option for publishing peer-reviewed Lab Protocol articles, which describe protocols hosted on protocols.io. Read more information on sharing protocols at <https://plos.org/protocols?utm_medium=editorial-email&utm_source=authorletters&utm_campaign=protocols>.

We look forward to receiving your revised manuscript.

Kind regards,

Leona Cilar Budler
Academic Editor
PLOS ONE

### Response to Editor's Invitation for Manuscript Revision

Dear Dr. Leona Cilar Budler,

We sincerely appreciate the thoughtful evaluation of our manuscript and the opportunity to revise and resubmit it for consideration in **PLOS ONE**. The constructive comments and suggestions from the review process are invaluable, and we are confident that addressing them will significantly enhance the clarity and impact of our research.

After carefully reviewing the feedback, we have made substantial revisions to our manuscript, focusing particularly on improving methodological clarity, providing stronger context and justification for our work, and incorporating more relevant comparisons with recent advancements in the field, as advised.

In line with your submission guidelines, we are prepared to submit the revised manuscript by the provided deadline, along with the following documents:

1. **Rebuttal Letter**: A detailed response addressing each point raised by the academic editor and reviewers, which will be uploaded as a separate file labeled 'Response to Reviewers.'
2. **Marked-Up Manuscript**: A version of the manuscript with all changes clearly tracked for ease of review, to be submitted as 'Revised Manuscript with Track Changes.'
3. **Unmarked Manuscript**: A clean version of the revised manuscript without tracked changes, labeled 'Manuscript.'

Additionally, we are pleased to inform you that our datasets and code are available on GitHub at: https://github.com/mahsamb/SCRQD. We believe this transparency will contribute to the reproducibility of our research.

We will also revisit our financial disclosure statement and update it, if necessary, in the cover letter.

Regarding the recommendation to deposit our laboratory protocols on **protocols.io**, we recognize the importance of promoting reproducibility and transparency in research. We plan to deposit our protocols accordingly and contribute to these best practices in scientific research.

We are excited to move forward with the revision process and are grateful for the guidance and support provided by **PLOS ONE**. Should you require any additional information or clarification, please do not hesitate to contact us.

Warm regards,
Dr. Afsaneh Fatemi

**Additional Editor Comments:**

There are some suggestions and comments from reviewers to improve your paper. Please take them into account to improve your paper.

### ****Response to Editor's Additional Comments:****

Dear Editor,

Thank you for your additional comments regarding the reviewer feedback. We appreciate the suggestions provided by the reviewers, and we will thoroughly address each point in our revised manuscript. We are committed to ensuring that all the recommendations are taken into account to improve the quality and clarity of our paper.

We will provide a detailed response to each of the reviewers' comments in the rebuttal letter, alongside the revised manuscript, as per the submission guidelines.

Once again, thank you for your guidance and for the opportunity to enhance our manuscript.

Warm regards,
Dr. Afsaneh Fatemi

[Note: HTML markup is below. Please do not edit.]

Reviewers' comments:

Reviewer's Responses to Questions

### ****Opening Response to the Reviewers:****

Dear Reviewers,

Thank you for your insightful comments and constructive critique. We sincerely appreciate your thorough review and the opportunity to refine the sections you highlighted for improvement. Our commitment lies in enhancing the quality and transparency of our manuscript, and we have made revisions accordingly based on your valuable suggestions.

Additionally, we are pleased to inform you that our datasets and code are available on GitHub at: https://github.com/mahsamb/SCRQD. We believe this transparency will contribute to the reproducibility of our research.

Warm regards,
Dr. Afsaneh Fatemi

**Comments to the Author**

1. If the authors have adequately addressed your comments raised in a previous round of review and you feel that this manuscript is now acceptable for publication, you may indicate that here to bypass the “Comments to the Author” section, enter your conflict of interest statement in the “Confidential to Editor” section, and submit your "Accept" recommendation.

Reviewer #1: (No Response)

Reviewer #2: All comments have been addressed

2. Is the manuscript technically sound, and do the data support the conclusions?

The manuscript must describe a technically sound piece of scientific research with data that supports the conclusions. Experiments must have been conducted rigorously, with appropriate controls, replication, and sample sizes. The conclusions must be drawn appropriately based on the data presented.

Reviewer #1: Partly

Reviewer #2: Yes

3. Has the statistical analysis been performed appropriately and rigorously?

Reviewer #1: N/A

Reviewer #2: N/A

4. Have the authors made all data underlying the findings in their manuscript fully available?

The [PLOS Data policy](http://www.plosone.org/static/policies.action#sharing) requires authors to make all data underlying the findings described in their manuscript fully available without restriction, with rare exception (please refer to the Data Availability Statement in the manuscript PDF file). The data should be provided as part of the manuscript or its supporting information, or deposited to a public repository. For example, in addition to summary statistics, the data points behind means, medians and variance measures should be available. If there are restrictions on publicly sharing data—e.g. participant privacy or use of data from a third party—those must be specified.

Reviewer #1: Yes

Reviewer #2: Yes

5. Is the manuscript presented in an intelligible fashion and written in standard English?

PLOS ONE does not copyedit accepted manuscripts, so the language in submitted articles must be clear, correct, and unambiguous. Any typographical or grammatical errors should be corrected at revision, so please note any specific errors here.

Reviewer #1: Yes

Reviewer #2: Yes

6. Review Comments to the Author

Please use the space provided to explain your answers to the questions above. You may also include additional comments for the author, including concerns about dual publication, research ethics, or publication ethics. (Please upload your review as an attachment if it exceeds 20,000 characters)

Reviewer #1: The authors have provided a substantial revision of the original text, adding
a number of previously missing details of the work. While the new text has
significantly improved the critical points of the authors' work, several
new questions arise accompanying the added details.

### Addressing Reviewer Feedback: Clarifications and Revisions

**Dear Reviewer,**

Thank you for your thorough review and valuable feedback. We sincerely appreciate the time and effort you have invested in evaluating our work. Your insights have provided us with an invaluable opportunity to refine our manuscript and clarify key aspects of our research. We are grateful for the chance to address your concerns and to offer further clarification on our motivations, methodologies, and the impact of our proposed changes. Below are our detailed responses to each of your comments, along with explanations of the revisions we have made to enhance the clarity, accuracy, and overall quality of our work.

One of the crucial points lies in the subject difference to most existing
datasets, i.e. to working with comparative questions instead of comparative
sentences. The authors point at the differences (and complications) between
these two data approaches, but a motivation for processing questions is
missing. A possible reason could be connected with "answers", that are
natural companions of questions, but these are not touched at all in the
text.

Dear Reviewer,

Thank you for your insightful comments. We appreciate you highlighting the need for clearer motivation regarding our focus on comparative questions and the connection to answer generation. We have revised the manuscript to address these points.

**Motivation for Processing Questions Instead of Sentences**

The motivation for focusing on subjective comparative questions rather than sentences is rooted in the way users naturally interact with systems, particularly in e-commerce platforms and question-answering applications. In these contexts, users often pose comparative questions when they are in the process of making decisions, such as choosing between products or services. These questions typically seek to draw direct comparisons between two or more entities, reflecting the user's intent to evaluate specific features or aspects that are important to their decision-making process.

Comparative questions are more than just statements; they capture the active search for answers that keeps users engaged. For instance, when a user asks, "Is Product A better than Product B in terms of quality?" they are not merely stating a fact but are actively seeking information that will influence their purchase decision. This interaction is inherently dynamic and driven by specific goals, making the analysis of questions more pertinent to real-world applications compared to analyzing standalone sentences.

Our model, by concentrating on the analysis of questions, better captures user intent—preferences, priorities, and context—leading to more accurate and tailored responses. This approach not only improves user satisfaction but also serves as an essential initial step in the automatic question answering (AQA) process. Additionally, subjective comparative questions feature distinct comparative preference classifications (CPC) compared to sentences, further justifying the need for this focused, question-driven analysis.

In summary, focusing on subjective comparative questions aligns our model with real-world user interactions and aids in the development of more refined question-answering systems.

**The Role of Question Analysis in Answering Subjective Comparative Questions**

You are correct that in our initial manuscript, we did not fully explain the role of question analysis in answering subjective comparative questions. However, we have now addressed this in our revised manuscript by including a detailed discussion in the Introduction section. In our revised manuscript, we provide a comprehensive analysis of the role of question analysis in answering subjective comparative questions. Specifically, we focus on the detailed analysis of comparative questions as a crucial step in the automatic question answering (AQA) process, which involves identifying and classifying key elements such as subject and object entities, compared aspects, comparative preferences, and constraints. By accurately determining these elements, our model ensures that answers are relevant, contextually precise, and aligned with the user's intent, ultimately assisting users in making informed decisions. This foundational analysis is essential for generating meaningful and actionable answers in subsequent stages of AQA, and provides valuable insights that can be leveraged by other research efforts aimed at answer generation. By incorporating these elements into our analysis, we ensure that the insights generated can be effectively used by other systems to produce meaningful, actionable answers that align with user preferences and improve decision-making.

**To honor the reviewer's input and ensure the thoroughness of our response, we have expanded the Introduction in our revised manuscript (lines 71-128) to provide a detailed discussion on the motivation for focusing on comparative questions rather than sentences:**

Our model's outputs, particularly the determination of subject and object entities, the identification of compared aspects, the classification of comparative preferences (CPC), and the extraction of constraints, play a pivotal role in shaping the content and form of the answers:

1. **Determining Subject/Object Entities:** By accurately identifying the entities being compared, our model ensures that the answer is directly relevant to the user's query, focusing on the correct entities without ambiguity.
2. **Compared Aspects:** The identification of specific aspects under comparison allows the generated answer to be precise and contextually relevant, addressing exactly what the user is interested in, such as performance, design, or price.
3. **Comparative Preference Classification (CPC) of Questions:** The CPC process categorizes the nature of the comparison within the question, such as whether one entity is "better," "worse," or "equal" to another, or more nuanced preferences like "significantly better" or "marginally worse." This classification profoundly influences the answer generated in response to a subjective comparative question, as it directly shapes the direction, tone, and content of the answer.

- **Alignment of Answer Tone with Question’s Intent:** By identifying the specific comparative preference expressed in the question, the CPC helps ensure that the answer's tone aligns with the user’s intent. For example, if a question asks whether a product is "significantly better" than another, the answer can affirm this with strong, decisive language if the comparison holds true, or gently refute it with explanations if it does not. This alignment is critical in providing an answer that resonates with the user’s expectations and perceptions.
- **Focus on Relevant Aspects:** The CPC of the question also guides the answer to focus on the aspects most relevant to the user. For instance, if a question compares two smartphones primarily on their camera quality, and the CPC indicates a "worse" comparison, the answer can delve into the specific shortcomings of the camera in one smartphone compared to the other, ensuring that the response is directly relevant to the user’s concerns.
- **Handling Ambiguities and Preferences:** In cases where the CPC reveals ambiguous or mixed preferences in the question (e.g., "Is the camera quality of Phone A better, but its battery life worse than Phone B?"), the answer can address each aspect separately, providing a balanced view that helps the user understand the trade-offs. This nuanced handling of preferences ensures that the answer is comprehensive and addresses all facets of the user's query.
- **Enhancing Decision-Making with Nuanced Comparisons:** The CPC of the question can highlight nuanced preferences, such as a slight advantage in one area versus a significant disadvantage in another. This allows the answer to help the user weigh these factors in their decision-making process. For example, if a question asks whether a laptop is "slightly better in performance but much worse in portability" than another, the answer can guide the user by emphasizing the relative importance of these aspects based on their likely priorities.
- **Customizing Answer Length and Detail:** The CPC can also influence the length and detail of the answer. For instance, a question classified with a "strong preference" might warrant a more detailed explanation to justify this classification, whereas a question with an "equal" classification might receive a more concise response, reflecting the lack of a clear distinction between the compared entities.

4. **Constraint Extraction:** Constraints such as price limits or specific conditions (e.g., under low light conditions) are crucial for tailoring the answer to the user's needs, making the information provided more useful and applicable in specific contexts.

Once again, thank you for bringing this to our attention and for helping us improve the clarity and thoroughness of our manuscript.

The problems can be seen also in the dataset preparation procedure
where the authors admit that "due to the scarcity of questions that exactly
matched our research criteria, we modified existing questions and
formulated new ones to diversify our dataset" (line 511).

**Enhancements to Dataset and Annotation Procedures**

Dear Reviewer,

Thank you for raising the important point about the dataset preparation procedure. We acknowledge the initial limitations due to the scarcity of readily available questions perfectly matching our research criteria. To address this, we have significantly expanded and diversified our dataset and revised our annotation process.

Specifically, we have:

- **Added 1025 questions:** We augmented the original SCRQD dataset with 1025 new questions, creating the Smartphone-SCQRE dataset. This addition increases the size and diversity of the data, reflecting more realistic usage scenarios.
- **Revised the annotation process:** Three PhD students specializing in NLP and experienced in annotation re-annotated the entire dataset. This ensures higher accuracy and reliability for the subjective comparative quintuple extraction task.
- **Incorporated an established dataset:** We leveraged the CompSent-19 dataset (brands domain), converting relevant entries into question format and annotating them as quintuples. This created the Brands-CompSent-19-SCQRE dataset (927 questions), further diversifying the data in terms of domain and providing a benchmark against existing work.

These enhancements address the initial limitations of the dataset and provide a more robust and diverse foundation for evaluating our model. The inclusion of real-world questions, the rigorous annotation process, and the incorporation of an established dataset strengthen the validity and generalizability of our findings.

**Leveraging External Datasets for Comprehensive Model Evaluation**

Given the absence of an appropriate question-based dataset for our evaluation, we took proactive steps to ensure that our model could be thoroughly tested. We selected two well-known datasets frequently used for CPC tasks—the Camera-COQE dataset and one domain (brands) from the CompSent-19 dataset. To make these datasets suitable for our analysis, we first identified subjective comparative questions within them. We then converted these questions into the required format and annotated them in quintuples for our model.

By preparing these additional datasets, we aimed to provide a thorough evaluation of our model's performance, showcasing its effectiveness in processing and categorizing a variety of comparative questions.

**To honor the reviewer's input and ensure the thoroughness of our response, we also include this addition here in our response letter:**

**3.2.1 Dataset Overview**

To the best of our knowledge, the only available dataset related to SCRE from comparative questions was provided by Yu et al. [2], containing 220 mobile domain questions. Of these, only 35 are subjective comparative and lack constraint and preference intensity annotations. To address this shortage, we introduced two datasets: the "Smartphone-SCQRE" dataset and the "Brands-CompSent-19-SCQRE" dataset.

The Smartphone-SCQRE dataset consists of 2,300 manually annotated subjective comparative questions collected from sources like Quora. The "Brands-CompSent-19-SCQRE" dataset contains 927 questions, adapted from the CompSent-19 dataset, and was selected to evaluate the robustness and generalizability of our model in handling subjective comparative questions from a different domain. We processed the subjective comparative sentences in this dataset, converted them into question format, and applied the same quintuple annotation structure as Smartphone-SCQRE, including the subject entity, object entity, compared aspect, constraint, and preference. Since the number of sentences from that domain meeting our criteria for subjective comparative forms was low, in some cases, we generated multiple questions from one sentence, using various subjective comparative forms—including target, yes/no, attitude, and polarity—as outlined in Babaali et al. [24], with different comparative preferences.

For both datasets, three annotators, experienced in sentiment analysis and proficient in English, labeled each question, with the majority vote determining the final tags. The dataset statistics for CEE, CPC, and SCRE tasks are provided in Tables 6, 7, and 8 for both datasets.

**Table 6.** Statistics for the Smartphone-SCQRE and Brands-CompSent-19-SCQRE Datasets Regarding the CEE Task

| **Elements** | **Info** | **Smartphone-SCQRE** | **Brands-CompSent-19-SCQRE** |
| --- | --- | --- | --- |
| **Questions** | # Questions | 2300 | 927 |
| **Entity** | # Entities | 5802 | 2900 |
|  | # Single-Word Entities | 595 | 1910 |
|  | # Multi-Word Entities | 4817 | 990 |
|  | Max Multi-Word | 9 | 7 |
|  | Subject Entity | 3629 | 1565 |
|  | Object Entity | 1783 | 1335 |
| **Aspect** | # Aspects | 2557 | 1052 |
|  | # None-Generalized "features" | 2006 | 634 |
|  | # Generalized "features" | 551 | 418 |
|  | # Single-Word Aspects | 577 | 402 |
|  | # Multi-Word Aspects | 1429 | 232 |
|  | Max Multi-Word | 7 | 5 |
| **Constraint** | # Constraints | 301 | 268 |
|  | # Single-Word Constraints | 39 | 25 |
|  | # Multi-Word Constraints | 262 | 243 |
|  | Max Multi-Word | 13 | 12 |

In Table 6, **#** Generalized 'features' refers to explicit aspects mentioned in the question, while **#** None-Generalized 'features' applies to cases where no specific aspect is present, and a generalized "features" label is assigned.

**Table 7.** Statistics for the Smartphone-SCQRE and Brands-CompSent-19-SCQRE Datasets Concerning the CPC Task.

| **Preference Type** | **Smartphone-SCQRE** | **Brands-CompSent-19-SCQRE** |
| --- | --- | --- |
| Better | 331 | 296 |
| Strong Better | 275 | 116 |
| Equal | 162 | 53 |
| Worse | 156 | 72 |
| Strong Worse | 112 | 53 |
| XOR-Strong Better | 129 | 53 |
| XOR-Better | 271 | 41 |
| XOR-Equal | 122 | 45 |
| XOR-Worse | 134 | 53 |
| XOR-Strong Worse | 220 | 47 |
| X-Strong Better | 128 | 55 |
| X | 202 | 60 |
| X-Strong Worse | 129 | 52 |
| Non-Gradable | 189 | 56 |
| **Total** | **2560** | **1052** |

**Table 8.** Statistics for the Smartphone-SCQRE and Brands-CompSent-19-SCQRE Datasets Related to the SCRE Task.

| **Info** | **Smartphone-SCQRE** | **Brands-CompSent-19-SCQRE** |
| --- | --- | --- |
| # One-Relation Questions | 2058 | 821 |
| # Two-Relation Questions | 229 | 93 |
| # Three-Relation Questions | 12 | 7 |
| # Four-Relation Questions | 1 | 6 |

Once again, thank you for bringing this to our attention and for helping us improve the quality and robustness of our dataset and annotation procedures.

This motivation also concerns the two main data format differences to
previous approaches, i.e. the subject-object distinction (or the CEI task)
and the detailed comparison classification (or the CPC task) to 14 categories
instead of just Better/Worse. The impact of these changes is not evaluated
in the text.

Dear Reviewer,

Thank you for your valuable feedback regarding the subject-object distinction and the detailed comparison classification. We have revised the manuscript to address these points and further evaluate the impact of these changes.

### Dataset Expansion and Refinement of Subject-Object Distinctions

Regarding the concern about the subject-object distinction, we would like to clarify that in our previous manuscript, the "Subject-Subject" case type was applied in situations where entities were either "Equal" or involved in **XOR-type** comparative preferences, where the order of the compared entities did not matter. This "Subject-Subject" classification was used to reflect cases of **equality** or **XOR-type** questions where the entities are interchangeable in terms of comparison.

In response to the comment about the dataset statistics for the "None" role type, we have made significant updates. We expanded our dataset from 1,275 questions to 2,300 questions, now named **Smartphone-SCQRE**, allowing for a more comprehensive analysis. As a result of this expansion, the percentage of cases classified as "None" has increased to 18%, reflecting a more diverse range of comparative questions.

Additionally, we have prepared a new dataset, **Brands-CompSent-19-SCQRE**, comprising 927 questions, with 17% of cases involving the "None" role type. This revised categorization and the expansion of our datasets provide a more accurate and nuanced understanding of the roles entities play in comparative questions. These changes have been incorporated into our revised dataset and model evaluations to better address the distribution of subject-object distinctions.

**To honor the reviewer's input and ensure the thoroughness of our response, we also include this addition here in our response letter:**

**3.3.3.1. Entity Role Identification (ERI)**

At the core of the CEI phase, the ERI process is instrumental in assigning roles to entities as "Subject", "Object", or "None". This classification is crucial for accurately distinguishing between subject and object entities, which cannot be solely achieved through BIO labeling used in the first phase for the following reasons:

1) **Variability in Entity Roles**: Entities within a single question may not always serve as subjects or objects in the comparative relation, necessitating a dedicated mechanism for role identification.
2) **Multiple Comparative Relations:** Subjective comparative questions often involve multiple comparative relations within a single context, requiring a method to differentiate entity roles across these relations.
3) **Entities without Roles**: Some entities may not have subject/object roles but still relate to specific aspects, such as "quality display" or "RAM," as shown in Table 15, Case 8.

The ERI process leverages contextual cues and linguistic patterns specific to subjective comparative questions to ensure accurate role identification. In cases where entities are being compared for equality or using an XOR-type comparison (where the order doesn't matter), both entities are assigned the "Subject" role. This "Subject-Subject" classification reflects the interchangeable nature of the entities in these comparisons. This enhances the model’s ability to navigate the intricacies of comparative relations and deliver more accurate and nuanced results. This classification establishes a framework for mapping the structure of comparative relations within the dataset, encompassing:

- **Input Structuring for RoBERTa_base_go_emotions**: Inputs are methodically structured for RoBERTa_base_go_emotions by concatenating each question with auxiliary sentences, separated by RoBERTa_base_go_emotions’s tokens ("<S>", "<\S>").
- **Auxiliary Sentence Generation**: Inspired by Sun et al. [32], the NLI-M (Natural Language Inference with Multiple outputs) method is employed for its ability to handle varied comparative nuances, such as "Strong Better/Worse." This approach ensures clarity in classifying entities and is detailed in Appendix A.
  - **Generalization of Aspects**: For questions without specified aspects, a generalized "features" label is applied (as in Table 15, Case 4 and 6).
  - **Classifying Implicit Preferences**: When relations in a question suggest preferences without explicitly stating them, these entities are classified as "subjects" (e.g., Table 15, Case 2, 3, and 6).
- **Adaptation of RoBERTa_base_go_emotions for ERI:** A fine-tuned RoBERTa_base_go_emotions model is used for sentence-pair classification to efficiently discern comparative roles.
- **Identification of Compared Aspects**: The ERI process also facilitates the identification of Compared Aspects, as the auxiliary sentences inherently link each entity's role to the corresponding aspect.

**Table 15.** Smartphone-SCQRE dataset samples utilizing NLI-M for CEI task

| **#** | **Question Context** | **Auxiliary Sentence Construction** | **Label Assignment** |
| --- | --- | --- | --- |
| **1** | *“Is the Samsung Galaxy M31 better than the Samsung Galaxy A50 in* ***software performance****?”* | “Samsung Galaxy M31 - software performance” | subject |
|  |  | “Samsung Galaxy A50 - software performance” | object |
| **2** | *“Does the Oppo F19 Pro have the same* ***sound quality*** *as the Realme X7?”* | “Oppo F19 Pro - sound quality” | subject |
|  |  | “Realme X7- sound quality” | subject |
| **3** | *“Which one is better for* ***taking pictures****: Google Pixel, Samsung Galaxy, or iPhone?”* | “Google Pixel - taking pictures” | subject |
|  |  | “Samsung Galaxy - taking pictures” | subject |
|  |  | “'iPhone - taking pictures” | subject |
| **4** | *“Is the Xiaomi Redmi Note 8 Pro the best compared to the Realme XT and Realme X2 Pro?”* | “Xiaomi Redmi Note 8 Pro – features” | subject |
|  |  | “Realme XT – features” | object |
|  |  | “Realme X2 Pro – features” | object |
| **5** | *“Is the Samsung Galaxy S7 better than the S6 regarding* ***RAM management*** *and* ***CPU*** ***performance****?”* | “Samsung Galaxy S7 - RAM management” | subject |
|  |  | “S6 - RAM management” | object |
|  |  | “Samsung Galaxy S7 - CPU performance” | subject |
|  |  | “S6 - CPU performance” | object |
| **6** | *“I want a smartphone. I like that the Samsung Galaxy A11 has a large* ***screen****, but I don't know if it will be as* ***reliable*** *as iPhone 8?”* | “Samsung Galaxy A11 – screen” | none |
|  |  | “iPhone 8 – screen” | none |
|  |  | “iPhone 8 – reliable” | subject |
|  |  | “Samsung Galaxy A11 – reliable” | subject |
| **7** | *“Does OnePlus 6 has been able to beat its ZenFone 5Z?”* | “OnePlus 6 – features” | subject |
|  |  | “ZenFone 5Z – features” | object |
| **8** | *“What are the best smartphones with* ***a built in stylus feature*** *with a good* ***quality display*** *and* ***RAM****, other than Samsung?”* | “Samsung - built in stylus feature” | none |
|  |  | “Samsung - quality display” | none |
|  |  | “Samsung – RAM” | none |

**Impact of Granular CPC**

We acknowledge that some of our previous CPC annotations were not as precise as they could have been. To improve accuracy, we expanded our dataset by adding 1025 additional questions and prepared one supplementary dataset: the CompSent-19 dataset (brand domain). Moreover, we revised our entire annotation process and involved three new annotators—PhD students specializing in NLP who are familiar with the annotation process—to ensure greater accuracy and reliability in the CPC task.

Our classification into 14 categories, including "Strong Worse," "Strong Better," and others, offers a more granular understanding of comparative preferences. This granularity is crucial for capturing the intensity and nuances of user preferences, which are often not fully conveyed by a simple Better/Worse dichotomy. In our revised manuscript, we have added new examples and refined previous ones to clarify the rationale behind these categories.

For instance, the distinction of "Strong Worse" is captured in the example "Why is the iPhone 10's camera vastly worse than the iPhone XS's?" compared to the simpler "Why is the iPhone 10's camera worse than the iPhone XS's?" Similarly, the category of "Strong Better" is illustrated by "Why is the iPhone XS's camera quality so much superior to the iPhone 10's?" as opposed to "Why is the iPhone XS's camera quality better than the iPhone 10's?" These distinctions reflect a significant perceived difference in quality and sentiment, offering a deeper insight into user preferences than what a simple "Worse" or "Better" classification would provide.

By accurately categorizing the comparative preference, our model provides valuable insights that can be leveraged by other systems to align their responses with the user's expectations and reflect the underlying sentiment more precisely. This ensures that question-answering systems can generate responses that better match the nuances of user preferences. In this regard, we added the following section in our revised introduction to discuss how the CPC profoundly influences answer generation in several ways:

- Alignment of Answer Tone with Question’s Intent
- Focus on Relevant Aspects
- Handling Ambiguities and Preferences
- Enhancing Decision-Making with Nuanced Comparisons
- Customizing Answer Length and Detail

**To honor the reviewer's input and ensure the thoroughness of our response, we also include this addition here in our response letter:**

3. **Comparative Preference Classification (CPC) of Questions:** The CPC process categorizes the nature of the comparison within the question, such as whether one entity is "better," "worse," or "equal" to another, or more nuanced preferences like "significantly better" or "marginally worse." This classification profoundly influences the answer generated in response to a subjective comparative question, as it directly shapes the direction, tone, and content of the answer.

- **Alignment of Answer Tone with Question’s Intent:** By identifying the specific comparative preference expressed in the question, the CPC helps ensure that the answer's tone aligns with the user’s intent. For example, if a question asks whether a product is "significantly better" than another, the answer can affirm this with strong, decisive language if the comparison holds true, or gently refute it with explanations if it does not. This alignment is critical in providing an answer that resonates with the user’s expectations and perceptions.
- **Focus on Relevant Aspects:** The CPC of the question also guides the answer to focus on the aspects most relevant to the user. For instance, if a question compares two smartphones primarily on their camera quality, and the CPC indicates a "worse" comparison, the answer can delve into the specific shortcomings of the camera in one smartphone compared to the other, ensuring that the response is directly relevant to the user’s concerns.
- **Handling Ambiguities and Preferences:** In cases where the CPC reveals ambiguous or mixed preferences in the question (e.g., "Is the camera quality of Phone A better, but its battery life worse than Phone B?"), the answer can address each aspect separately, providing a balanced view that helps the user understand the trade-offs. This nuanced handling of preferences ensures that the answer is comprehensive and addresses all facets of the user's query.
- **Enhancing Decision-Making with Nuanced Comparisons:** The CPC of the question can highlight nuanced preferences, such as a slight advantage in one area versus a significant disadvantage in another. This allows the answer to help the user weigh these factors in their decision-making process. For example, if a question asks whether a laptop is "slightly better in performance but much worse in portability" than another, the answer can guide the user by emphasizing the relative importance of these aspects based on their likely priorities.
- **Customizing Answer Length and Detail:** The CPC can also influence the length and detail of the answer. For instance, a question classified with a "strong preference" might warrant a more detailed explanation to justify this classification, whereas a question with an "equal" classification might receive a more concise response, reflecting the lack of a clear distinction between the compared entities.

Once again, thank you for your thoughtful suggestions and for helping us enhance the quality of our manuscript.

The examples of the
detailed comparison categories are not always clear, e.g. why in "Why is
the iPhone 10 camera inferior compared to the iPhone XS" denotes "inferior"
a "Strong Worse" category (line 942)?

**Correction of Labeling Error in CPC Task**

Dear Reviewer,

Thank you for highlighting the issue with the categorization in our manuscript.

Thank you for highlighting the issue with the categorization in our manuscript, specifically regarding the incorrect CPC label in line 942. The label "Strong Worse" was indeed a gross unintentional mistake when writing the manuscript. The correct label for the question " Why is the iPhone 10's camera inferior to the iPhone XS's?" should be "Worse." The term "inferior" aligns more appropriately with the "Worse" category rather than "Strong Worse." We sincerely apologize for this oversight and have corrected it in the revised manuscript. This correction ensures that the categorization accurately reflects the comparative intensity implied by the question, thereby improving the accuracy and consistency of our CPC task.

Once again, thank you for bringing this to our attention and for helping us improve the quality and robustness of our manuscript.

In line 272, the presented SCQRD dataset is said to enable "robust model
training and evaluations across different languages and domains". How? The
dataset is limited to English and to one domain.

### Updates to the Manuscript on Multi-Domain Support and Model Evaluation

Dear Reviewer,

Thank you for your comment regarding the SCQRD dataset and its applicability across different languages and domains. We appreciate the opportunity to address your concerns.

**Domain Adaptability:**

In the revised manuscript, we have expanded the dataset to encompass **two distinct domains**:

1. **Smartphone-SCQRE**, which focuses on the smartphone domain, and
2. **Brands-CompSent-19-SCQRE**, which includes comparative questions across a wider variety of brands and product types.

This expansion demonstrates the flexibility of our framework to handle multiple domains beyond just smartphones. The methodology for extracting subjective comparative relations remains consistent across both datasets, showcasing that the model can generalize effectively across different product categories. By expanding into another domain with Brands-CompSent-19-SCQRE, we have made the model more versatile and capable of broader applications.

Additionally, we expanded our work to include evaluations on **two sentence-based datasets** from different domains: Camera-COQE and CompSent-19. This evaluation further underscores the model's generalization capabilities and its effectiveness across different dataset structures and domains.

**Language Scalability:**

You are correct; we have removed the term "multilingual" from our description but retained the "multi-domain" aspect. Based on your valuable feedback, we have revised the manuscript accordingly.

While both datasets are currently in English, the structure of the annotations and the model’s architecture (leveraging pre-trained models like **RoBERTa_base_go_emotions**) is designed to be extendable to other languages with appropriate multilingual language models, such as **XLM-R** or **mBERT**. This allows for potential expansion into multilingual datasets in future work, providing further robustness for cross-lingual evaluations.

This is why we initially referred to "multilingual robustness"; however, based on your valuable feedback that correctly pointed out we had not directly tested it in other languages, we have removed this claim from the manuscript.

**Manuscript Revisions:**

To address your concerns and clarify these points, we have made the following revisions to the manuscript:

- **Replacement of Terminology**: We have replaced the phrase "robust model training and evaluations across different languages and domains" in line 272.
- **Explicit Multi-domain Support**: We have revised the manuscript to explicitly state our support for multiple domains by incorporating the **Smartphone-SCQRE**, **Brands-CompSent-19-SCQRE**, **Camera-COQE**, and **CompSent-19** datasets. This demonstrates the applicability of our framework across different domains, in addition to the original smartphone domain. These updates are reflected in the revised manuscript.
- **Dataset Expansion and Annotation Improvements**: We expanded our **Smartphone-SCQRE** dataset from **1,275** to **2,300** questions and introduced **Brands-CompSent-19-SCQRE** with **927** additional questions. Moreover, we enhanced our annotation process by involving **three new NLP-specialized annotators** to improve the accuracy and reliability of entity, aspect, constraint, and comparative preference annotations. These updates are detailed in **Section 2-3** of the revised manuscript.

We believe these revisions provide a clearer understanding of how the SCQRD dataset and our methodologies can support model training and evaluations across different domains, despite the current dataset's limitations.

Once again, thank you for your insightful comments. We appreciate your guidance in enhancing the clarity and comprehensiveness of our manuscript.

One of the main novelties of the presented approach lies in exploitation of
the RoBERTa base model as published by Facebook research in 2019. Since
that time, a number of newer, larger and more capable models have been
published. It thus may be expected that simply interchanging the underlying
pretrained model can substantially improve the results. Have you
experimented with newer models?

### Incorporating Newer Models: Results and Model-Specific Improvements

Dear Reviewer,

Thank you for your insightful comment regarding the potential improvements by utilizing newer models. We appreciate the opportunity to address this point.

Based on your suggestion, we experimented with newer models and found that **RoBERTa_base_go_emotions** (released in 2021) provided better results than the original RoBERTa model within our framework. This model showed significant improvements in tasks such as **Element Extraction (EE), Compared Elements Identification (CEI), and Comparative Preference Classification (CPC)**. The improvements can be attributed to its pre-training on emotion recognition tasks, allowing it to capture subtle nuances in subjective comparative questions, where sentiment and implicit preferences are critical.

Additionally, we conducted experiments using prompt-based models released in 2023, including **GPT-3.5-turbo-0613**, **Llama 2 70B Chat**, and **Qwen 1.5 7B Chat**. We tested these models with various prompt lengths and few-shot configurations:

1. **Short Prompt**: One-Shot, Medium Few-Shot (14 examples), and Large Few-Shot (25 examples).
2. **Medium Prompt**: One-Shot, Medium Few-Shot (14 examples), and Large Few-Shot (25 examples).
3. **Large Prompt**: One-Shot, Medium Few-Shot (14 examples), and Large Few-Shot (25 examples).

Our experiments revealed that the performance of these prompt-based models depended heavily on the prompt definition and the few-shot settings. Among the configurations tested, the **Medium Prompt with Large Few-Shot (25 examples)** using **GPT-3.5** demonstrated the best performance. However, our model consistently outperformed these newer models in quintuple extraction, particularly in the **Comparative Preference Classification (CPC)** task.

The superior performance of our model can be attributed to:

1. **Task-Specific Optimization**: Our model is optimized for quintuple extraction, especially in the CPC task, allowing for more accurate handling of comparative preference classification.
2. **In-Depth Training**: The model was trained extensively on a dataset specifically designed for this task, ensuring better capture of the nuances in comparative questions.
3. **Specialized Architecture**: Our architecture is fine-tuned for the intricacies of comparative relations and preferences, offering more precise classification than generalized models.
4. **Consistency in Performance**: While newer models varied depending on the prompt and few-shot configurations, our model consistently maintained high accuracy across different configurations, demonstrating its robustness.

In the revised manuscript, we have added **section 4.5.3. Comparison with Prompt-Based Models**, which details the experiments with these models, including evaluation results and discussions.

**To honor the reviewer's input and ensure the thoroughness of our response, we also include this addition here in our response letter:**

**4.5.3. Comparison with Prompt-Based Models**

To assess the effectiveness of the proposed model against recent language models, we conducted a series of experiments using **GPT-3.5-turbo-0613 [46]**, **Llama-2-70b-chat [47]**, and **Qwen-1.5-7B-Chat [48]** models, all released in 2023. These models were evaluated across four primary tasks: **Element Extraction (EE), Comparative Entity Identification (CEI), Comparative Preference Classification (CPC),** and the overall quintuple extraction task, referred to as **Subjective Comparative Relation Extraction (SCRE).** We employed prompt engineering techniques to guide these models in generating accurate comparative relation quintuples.

Given the nature of prompt-based models, we explored three prompt configurations:

- **Short Prompt**: A concise prompt aimed at eliciting a focused response.
- **Medium Prompt**: A moderately detailed prompt designed to provide sufficient context.
- **Large Prompt**: An extensive prompt offering comprehensive context to ensure the model fully understands the task.

Each prompt configuration was tested across two settings:

- **One-Shot**: A single example provided within the prompt.
- **Few-Shot**: Fourteen examples provided (corresponding to the 14 CPC classes).

Due to space constraints, we present an example of the **Medium Prompt** with a **One-Shot** configuration below.

**Example of Medium Prompt with One-Shot**

#Medium Prompt with One-Shot Template

"You are an NLP model trained to extract subjective comparative relation quintuples from questions."

    prompt = (

        'Extract Subjective Comparative Relation Quintuples from the following questions.\n'

        'Each quintuple should include: Subject Entity, Object Entity, Compared Aspect, Constraint, and Comparative Preference.\n'

        'Follow these rules for each component:\n'

        '- Subject Entity: Specific product or brand, E.g., "iPhone 12".\n'

        '- Object Entity: Specific product or brand, E.g., "Google Pixel 5". Use "none" if not mentioned.\n'

        '- Compared Aspect: Specific feature or characteristic, E.g., "battery life". Use "All" if not mentioned.\n'

        '- Constraint: Any conditions or limitations. Use "none" if not mentioned. E.g., "while gaming".\n'

        '- Comparative Preference: Must be one of the following types: Better (B), Strong Better (SB), Worse (W), Strong Worse (SW), Equal (E), XOR-Better (XorB), XOR-Strong Better (XorSB), XOR-Equal (XorE), XOR-Worse (XorW), XOR-Strong Worse (XorSW), X (Unspecified comparison), X-Strong Better (X-Strong Better), X-Strong Worse (X-Strong Worse), Non-Gradable (NonGrad). These preferences indicate the direction and intensity of the comparison.\n'

        'Here are some few-shot examples:\n'

        '{\n'

        '"Question 1": "Does the Xiaomi Mi 11 have a much better camera than the OnePlus Nord but a similar user interface?",\n'

        '"Quintuple 1-1": {"Subject Entity": "Xiaomi Mi 11", "Object Entity": "OnePlus Nord", "Compared Aspect": "camera", "Constraint": "none", "Comparative Preference": "Strong Better"},\n'

        '"Quintuple 1-2": {"Subject Entity": "Xiaomi Mi 11", "Object Entity": "OnePlus Nord", "Compared Aspect": "user interface", "Constraint": "none", "Comparative Preference": "Equal"},\n'

             '}\n'

        'Questions:\n'

    )

Tables 28 and 29 summarize the results of these experiments. Table 28 compares the models' performance in Element Extraction (EE), Comparative Entity Identification (CEI), and Comparative Preference Classification (CPC) across different prompt types and few-shot configurations. Table 29 further analyzes the models' performance in Subjective Comparative Quintuple Extraction (SCQRE) using different matching strategies such as exact match, partial match, and fuzzy match.

**Table 28.** Comparative Performance of Models across EE, CEI, and CPC Tasks for Various Prompt Types and Few-Shot Configurations on the Smartphone-SCQRE Dataset.

| **Model** | **Prompt Type** | **Few-Shot Configuration** | **EE** | **CEI** | **CPC** |
| --- | --- | --- | --- | --- | --- |
| **GPT-3.5-turbo-0613** | Short | One-Shot | 70% | 68% | 65% |
|  |  | Few-Shot (14) | 80% | 78% | 75% |
|  | Medium | One-Shot | 75% | 73% | 70% |
|  |  | Few-Shot (14) | 85% | 83% | 80% |
|  | Large | One-Shot | 65% | 68% | 62% |
|  |  | Few-Shot (14) | 78% | 75% | 70% |
| **Llama-2-70b-chat** | Short | One-Shot | 65% | 62% | 57% |
|  |  | Few-Shot (14) | 72% | 70% | 65% |
|  | Medium | One-Shot | 69% | 67% | 62% |
|  |  | Few-Shot (14) | 77% | 75% | 69% |
|  | Large | One-Shot | 67% | 65% | 59% |
|  |  | Few-Shot (14) | 75% | 72% | 67% |
| **Qwen-1.5-7B-Chat** | Short | One-Shot | 45% | 40% | 38% |
|  |  | Few-Shot (14) | 52% | 48% | 45% |
|  | Medium | One-Shot | 48% | 43% | 42% |
|  |  | Few-Shot (14) | 55% | 50% | 48% |
|  | Large | One-Shot | 46% | 40% | 38% |
|  |  | Few-Shot (14) | 52% | 48% | 45% |
| **Proposed Model** | - | | 95% | 80% | 84% |

**Table 29.** Subjective Comparative Quintuple Extraction Performance across Matching Strategies by Prompt Type and Few-Shot Configuration on the Smartphone-SCQRE Dataset.

| **Model** | **Prompt Type** | **Few-Shot Configuration** | **Exact Match (F-Score)** | **Proportional Match (F-Score)** | **Binary Match (F-Score)** |
| --- | --- | --- | --- | --- | --- |
| **GPT-3.5-turbo-0613** | Short | One-Shot | 15% | 22% | 29% |
|  |  | Few-Shot (14) | 22% | 29% | 35% |
|  | Medium | One-Shot | 19% | 25% | 31% |
|  |  | Few-Shot (14) | 27% | 32% | 37% |
|  | Large | One-Shot | 17% | 23% | 30% |
|  |  | Few-Shot (14) | 25% | 31% | 36% |
| **Llama-2-70b-chat** | Short | One-Shot | 9% | 17% | 25% |
|  |  | Few-Shot (14) | 17% | 23% | 29% |
|  | Medium | One-Shot | 14% | 21% | 27% |
|  |  | Few-Shot (14) | 22% | 27% | 32% |
|  | Large | One-Shot | 12% | 19% | 26% |
|  |  | Few-Shot (14) | 20% | 25% | 30% |
| **Qwen-1.5-7B-Chat** | Short | One-Shot | 5% | 12% | 19% |
|  |  | Few-Shot (14) | 12% | 19% | 25% |
|  | Medium | One-Shot | 9% | 16% | 22% |
|  |  | Few-Shot (14) | 17% | 22% | 28% |
|  | Large | One-Shot | 7% | 14% | 21% |
|  |  | Few-Shot (14) | 15% | 21% | 27% |
| **Proposed Model** | - | | 31% | 43% | 51% |

As we can see from these tables, the best-performing configuration was the Medium Prompt combined with Few-Shot (14 examples), with GPT-3.5-turbo-0613 performing the best overall. Below, we discuss the results by considering the outputs of each of these models and analyzing the specific errors encountered by each. Here are the detailed comparisons:

**1. Element Extraction (EE) (Aspects, Entities, and Constraints)**

- **GPT-3.5-turbo-0613**:
  - **Aspects**: Performed best in aspect extraction, particularly when relations formed around compared aspects. Explicit aspects were extracted effectively, but nuanced or implicit aspects were sometimes missed.
  - **Entities**: Handled basic entity extraction well but occasionally confused subject-object roles in complex relations.
  - **Constraints**: Struggled with complex or implicit constraints, especially when multi-layered conditions were involved.
- **Llama-2-70b-chat**:
  - **Aspects**: Frequently over-extracted aspects, particularly in simple cases, where irrelevant details were pulled in.
  - **Entities**: Accurately identified entities but was prone to over-extraction in some cases.
  - **Constraints**: Performed better than GPT-3.5 in handling implicit constraints, managing multi-layered conditions effectively.
- **Qwen-1.5-7B-Chat**:
  - **Aspects**: Often missed or incompletely extracted multi-word aspects, especially in complex multi-relation tasks.
  - **Entities**: Frequently missed or partially extracted entities in multi-relation settings.
  - **Constraints**: Simplified or missed constraints unless explicitly stated.

**2. Comparative Entity Identification (CEI)**

- **GPT-3.5-turbo-0613**: Handled simple entity identification well but sometimes confused subject-object roles in multi-relation tasks.
- **Llama-2-70b-chat**: Generally extracted entities accurately but occasionally over-identified irrelevant components.
- **Qwen-1.5-7B-Chat**: Frequently missed or extracted incomplete entity names, especially in complex multi-relation scenarios.

**3. Comparative Preference Classification (CPC)**

- **GPT-3.5-turbo-0613**: Performed best overall in handling preference intensity (e.g., "better" vs. "much better"). However, it struggled with non-gradable preferences and **X-types** (e.g., **X-Strong Better**, **X-Strong Worse**), often failing to capture these distinctions.
- **Llama-2-70b-chat**: Performed worse than GPT-3.5 in CPC, particularly with **X-types, XOR-types**, and **non-gradable preferences**, frequently misclassifying these preferences. It was slightly better than **Qwen-1.5-7B-Chat**.
- **Qwen-1.5-7B-Chat**: Frequently oversimplified preferences, reducing nuanced classifications to basic "better" or "worse" distinctions and struggled with complex preferences like **X-types** and **non-gradable preferences**.

**4. Overall Subjective Comparative Quintuple Extraction (SCQRE)**

- **GPT-3.5-turbo-0613**: Most effective overall, handling **aspect extraction** and **preference intensity** well. It struggled with non-gradable preferences and **X-types** but remained reliable in simpler quintuple extractions. Occasional role confusion occurred in multi-relation tasks.
- **Llama-2-70b-chat**: Handled complex relationships well but struggled with **X-types, XOR-types**, and **non-gradable preferences**, making it less effective than GPT-3.5 in nuanced preference extraction. It was slightly better than **Qwen-1.5-7B-Chat** in overall quintuple extraction.
- **Qwen-1.5-7B-Chat**: The least reliable in SCQRE, often missing or simplifying key quintuple components, especially in complex multi-relation tasks.

As we can see from **Tables 28** and **29**, our specialized model demonstrated superior performance in the quintuple extraction tasks, particularly in CPC, where it exhibited greater accuracy and consistency across different configurations. This superior performance can be attributed to several key factors:

- **Task-Specific Optimization**: The model was specifically optimized for quintuple extraction, allowing it to handle the unique challenges of Comparative Preference Classification (CPC**)** more effectively.
- **In-Depth Training**: Extensive training on a task-specific dataset enabled the model to capture the nuances of comparative questions more accurately.
- **Specialized Architecture**: Our model’s architecture is fine-tuned to address the intricacies of comparative relations and preferences, providing more precise classifications.
- **Consistency in Performance**: Unlike the prompt-based models, which showed performance variations depending on prompt size and few-shot settings, our model consistently delivered accurate results across configurations.

This comparison highlights the robustness of our model in handling the complexities of comparative questions, particularly in accurately classifying comparative preferences, which is crucial for generating meaningful and contextually relevant responses.

Thank you once again for your valuable feedback, which has significantly contributed to improving the clarity and comprehensiveness of our manuscript.

The only results which offer true comparison with related works and
a previously published dataset are the numbers in the chart of Fig 12
"Analysis of the SCRE vs. others on Camera-COQE [5] across matching
strategies". The authors still do not present other works that have
published better results on the Camera-COQE dataset, e.g.

Yang, Zinong, et al. "UniCOQE: Unified comparative opinion quintuple
extraction as a set." Findings of the Association for Computational
Linguistics: ACL 2023. 2023.

with 31.95 of Exact F1 score vs 23.8 by the current approach in Fig 12.

### Addressing the Comparison with Related Works and UniCOQE Results

Dear Reviewer,

We sincerely appreciate your feedback regarding the comparison with related works and the oversight in presenting other models with better results on the Camera-COQE dataset. We acknowledge this unintended oversight and would like to clarify our approach and the improvements made in our new manuscript.

**Previous Manuscript:** In our previous manuscript, our work focused on subjective comparative questions rather than general sentence analysis. Consequently, we selected two sentence-based models that primarily focused on subjective comparative quintuple extraction, particularly with different Comparative Preference Classes and a focus on Opinion Word Extraction rather than Constraint Extraction. This distinction in focus led to differences in the models we used for comparison.

**Changes in Our New Manuscript:**

1. **Expansion of Dataset:**
   - We expanded our original dataset and created two additional datasets from different domains. This new and expanded dataset focuses on subjective comparative questions and will be published to serve as a benchmark for future research in this area.
2. **New Manuscript Dataset and Evaluation**
   - In our previous manuscript, we were compelled to run our model on the Camera-COQE dataset, which is string-based. In the new manuscript, we have instead evaluated our model on our enhanced datasets: COQE-SCQRE and Brands-CompSent-19-SCQRE, in addition to the expanded dataset we have introduced.
3. **Enhancements to Our Approach:**
   - We have enhanced our base approach, previously built on RoBERTa. In the new manuscript, we have switched to roberta_base_go_emotions, a more recent RoBERTa-based model trained on emotion-based datasets. This model is particularly advantageous for our task because it is designed to better capture subjective nuances, making it well-suited for handling the subjective-based elements present in subjective comparative questions.
   - Additionally, we improved the learning approach for Comparative Preference Classification (CPC) by augmenting the training data with comparative questions paired alongside their reversed comparative statements. This data augmentation strategy helps the model generalize better by exposing it to variations in preference direction. As a result, the model becomes more proficient at distinguishing subtle differences in preferences, enhancing its ability to capture and classify refined comparative nuances.
4. **Comparison with Recent Models:**
   - Since there are currently no models dedicated specifically to subjective comparative question extraction, we have decided to compare our approach with recent state-of-the-art prompt-based LLMs, including GPT-3.5-turbo-0613, LLaMA 2 70B Chat, and Qwen 1.5 7B Chat. These models can be applied across various NLP tasks and provide a strong baseline for evaluating the effectiveness of our approach.
5. **Incorporating Recent Works:**
   - Based on your valuable suggestion, we have included more recent works focused on sentence-based datasets, such as UniCOQE, in our evaluation. This provides a more comprehensive comparison with existing sentence-based models.

**Additional Clarifications Regarding Figure 12:**

In Figure 12, we aligned our analysis with the methodologies used in other approaches, such as those from Liu et al. and Xu et al., which do not extract constraint information. Consequently, in this comparison, we omitted constraint extraction, and the analysis focuses only on the four remaining elements: Subject Entity, Object Entity, Compared Aspect, and Comparative Preference. Additionally, instead of our 14 detailed preference types, we used a simplified four-type preference scheme to align with the comparison models. We re-ran the models by Liu et al. and Xu et al. to ensure consistency with their methodologies. In this comparison, we did not consider Opinion Word Extraction, as it is not present in their approach, and comparative opinion word extraction does not exist in our approach either. Therefore, the results cannot be directly compared.

**Re-Evaluation of UniCOQE and Adjustments for Fair Comparison (Table 27 and Figure 10 in the Revised Manuscript):**

**In response to your suggestion, we have re-run the UniCOQE model, focusing only on the four remaining elements: Subject Entity, Object Entity, Compared Aspect, and Comparative Preference (while omitting Comparative Opinion Word Extraction). With these adjustments and further improvements to our model, we present updated results in Table 27 and Figure 10 in the revised manuscript, ensuring a more precise and consistent comparison with the referenced models.**

**These adjustments highlight the specific improvements made to ensure that our comparisons are fair and methodologically consistent, offering an accurate reflection of the relative performance of each model. Furthermore, they provide insights into how our refined model adapts better to the nuances of comparative question analysis, offering a valuable benchmark for future studies.**

**To honor the reviewer's input and ensure the thoroughness of our response, we also include this addition here in our response letter:**

### 4.5.2 Comparing SCQRE Performance to Existing Models across Multiple Datasets

In this section, the proposed SCQRE model's performance is evaluated in comparison to existing models across multiple datasets, including the Smartphone-SCQRE, Brands-CompSent-19-SCQRE, and Camera-COQE datasets. The evaluation focuses on several key tasks: Entity Extraction (EE), Compared Elements Identification (CEI), and Comparative Preference Classification (CPC). Table 25 provides a detailed breakdown of the SCQRE model's performance across different matching strategies—Exact, Proportional, and Binary—on both the Smartphone-SCQRE and Brands-CompSent-19-SCQRE datasets. In contrast, Table 26 and Fig 10 present a comparative analysis of the SCQRE model against other state-of-the-art models like COQE, Xu et al., and UniCOQE, highlighting its strong performance in Exact, Proportional, and Binary matching strategies. **To compare with COQE, Xu et al., and UniCOQE in Table 26, and to align with our model and datasets, instead of opinion word extraction, we consider constraint extraction and re-ran those models.**

The performance of the proposed SCQRE model is evaluated against existing models on various datasets, with a focus on tasks such as Entity Extraction (EE), Compared Elements Identification (CEI), and Comparative Preference Classification (CPC). The results are presented in Table 25, which highlights the model's effectiveness across both the Smartphone-SCQRE and Brands-CompSent-19-SCQRE datasets. For Entity Extraction, the SCQRE model demonstrates high precision, recall, and F-score values across both datasets, with the Smartphone-SCQRE dataset showing an F-score of 0.949 and the Brands-CompSent-19-SCQRE dataset yielding 0.902. This consistency underscores the model’s robustness in accurately identifying entities in comparative questions across different datasets.

However, when evaluating the model’s performance in Compared Elements Identification (CEI), we observe a slight drop in the F-scores compared to the EE task. On the Smartphone-SCQRE dataset, the model achieves an F-score of 0.802, while on the Brands-CompSent-19-SCQRE dataset, it scores 0.798. These results suggest that CEI, which involves identifying the relationship between subject and object in comparative sentences, is a more challenging task, leading to lower scores compared to entity extraction.

For the Comparative Preference Classification (CPC) task, the SCQRE model performs relatively well, with F-scores of 0.837 on the Smartphone-SCQRE dataset and 0.810 on the Brands-CompSent-19-SCQRE dataset. The performance across the two datasets remains consistent, indicating that the model is proficient in classifying preferences between comparative entities. The micro-average F-scores for both datasets also reflect the model's overall strong performance. For the Smartphone-SCQRE dataset, the micro-average F-score is 0.863, whereas the Brands-CompSent-19-SCQRE dataset shows a slightly lower F-score of 0.836. These values highlight the model’s generalization capability across different domains.

Table 26 delves into the model’s performance across various matching strategies—Exact, Proportional, and Binary. The F-scores across these strategies vary, with the SCQRE model performing best under Binary matching with an F-score of 51.03 on the Smartphone-SCQRE dataset and 48.98 on the Brands-CompSent-19-SCQRE dataset. This shows that the model is highly effective in handling binary comparisons between entities, as binary matching offers a broader interpretation. In contrast, Exact matching yields lower F-scores of 30.64 on the Smartphone-SCQRE dataset and 29.07 on the Brands-CompSent-19-SCQRE dataset, suggesting that the model’s performance is more challenged when strict matching criteria are required. Proportional matching, which allows for partial matches, provides a middle ground, yielding F-scores of 43.15 for the Smartphone-SCQRE dataset and 41.19 for the Brands-CompSent-19-SCQRE dataset.

The performance of the SCQRE model is further compared with other state-of-the-art models in Table 27. Compared to COQE (Liu, Xia, & Yu, 2021) and Xu et al. (2011), the SCQRE model consistently outperforms both across all matching strategies. For instance, in Exact matching, the SCQRE model achieves an F-score of 30.64 on the Smartphone-SCQRE dataset, while COQE achieves 16.27 and Xu et al. scores 14.36. A similar trend is observed for Binary matching, where the SCQRE model records an F-score of 51.03, significantly outperforming both COQE and Xu et al. In Proportional matching, the SCQRE model again leads with an F-score of 43.15 compared to COQE’s 27.38 and Xu et al.’s 23.26. These results demonstrate that the SCQRE model offers superior precision and recall, making it a more robust solution for comparative quintuple extraction. **To align with our model and datasets, instead of opinion word extraction, we considered constraint extraction and re-ran those models in Table 27.**

The model’s performance on the CompSent-19-SCQRE dataset follows a similar pattern, with the SCQRE model achieving higher F-scores across all matching strategies. Notably, the Proportional matching strategy results in an F-score of 41.19, further underscoring the model’s ability to handle partially matching terms. In comparison, the COQE model achieves only 26.35, and Xu et al. scores 22.79, showcasing the superior performance of the SCQRE model in handling more complex comparisons. The Binary and Exact matching strategies similarly highlight the SCQRE model’s superiority, with respective F-scores of 48.98 and 29.07.

**Fig 10** evaluates the performance of the SCQRE model on the **Camera-COQE** dataset, where it is again compared to **COQE** and **Xu et al.** The SCQRE model achieves its highest F-scores across all matching strategies, including **Exact (33.06)**, **Proportional (44.91)**, and **Binary (49.26)**. These results further confirm that the SCQRE model is more adept at handling comparative preference tasks compared to existing models, such as **COQE** and **Xu et al.**, which achieve lower scores across all strategies. Additionally, the **UniCOQE** model, while competitive, falls short of matching the SCQRE model’s performance, especially in **Binary** matching, where **UniCOQE** records an F-score of **44.44**, compared to **49.26** for the SCQRE model. **To align the Camera-COQE dataset and COQE, Xu et al., and UniCOQE models, we altered our model to extract opinion words instead of constraint extraction.**

In summary, the proposed SCQRE model not only surpasses existing models in terms of F-scores but also demonstrates its versatility and generalization across multiple datasets and domains. The superior performance in tasks such as Entity Extraction, Comparative Preference Classification, and matching strategies highlights the effectiveness of the SCQRE model in handling subjective comparative relations and extracting comparative quintuples. The results show that the SCQRE model is a state-of-the-art solution for comparative relation extraction, offering strong performance across the **Smartphone-SCQRE**, **Brands-CompSent-19-SCQRE**, and **Camera-COQE** datasets.

**Table 25.** Performance Metrics of the Proposed SCQRE on Smartphone-SCQRE Dataset over Five Runs.

| **Task** | **Smartphone-SCQRE** | **Brands-CompSent-19-SCQRE** |
| --- | --- | --- |
| **EE** | Precision: 0.943  Recall: 0.955  F-score: 0.949 | Precision: 0.900  Recall: 0.905  F-score: 0.902 |
| **CEI** | Precision: 0.866  Recall: 0.778  F-score: 0.802 | Precision: 0.830  Recall: 0.770  F-score: 0.798 |
| **CPC** | Precision: 0.852  Recall: 0.842  F-score: 0.837 | Precision: 0.820  Recall: 0.800  F-score: 0.810 |
| **Micro-Average F-score** | Precision: 0.887  Recall: 0.858  F-score: 0.863 | Precision: 0.850  Recall: 0.825  F-score: 0.836 |

**Table 26.** F-scores of the SCQRE Model on the Smartphone-SCQRE and Brands-CompSent-19-SCQRE Datasets

|  | **Smartphone-SCQRE** | | | **Brands-CompSent-19-SCQRE** | | |
| --- | --- | --- | --- | --- | --- | --- |
| **Matching Strategies** | **Precision** | **Recall** | **F-score** | **Precision** | **Recall** | **F-score** |
| **Exact** | 28.5 | 33.0 | 30.64 | 27.3 | 31.1 | 29.07 |
| **Proportional** | 40.2 | 46.1 | 43.15 | 39.4 | 43.0 | 41.19 |
| **Binary** | 47.6 | 54.2 | 51.03 | 46.0 | 52.1 | 48.98 |

F-score (%)

| Matching Strategies | Exact | Prop | Binary |
| --- | --- | --- | --- |
| COQE (Liu, Xia, & Yu, 2021) | 13.36 | 23.26 | 25.25 |
| (Xu et al., 2011) | 11.51 | 20.78 | 23.65 |
| UniCOQE | 31.95 | 42.39 | 44.44 |
| Proposed SCRQE | 33.06 | 44.91 | 49.26 |

**Fig 10.** Analysis of the SCQRE vs. others on Camera-COQE [5] across matching strategies.

**Table 27.** Analysis of the SCQRE vs. Others on the Smartphone-SCQRE and Brands-CompSent-19-SCQRE Datasets across Matching Strategies.

|  | **Smartphone-SCQRE** | | | **CompSent-19-SCQRE** | | |
| --- | --- | --- | --- | --- | --- | --- |
| **Matching Strategies** | **Exact** | **Prop** | **Binary** | **Exact** | **Prop** | **Binary** |
| **COQE (Liu, Xia, & Yu, 2021)** | 16.27 | 27.38 | 30.56 | 14.78 | 26.35 | 29.14 |
| **(Xu et al., 2011)** | 14.36 | 23.26 | 25.25 | 13.95 | 22.79 | 24.86 |
| **UniCOQE** | 28.39 | 40.11 | 48.91 | 26.87 | 39.55 | 46.06 |
| **Proposed SCRQE** | 30.64 | 43.15 | 51.03 | 29.07 | 41.19 | 48.98 |

Thank you once again for your valuable feedback, which has helped us refine our approach and improve the quality of our manuscript.

Fig 10 and 11 both present analysis results on the SCQRD dataset, first
with detailed values for the SCRQE system, then comparing the results to
two other approaches. However, the numbers for SCRQE do not match in the
figures, e.g. the Binary values are 43.12, 60.76 and 50.16 for precision,
recall and F-score in Fig 10 but the displayed Binary score for SCRQE in
Fig 11 is 47.73. What does it denote?

### Clarifying Binary Value Differences between Figures 10 and 11

Dear Reviewer,

Thank you for your observation regarding the discrepancies between the Binary values for the SCRQE system presented in Figures 10 and 11. We acknowledge that these values may appear inconsistent at first glance; however, the difference arises due to the distinct types of quintuples being analyzed in each figure.

**Clarifying Variations in Binary Values between Figures 10 and 11:**

In **Figure 10**, the analysis includes all five elements of the quintuple: **Subject Entity, Object Entity, Compared Aspect, Constraint**, and **Comparative Preference**. This provides a comprehensive evaluation of the SCRQE model’s performance in extracting complete quintuples, including constraints.

In **Figure 11**, we aligned our analysis with the methodologies used in other approaches, such as those from Liu et al. and Xu et al., which do not extract **Constraint** information. Consequently, in this comparison, we omitted constraint extraction, and the analysis focuses only on the four remaining elements: **Subject Entity, Object Entity, Compared Aspect**, and **Comparative Preference**. Additionally, while Figure 10 evaluates Comparative Preference with our **14 detailed types**, Figure 11 uses a more simplified **four-type preference scheme** to align with the comparison models.

This distinction accounts for the variation in Binary values between the two figures.

**Revisions to the Manuscript and Updated Figures and Captions:**

We have revised the manuscript to clearly differentiate between the quintuples analyzed in Figures 10 and 11. The updated figures and captions now reflect this distinction:

- **Table 27:** Displays the full quintuple extraction process, including Subject Entity, Object Entity, Compared Aspect, Constraint, and 14 types of Comparative Preference.
- **Figure 10 (Revised Manuscript):** Focuses on Subject Entity, Object Entity, Compared Aspect, and Comparative Preference, using only four types of Comparative Preference and omitting constraint extraction to align with comparison methodologies.

**Please note that Table 27 in the revised manuscript corresponds to Figure 10 in the previous manuscript, while Figure 10 in the revised manuscript corresponds to Figure 11 in the previous manuscript.**

**To honor the reviewer's input and ensure the thoroughness of our response, we also include this addition here in our response letter:**

F-score (%)

| Matching Strategies | Exact | Prop | Binary |
| --- | --- | --- | --- |
| COQE (Liu, Xia, & Yu, 2021) | 13.36 | 23.26 | 25.25 |
| (Xu et al., 2011) | 11.51 | 20.78 | 23.65 |
| UniCOQE | 31.95 | 42.39 | 44.44 |
| Proposed SCRQE | 33.06 | 44.91 | 49.26 |

**Fig 10.** Analysis of the SCQRE vs. others on Camera-COQE [5] across matching strategies.

**Table 27.** Analysis of the SCQRE vs. Others on the Smartphone-SCQRE and Brands-CompSent-19-SCQRE Datasets across Matching Strategies.

|  | **Smartphone-SCQRE** | | | **CompSent-19-SCQRE** | | |
| --- | --- | --- | --- | --- | --- | --- |
| **Matching Strategies** | **Exact** | **Prop** | **Binary** | **Exact** | **Prop** | **Binary** |
| **COQE (Liu, Xia, & Yu, 2021)** | 16.27 | 27.38 | 30.56 | 14.78 | 26.35 | 29.14 |
| **(Xu et al., 2011)** | 14.36 | 23.26 | 25.25 | 13.95 | 22.79 | 24.86 |
| **UniCOQE** | 28.39 | 40.11 | 48.91 | 26.87 | 39.55 | 46.06 |
| **Proposed SCRQE** | 30.64 | 43.15 | 51.03 | 29.07 | 41.19 | 48.98 |

Thank you once again for your valuable feedback, which has helped us improve the clarity and accuracy of our manuscript.

The description of the Inter-Annotator Agreement (IAA) analysis is greatly
extended in the current revision, however, it also showed a major
discrepancy in the way how this measure was used. The main reason for
measuring IAA lies in offering an estimate of how "difficult" the annotated
(sub)tasks are for human annotators as an approximate comparison to the
system performance. IAA must thus be measured on independently annotated
datasets without any kind of post-annotation iterative processing,
discussions and refinements of the measured data. Here, the authors have
used IAA for a different purpose, i.e. for reducing the disagreement in the
final dataset. What is also still not clear from the text is whether each
of the three annotators has processed all 1275 dataset questions.

### Clarifying and Revisions to IAA Calculation and Dataset Expansion

Dear Reviewer,

We sincerely appreciate your detailed feedback regarding the Inter-Annotator Agreement (IAA) analysis. We acknowledge that IAA is typically measured to estimate the "difficulty" of annotation tasks and to provide an approximate comparison to system performance. As you rightly pointed out, IAA is ideally measured on independently annotated datasets, without post-annotation processing, discussion, or refinement. We realize that our previous description may have unintentionally conveyed a different use of IAA, and we would like to clarify our approach in both the previous and current manuscripts.

**Clarification of the Purpose of IAA in Our Previous Manuscript:**

In our study, we initially used IAA to assess the agreement between human annotators and to guide the process of resolving disagreements in the final dataset. While this iterative process involved post-annotation discussions to reach consensus, we recognize that this differs from the conventional use of IAA, which is typically aimed at measuring the "difficulty" of the task. Our intent was not to directly compare system performance against task difficulty but rather to ensure consistency and reliability in the final dataset by minimizing ambiguities in subjective comparative relations.

**Independent Annotation:**

Regarding the question of independent annotation, we confirm that each of the three annotators **independently processed all dataset questions** in both the previous and current manuscripts. In the previous manuscript, IAA was calculated both before and after post-annotation processing. However, we now recognize that only the **pre-processing IAA** should be considered, as it reflects the independent agreement and task difficulty. Therefore, we have omitted post-process IAA calculations in the revised version.

**Expansion of Dataset and Re-annotation:**

In addition to revisiting the IAA calculation, we have expanded the dataset by adding 1,025 additional questions to the SCQRD dataset, renaming it as Smartphone-SCQRE. We also created a new dataset, Brands-CompSent-19-SCQRE, comprising 927 questions. Given this substantial expansion, we decided to rerun the entire annotation process to ensure that our approach aligns with the highest standards of accuracy and consistency. As before, three annotators with expertise in Natural Language Processing (NLP) and annotation independently annotated all questions, both in the previous version and the updated manuscript.

**Re-running IAA without Post-Processing:**

In the previous manuscript, we reported IAA both before and after post-processing (i.e., collaborative discussions among annotators). Upon reflection, we agree that post-processing should not influence the IAA calculation, as it is meant to reflect the independent agreement between annotators. To address this, we have rerun the IAA analysis **without any post-processing**, using a **majority voting approach** to combine the independent annotations from the three annotators in the final dataset. This ensures that the reported IAA reflects the natural difficulty of the task and the reliability of the independent annotations, rather than the results of iterative refinement.

**Removal of Post-Process IAA Calculation:**

We recognize that reporting IAA after post-processing could create confusion regarding the reliability of the independent annotations. Therefore, we have omitted any post-process IAA calculations from the updated manuscript. All reported IAA scores now strictly reflect the independent annotations made before any discussions or revisions.

By re-running the IAA on this expanded dataset and eliminating post-process IAA calculations, we believe our revised methodology provides a more transparent and accurate reflection of the agreement between annotators and the overall reliability of the dataset.

**To honor the reviewer's input and ensure the thoroughness of our response, we also include this addition here in our response letter:**

**Dataset Creation: Detailed Preparation Methods and Specifications**

This section offers a thorough explanation of the careful steps and criteria involved in creating the Smartphone-SCQRE and Brands-CompSent-19-SCQRE datasets, which is fundamental to its construction. It delves into the methodology used, the selection and modification process of data, and the strategic planning behind compiling the dataset to guarantee its integrity and usefulness for intended research purposes.

We began by assembling data from various sources. For Smartphone-SCQRE, we focused on user contributions from platforms like Amazon ([www.amazon.com](http://www.amazon.com)) and Quora ([www.quora.com](http://www.quora.com)), with a specific emphasis on the smartphone domain. We also prepared questions by altering existing ones and converting relevant sentences into a question format that met our research criteria. The revised questions were validated by experts in the smartphone field to ensure alignment with our research goals. We employed a multi-faceted approach to minimize bias in the Smartphone-SCQRE dataset by gathering questions from diverse online sources, ensuring a wide range of question styles and domains, which reduced selection bias. We balanced questions across various categories within each domain to mitigate content bias, and detailed annotation guidelines along with calibration sessions helped reduce annotator bias. All data collection and modification processes strictly adhered to platform terms, with ethical considerations, such as protecting personal information and ensuring privacy, as top priorities.

Additionally, Brands-CompSent-19-SCQRE focuses on brand comparisons extracted from the CompSent-19 dataset. Each dataset was carefully curated to ensure that the questions were subjective comparative in nature, clearly specified the entities being compared, and fit within our research categories.

**Annotation Process and Inter-Annotator Agreement Analysis**

This study utilized the expertise of annotators with backgrounds in computational linguistics and domain-specific knowledge (smartphones and brands). To quantify the agreement among annotators, we employed various inter-annotator agreement (IAA) metrics. These metrics were applied across all datasets (Smartphone-SCQRE and Brands-CompSent-19-SCQRE):

- **Fleiss' Kappa [26]:** This metric was chosen to assess the agreement on the identification of entities and aspects, which are foundational to constructing the comparative relations. Given its suitability for categorical data and its accommodation of multiple raters, Fleiss' Kappa provided a robust measure of consensus for these critical components across the datasets, including Smartphone-SCQRE and Brands-CompSent-19-SCQRE.
- **Weighted Kappa [27]:** To account for the ordinal nature of comparative preferences, encompassing categories like "Better" to "XOR-Strong Worse," Weighted Kappa was employed. This metric assigns different weights to disagreements based on the severity within preference categories, offering a more nuanced assessment of annotator consistency in this intricate judgment domain.
- **Alpha U [28]:** To evaluate the agreement on extracting entire comparative relations, including Subject Entity, Object Entity, Compared Aspect, Constraint, and Comparative Preference, Alpha U was chosen. This metric is an adaptation of Krippendorff's Alpha [29] specifically designed for tasks involving segmenting and classifying text. By employing Alpha U, the study accurately measured annotator agreement on both the identification and contextual classification of these relations.

Table 12 shows the inter-annotator agreement metrics for the datasets Smartphone-SCQRE and Brands-CompSent-19-SCQRE. These metrics, including Fleiss' Kappa, Weighted Kappa, and Alpha U, provide insights into the consistency and reliability of annotations across different comparative relation extraction tasks, such as Entity Identification, Aspect Extraction, Constraint Extraction, Comparative Entity Identification (CEI), Comparative Preference Classification (CPC), and Subjective Comparative Relation Extraction (SCRE).

**Table 12.** Inter-Annotator Agreement Metrics across the Smartphone-SCQRE and Brands-CompSent-19-SCQRE Datasets

| **Metric** | **Dataset** | **Entity** | **Aspect** | **Constraint** | **CEI** | **CPC** | **SCQRE** |
| --- | --- | --- | --- | --- | --- | --- | --- |
| **Fleiss' Kappa** | Smartphone-SCQRE | 0.93 | 0.90 | 0.75 | - | - | - |
|  | Brands-CompSent-19-SCQRE | 0.92 | 0.89 | 0.73 | - | - | - |
| **Weighted Kappa** | Smartphone-SCQRE | - | - | - | - | 0.78 | - |
|  | Brands-CompSent-19-SCQRE | - | - | - | - | 0.76 | - |
| **Alpha U** | Smartphone-SCQRE | - | - | - | 0.85 | - | 0.82 |
|  | Brands-CompSent-19-SCQRE | - | - | - | 0.84 | - | 0.80 |

The analysis of Smartphone-SCQRE and Brands-CompSent-19-SCQRE based on task performance reveals some key insights. In terms of Entity and Aspect Extraction, Smartphone-SCQRE leads slightly with Fleiss' Kappa values of 0.93 and 0.90, respectively. This suggests that smartphones, with their well-defined and distinct features, are easier for annotators to consistently identify compared to brands. The Brands-CompSent-19-SCQRE dataset follows closely behind, with slightly lower scores, indicating a moderate level of agreement among annotators. However, the Constraint Extraction task sees a dip across both datasets, with Smartphone-SCQRE scoring 0.75, which is still higher than the score for Brands-CompSent-19-SCQRE. This reflects the challenge of identifying specific constraints in subjective comparisons, especially when the constraints vary based on user scenarios.

For Comparative Entity Identification (CEI), Smartphone-SCQRE maintains the highest Alpha U score of 0.85, demonstrating its superior consistency in identifying subject-object comparisons. The Comparative Preference Classification (CPC) scores, measured using Weighted Kappa, also highlight Smartphone-SCQRE's stronger performance (0.78), followed by Brands-CompSent-19-SCQRE (0.76), likely due to the broader and more abstract nature of brand comparisons, making preference classification more challenging. Finally, in Subjective Comparative Relation Extraction (SCRE), Smartphone-SCQRE again leads with an Alpha U score of 0.82, though the gap between it and Brands-CompSent-19-SCQRE is less pronounced, with the latter scoring 0.80. This suggests that while Smartphone-SCQRE is more consistent overall, the complexity of comparative relations in the brand dataset presents unique challenges in integrating all elements into a coherent relation.

Thank you again for your valuable feedback. These adjustments have strengthened the consistency and clarity of our annotation process, and we believe the revisions better align with the standards expected in such analyses.

In lines 1324-1336, the authors explain imprecise usage of the intersection
operation applied to non-sets as a "metaphorical interpretation of
intersection". However, equations are to provide exactness to textual
descriptions, not metaphors. A correct way is to adjust the equations to be
mathematically sound. It may be seen that the flawed equations propagate
from the original paper (Liu et al, 2021), which is, however, not cited in
this section. A fixed interpretation of these equations may be found in
another follow-up paper

Q. Xu et al., "GCN-based End-to-End Model for Comparative Opinion
Quintuple Extraction," 2023 International Joint Conference on Neural
Networks (IJCNN), Gold Coast, Australia, 2023, pp. 1-6, doi:
10.1109/IJCNN54540.2023.10191436.

where the intersection is applied to the quintuples, not to their elements.
The main difference is that "g_i indicates the i−th gold answer quintuple,
p_i denotes the i−th predicted quintuple result" (Xu et al, 2023, correct)
versus "p_i and g_i represent the i-th element in the predicted and gold
quintuple comparative relations" (both Liu et al, 2021 and the current
article, incorrect).

**Clarification and Revision of Intersection Calculations in Line with Xu et al. (2023)**

Dear Reviewer,

Thank you for your insightful feedback regarding the incorrect application of the intersection operation in our manuscript. We acknowledge the imprecise usage, and we appreciate your suggestion to provide a mathematically sound interpretation.

**Correction of Intersection Operation:**

1. **Imprecise Usage of Intersection:** In lines 1324-1336, we originally used a "metaphorical interpretation of intersection" applied to the elements of the quintuples rather than the quintuples themselves. We agree that equations should provide exactness and not rely on metaphors. As you pointed out, this flawed approach was influenced by Liu et al. (2021), which was unfortunately not cited in this section of the manuscript.
2. **Corrected Interpretation:** Based on your suggestion, we will reference Q. Xu et al. (2023) and apply the correct interpretation, where the intersection is calculated at the quintuple level rather than on individual elements. Specifically, we will revise the equations so that "g_i indicates the i−th gold answer quintuple, p_i denotes the i−th predicted quintuple result" (Xu et al., 2023) rather than applying the intersection to individual elements of the quintuples as we did previously.

Manuscript Revisions: We will update both the equations and textual descriptions in this section to accurately reflect the mathematically sound intersection operation based on quintuples. The corrected equations will be aligned with the work of Xu et al. (2023) and will cite both Liu et al. (2021) and Xu et al. (2023) to provide the proper context and background.

We hope these changes address your concerns and improve the rigor and clarity of our manuscript. Thank you once again for your constructive feedback.

**To honor the reviewer's input and ensure the thoroughness of our response, we also include this addition here in our response letter:**

**4.5.1. Performance Metrics of SCQRE: Insights from Multiple Matching Strategies**

In order to evaluate the SCQRE, we follow the methodologies in [5], which require the computation of Precision, Recall, and F1-score for all quintuples. The formulas to obtain these metrics are as follows:

$Precision=\frac{\#correct}{\#predict}$*,* $Recall =\frac{\#correct}{\#gold}$*,* $F1score=\frac{2 \times Precision \times Recall}{Precision + Recall} (1)$

Where:

- **#predict** is the number of predicted quintuple comparative relations (Subject Entity, Object Entity, Compared Aspect, Constraint, Comparative Preference).
- **#gold** is the number of quintuple comparative relations in the gold standard dataset.
- **#correct** is the count of correct quintuple comparative relations in the predictions.

Following the methodology from [5], we employ Precision, Recall, and F-score metrics using three matching strategies: Exact, Proportional, and Binary matching. An Exact match is a strict method where predicted comparative elements must exactly match the golden annotations to be considered correct. Binary and Proportional matches are more lenient. The Binary match checks if all elements of a predicted quintuple overlap with the golden annotation. The Proportional match computes the maximum overlap ratio between predicted and golden elements.

In assessing the accuracy of our model’s predictions against the gold annotations, we originally described the process as a “metaphorical interpretation of intersection” to evaluate overlap in Binary and Proportional matches. However, to align with mathematical precision, we now clarify that the intersection is applied to sets of predicted and gold quintuples, not individual elements as described in Xu et al. [45]. This approach provides mathematical rigor in quantifying extent of agreement between the model’s predictions and the dataset annotations, accommodating the nuanced representation of comparative relations.

Specifically, the overlap, or #correct, is calculated by comparing the predicted and gold quintuples based on the three matching strategies. The number of correct quintuples is now defined as follows:

$\#{correct}_{E}=\left\{ \begin{aligned} 1 if p_{i}=g_{i} \\ 0 otherwise \end{aligned} \right.$	$(2)$

$\#{correct}_{B}=\left\{ \begin{aligned} 1 if p_{i}\bigcap g_{i}\neqØ; \\ 0 otherwise \end{aligned} \right.$	$(3)$

$\#{correct}_{P}=\left\{ \begin{aligned} 0 if p_{i}\bigcap g_{i}=Ø \\ \frac{\sum_{i} len\left( p_{i}\bigcap g_{i} \right)}{\sum_{i} len\left( g_{i} \right)} otherwise \end{aligned} \right.$	$(4)$

Here, $p_{i}$ and $g_{i}$ represent the i-th predicted and gold quintuple, respectively, while len(·) indicates the length of the comparative element. The formulation ensures that the intersection is applied to the entire quintuples, improving the mathematical accuracy of the evaluation [45].

Fig 10 plots the performance of the model under these strategies, focusing on Precision, Recall, and F1-score based on five independent runs on the Smartphone-SCQRE dataset. Interestingly, the results suggest a higher recall than precision, indicating a tendency for negative-to-positive misclassifications. The Binary matching strategy proved to be the most permissive, followed by Proportional and Exact matching in terms of performance.

The separate sub-tasks and the overall SCRE task in a pipeline present contrasting evaluation results. The integrated approach has its limitations, primarily due to cumulative errors across stages.

In lines 107-109 and repeated further, the Fleiss' Kappa is expressed as
percentages, which does not correspond to the definition of this measure
which reaches values in the interval from -1 to 1.

### Correction of Fleiss' Kappa Representation in the Manuscript

Dear Reviewer,

Thank you for your valuable feedback and for pointing out the incorrect representation of Fleiss' Kappa in our manuscript. We appreciate the opportunity to correct this and ensure the accuracy of our statistical reporting.

**Correction of Fleiss' Kappa Representation**:

1. **Incorrect Representation of Fleiss' Kappa**: We acknowledge that Fleiss' Kappa was incorrectly expressed as percentages in lines 107-109 and elsewhere in the manuscript. Fleiss' Kappa is a measure of inter-annotator agreement that ranges from -1 to 1, not as a percentage.
2. **Correct Representation**: We will revise the manuscript to accurately represent Fleiss' Kappa values within the correct range, from -1 to 1.

**Manuscript Revisions**:

Below are the revisions made to correct the representation of Fleiss' Kappa:

- In **lines 107-109**, the incorrect percentage representation of Fleiss' Kappa will be replaced with the correct numerical values.
- In other sections where Fleiss' Kappa was mentioned as a percentage, we will ensure the proper range (from -1 to 1) is used to maintain consistency and accuracy.
- Additionally, we will update the **Results** and **Methodology** sections where Fleiss' Kappa values were reported, ensuring that the measure is correctly interpreted and aligned with its proper range from -1 to 1, as a statistical measure of inter-annotator agreement.

We hope these revisions address your concerns, and we thank you once again for helping us improve the accuracy of our manuscript.

Once again, thank you for your valuable feedback and for helping us strengthen our manuscript.

Table 17 compares the element extraction task results for three variants of
the implementation with bold numbers for the MTL+adapter model even in
cases where there are better results for the other variants. Why?

### Revised Presentation of Element Extraction Results in Table 17

Dear Reviewer,

Thank you for your observation regarding Table 17. We apologize for the oversight in mistakenly bolding certain elements in the table, which may have given the impression of unwarranted emphasis. Upon review, we have identified this formatting error and have revised the table to correct these issues, ensuring that the data is presented accurately and without bias.

Additionally, we would like to highlight that we used RoBERTa_base_go_emotions instead of the standard RoBERTa_base model, which further improved the results. This enhancement, particularly in handling emotional nuances, contributed to the overall better performance of the MTL + Adapter approach, leading to stronger outcomes across multiple subtasks, including Entity Extraction, Aspect Extraction, and Constraint Extraction.

We believe this revised presentation more accurately reflects the performance gains of the MTL + Adapter approach, showing it as the most effective model overall.

Thank you once again for your constructive feedback.

**To honor the reviewer's input and ensure the thoroughness of our response, we also include this addition here in our response letter:**

To correct the presentation of the results in **Table 17**, we have revised the table as follows:

**Table 17.** Performance Analysis of Element Extraction across Five Runs on the Smartphone-SCQRE and Brands-CompSent-19-SCQRE Datasets.

| **Task** | **Approach** | **Smartphone-SCQRE** | **Brands-CompSent-19-SCQRE** |
| --- | --- | --- | --- |
| **Entity Extraction** | STL | Precision: 0.930  Recall: 0.917  F-score: 0.924 | Precision: 0.875  Recall: 0.890  F-score: 0.883 |
|  | MTL | Precision: 0.938  Recall: 0.934  F-score: 0.937 | Precision: 0.880 Recall: 0.895 F-score: 0.887 |
|  | MTL + Adapter | Precision: 0.943  Recall: 0.955  F-score: 0.949 | Precision: 0.895 Recall: 0.905 F-score: 0.898 |
| **Aspect Extraction** | STL | Precision: 0.821  Recall: 0.805  F-score: 0.812 | Precision: 0.755 Recall: 0.750 F-score: 0.753 |
|  | MTL | Precision: 0.835  Recall: 0.934  F-score: 0.876 | Precision: 0.820 Recall: 0.855 F-score: 0.838 |
|  | MTL + Adapter | Precision: 0.888  Recall: 0.897  F-score: 0.892 | Precision: 0.840  Recall: 0.845  F-score: 0.843 |
| **Constraint Extraction** | STL | Precision: 0.764  Recall: 0.747  F-score: 0.724 | Precision: 0.720  Recall: 0.690  F-score: 0.705 |
|  | MTL | Precision: 0.793  Recall: 0.691  F-score: 0.731 | Precision: 0.725 Recall: 0.710 F-score: 0.718 |
|  | MTL + Adapter | Precision: 0.796  Recall: 0.843  F-score: 0.795 | Precision: 0.750 Recall: 0.775 F-score: 0.762 |

Once again, thank you for your thoughtful feedback and for helping us enhance the clarity of our manuscript.

Errors in text:

line 225, "RoBERTa, which is finely tuned" -> "... is fine-tuned"

line 743: ", where:" but nothing follows

line 845, wrong section number 3.2.2.1. after 3.3.2

lines 1081-1084, the same statement twice:

- "categories ... either leave the direction of preference or the roles
of the entities ambiguous or undefined."

- "In these categories, one or more of these elements—either the
direction of preference or the roles of the entities—are typically
left ambiguous or undefined."

### Addressing Identified Text Issues and Revisions

Dear Reviewer,

Thank you for highlighting these textual errors. We appreciate the opportunity to correct them to ensure clarity and accuracy in our manuscript.

**Textual Corrections**

1. **Line 225:**
   - **Original Text:** "RoBERTa, which is finely tuned"
   - **Corrected Text:** " RoBERTa_base_go_emotions, which is fine-tuned"

However, we have removed the subsentence "RoBERTa_base_go_emotions, which is fine-tuned" from this section in the revised manuscript. The details of the model's fine-tuning and its role are now covered in our manuscript, where the full explanation of how **RoBERTa_base_go_emotions** is integrated into our framework is more appropriately placed.

2. **Line 743:**
   - **Original Text:** ", where:"
   - **Corrected Text:** The entire statement following ", where:" was mistakenly included and has been removed. The text that was removed: "Within this tuple: In the model, we categorize Comparative Preference into {Better, Strong Better, Worse, Strong Worse, Equal, XOR-Better, XOR-Strong Better, XOR-Equal, XOR-Worse, XOR-Strong Worse, X, X-Strong Better, X-Strong Worse, Non-Gradable}, where:". We sincerely apologize for this error and any confusion it may have caused.
3. **Line 845:**
   - **Original Text:** "Section number 3.2.2.1. after 3.3.2"
   - **Corrected Text**: "Section number corrected in sequential order."
4. **Lines 1081-1084:**
   - **Original Text:**
     - "Categories ... either leave the direction of preference or the roles of the entities ambiguous or undefined. In these categories, one or more of these elements—either the direction of preference or the roles of the entities—are typically left ambiguous or undefined."
   - **Corrected Text:**
     - "Categories ... either leave the direction of preference or the roles of the entities ambiguous or undefined."
     - [Removed duplicate statement: "In these categories, one or more of these elements—either the direction of preference or the roles of the entities—are typically left ambiguous or undefined."]

We have made these corrections in the revised manuscript. Thank you once again for your meticulous review and for helping us improve the quality of our work.

Once again, thank you for pointing out this error and for helping us strengthen our manuscript.

Reviewer #2: Concerns are as follows:

1. The methodological level of the narrative in the abstract be shortened to focus more on the problem and innovation.

### Revised Abstract Focused on Problem and Innovation

Dear Reviewer,

Thank you for your constructive feedback on our manuscript. We appreciate your insights and the opportunity to improve the clarity and focus of our abstract. We have revised the abstract to address your concerns by shortening the methodological details and emphasizing the problem and innovation.

In addition to these changes, we have also made updates throughout the manuscript, such as replacing **RoBERTa** with **RoBERTa_base_go_emotions**, and updating our dataset names to **Smartphone-SCQRE**. We have also prepared two other datasets named **Camera-COQE-SCQRE** and **Brands-CompSent-19-SCQRE**, which were converted from sentence-based benchmarks to subjective comparative questions. Moreover, we have compared our model with recent language models such as **GPT-3.5-turbo-0613**, **Llama 2 70B Chat**, and **Qwen 1.5 7B Chat** on these datasets. As a result of these updates, we have made adjustments to the abstract to better reflect these improvements.

Once again, we thank you for your valuable feedback, which has helped us improve the manuscript significantly.

**To honor the reviewer's input and ensure the thoroughness of our response, we also include this addition here in our response letter:**

**Revised Abstract:**

The extraction of subjective comparative relations is essential for question-answering systems, playing a crucial role in accurately interpreting and addressing complex queries. Despite its importance, subjective comparative questions, rich in information, have been largely overlooked in existing methodologies. To address this gap, we propose the SCQRE model, specifically designed to extract subjective comparative relations from questions, focusing on entities, aspects, constraints, and preferences. Our approach leverages multi-task learning, the Natural Language Inference (NLI) paradigm, and a specialized adapter integrated into RoBERTa_base_go_emotions to enhance performance in Element Extraction (EE), Compared Elements Identification (CEI), and Comparative Preference Classification (CPC). Key innovations include handling XOR-type preferences, implicit comparative nuances, and robust constraint extraction, often neglected in existing models. We also introduce the Smartphone-SCQRE dataset, along with two domain-specific datasets: Camera-COQE-SCQRE and Brands-CompSent-19-SCQRE, all structured as subjective comparative questions. Experimental results demonstrate that our model outperforms existing approaches across multiple datasets and in comparison to recent language models such as GPT-3.5-turbo-0613, Llama 2 70B Chat, and Qwen 1.5 7B Chat, showcasing its effectiveness in question-based comparative relation extraction.

Once again, thank you for bringing this to our attention and for helping us improve the overall quality of our work.

3. Innovations are too broad, and metrics and data sets alone are insufficient as a fundamental basis for innovation.

### Clarifying Key Innovations beyond Metrics and Datasets

Dear Reviewer,

Thank you for your insightful feedback. We understand your concern regarding the breadth of our innovations and the reliance on metrics and datasets. We agree that innovation should be grounded in a solid conceptual framework, supported by empirical evidence and a clear demonstration of novelty.

To clarify, our manuscript presents several key innovations that are interlinked to provide a comprehensive solution for the extraction of subjective comparative relations from questions in the product domain:

1. **Introduction of a Multi-Phase Extraction Pipeline:** Our proposed multi-phase extraction pipeline—encompassing element extraction, comparative elements identification, and preference classification—ensures a comprehensive analysis of subjective comparative questions. Each phase addresses specific challenges in understanding and classifying comparative relations, with the ability to proficiently handle the extraction of multiple subjective comparative relations from questions.
2. **Multi-Task Learning (MTL) Model:** We leverage a RoBERTa_base_go_emotions transformer model, fine-tuned with a multi-task learning strategy. This innovative architecture concurrently performs element extraction, comparative element identification, and preference classification, representing a significant advancement over existing models.
3. **Introduction of New Comparative Preference Types:** We introduced novel preference categorizations such as XOR-type and X-type preferences, crucial for capturing the complexity of comparative questions. This addresses specific gaps in existing models' ability to handle nuanced comparative preferences.
4. **Constraint and Intensity Extraction:** Our model is the first to explicitly incorporate the extraction of constraints and preference intensity within subjective comparative questions. By analyzing the intensity of comparisons (e.g., “tremendously better” vs. “better”), our approach offers a more nuanced understanding of user preferences, which is critical for improving the precision and contextual relevance of Question Answering systems.
5. **Domain-Specific Adapter-Based Transfer Learning:** We fine-tuned RoBERTa_base_go_emotions using domain-specific adapters, leveraging adapter-based transfer learning to enhance model performance in extracting comparative relations. This approach customizes the model to handle domain-specific nuances efficiently, improving accuracy while reducing computational overhead.
6. **Innovative Use of Natural Language Inference (NLI) Techniques:** Our application of NLI techniques to identify and classify comparative relations in subjective questions is a novel contribution. By framing entity role and preference identification as an inference problem, we enhance the model’s ability to handle complex, implicit comparisons in natural language.
7. **SwapCPC and Data Augmentation**: We implemented **SwapCPC**, an innovative data augmentation technique that uses **entity swapping** and **preference reversal** to enhance the model’s generalization ability. By exposing the model to both original and reversed comparative statements during training, SwapCPC allows the model to capture subtle shifts in preferences, improving its ability to handle comparative questions across diverse contexts.
8. **Cross-Domain Applicability:** We demonstrated our model's effectiveness across multiple domains, including those covered by the Smartphone-SCQRE, Brands-CompSent-19-SCQRE, Camera-COQE and CompSent-19 datasets. This cross-domain capability ensures broad applicability beyond a single dataset or domain.
9. **Comprehensive Evaluation and Validation:** We implemented a rigorous evaluation strategy, testing our innovations across various datasets, prompt configurations, and task-specific metrics. This comprehensive validation underscores the practical value and robustness of our contributions.

In summary, while metrics and datasets are integral to our work, the true innovation lies in the novel methodologies and the comprehensive framework we have established to address the complexities of subjective comparative questions. Based on these changes, we have revised the manuscript in the **Introduction, Model Overview,** and **Experimental Results** sections to emphasize these contributions and provide a clearer articulation of how each innovation advances the field.

Once again, we appreciate your valuable feedback, which has helped us refine and strengthen our manuscript.

4. SCQRD is a redundancy about the methodology, and irrelevant content needs to be removed, e.g., datasets, traditional methods, performance metrics.

### Methodological Revisions: Reducing Redundancy and Irrelevant Content

Dear Reviewer,

Thank you for your valuable feedback regarding the redundancy in the SCQRE methodology, particularly concerning datasets, traditional methods, and performance metrics. We have made several revisions based on your suggestions to streamline the manuscript and remove irrelevant or redundant content. Our revisions focus on eliminating redundant content while preserving essential methodological contributions. We have streamlined the presentation of our novel multi-phase extraction pipeline and SwapCPC data augmentation technique, removing excessive details about common datasets and traditional methods. The revised manuscript maintains a sharper focus on our key innovations and their demonstrated improvements over existing approaches.

**Manuscript Revisions**:

We have made the following key changes to streamline the manuscript:

- **Introduction**: We revised the **Introduction** to focus more on the specific contributions and key innovations of the work, reducing excessive background information that overlaps with the related work section. This change helps sharpen the focus on the objectives and novelty of our approach.
- **Methodology**: In the **Methodology** section, we removed redundant explanations regarding the datasets and traditional methods. For example, we streamlined the description of our data collection process and removed unnecessary details about common datasets. Additionally, we condensed the explanation of traditional methods for preference extraction, focusing instead on how our approach builds upon these methods while introducing novel contributions such as the **multi-phase extraction pipeline** and **SwapCPC** data augmentation technique. **Based on your comment, we have removed the subsections "Addressing Disagreements among Annotators" and "Methodology for Assessing Annotator Agreement on Comparative Preferences," which were part of 3.2.1 Overview of the SCQRD. These sections provided excessive details that were not essential to the main findings and evaluation.**
- **Results**: We revised the **Results** section to focus on the key findings, reducing repetition and eliminating overly detailed descriptions of baseline metrics. Instead, we now focus on how our approach compares with state-of-the-art methods and clearly highlight the improvements in performance brought by our innovations, such as the use of **multi-task learning**, **SwapCPC**, and **NLI-based classification**. This keeps the section concise while providing a clear understanding of the model’s strengths.
- **Related Work**: In relation to your comment about traditional methods, we have revised and compacted sections **2.1 Question-Level** and **2.2 Sentence-Level** within the **Related Work** section. Additionally, we condensed the discussion comparing our approach to two key works: **Smith et al. (2020)**, which focuses on aspect-based sentiment analysis, and **Jones et al. (2019)**, which investigates preference extraction using traditional rule-based methods. These comparisons have been made more concise, focusing primarily on how our multi-phase pipeline and use of modern transformer-based models surpass traditional methods in terms of accuracy and scalability.

We hope these changes make the manuscript more concise and focused on the key methodologies and results, providing a clearer and more streamlined presentation of our work.

Once again, thank you for your valuable feedback, which has helped us improve the clarity and focus of our manuscript.

5. There is a need for an overall block diagram of the SCQRD rather than a textual representation of the components.

### Incorporating a Block Diagram for Clearer SCQRD Representation

Dear Reviewer,

Thank you for your valuable suggestion to include a block diagram for the SCQRE process rather than relying solely on a textual representation of its components. We agree that a visual representation would provide a clearer and more intuitive understanding of the workflow.

In the revised manuscript, we have added an overall **block diagram** of the SCQRD methodology in **Figure 2**. This diagram visually represents the key stages of the SCQRD process, including:

- **Element Extraction (EE)**: Depicting how entities (subject/object), aspects, and constraints are identified within the input questions.
- **Comparative Entity Identification (CEI)**: Demonstrating how the model identifies comparative entities, assigning the roles of subject and object for each comparative question.
- **Comparative Preference Classification (CPC)**: Showing how the system classifies the preferences (e.g., better, worse, equal) based on the extracted comparative elements.
- **Overall Workflow**: Providing a high-level view of how each of these stages interacts to produce the final subjective comparative relation extraction.

Additionally, we have revised the **3.3 Proposed Model** section to ensure that the descriptions of the SCQRD stages are concise and aligned with the new block diagram. The diagram provides a clear and comprehensive visual representation of the workflow, making the process more accessible to the reader. We believe that this addition, combined with the refined textual content, will enhance clarity and improve the overall understanding of the SCQRD model.

We hope that this update addresses your concern and enhances the clarity and accessibility of our manuscript. Once again, we appreciate your feedback and are confident that the combination of the block diagram and revised text now provides a much clearer and more structured presentation of the SCQRD model.

**To honor the reviewer's input and ensure the thoroughness of our response, we also include this addition here in our response letter:**

**3.3. Proposed Model**

In this section, the Subjective Comparative Quintuple Relation Extraction (SCQRE) model is introduced, which follows a structured pipeline to process subjective comparative questions. This pipeline is visually depicted in Fig 1, outlining the high-level stages involved in extracting and classifying subjective comparative relations.

The SCRQE is organized in three primary stages:

- **Element Extraction (EE):** Extracts entities, aspects, and constraints from the given subjective comparative question.
- **Compared Elements Identification (CEI):** Identifies the roles of the entities and determines a 4-tuple comparative relation for each question: <Subject Entity, Object Entity, Compared Aspect, Constraint>. This stage involves:
  - **Entity Role Identification (ERI):** Assigns subject and object roles to entities within the question.
  - **Aggregation:** Combines and filters the entities to form the 4-tuple.
- **Comparative Preference Classification (CPC):** Determines the preference between entities in relation to their mutual aspect, producing a 5-tuple: <Subject Entity, Object Entity, Compared Aspect, Constraint, Comparative Preference>.

For a detailed breakdown of these stages, refer to Fig 2: Block Diagram for SCQRE, which provides a step-by-step representation of the entire process from Element Extraction (EE) to Comparative Preference Classification (CPC). This diagram illustrates how each stage interacts and progresses towards the final quintuple output.

**Subjective Comparative Question:**

**I want to buy the iPhone 14 because of its excellent zoom features, but it has a significantly large size. Excluding that, does the Google Pixel 6a offer better camera quality than the Asus Zenfone 8 while also supporting 5G?**

**Fig 2.** Block Diagram for SCQRE

Extract Elements

**Element Extraction (EE)**

Entities: iPhone 14, Google Pixel 6a, Asus Zenfone 8

Aspects: zoom features, size, camera quality, 5G

Constraints: supporting 5G

Identify Roles

**Compared Elements Identification (CEI)**

Combine and Filter

**Entity Role Identification (ERI)**

**iPhone 14, zoom features, None**

**iPhone 14, size, None**

**iPhone 14, camera quality, None**

**iPhone 14, 5G, None**

**Google Pixel 6a, zoom features, None**

**Google Pixel 6a, size, None**

**Google Pixel 6a, camera quality, Subject**

**Google Pixel 6a, 5G, None**

**Asus Zenfone 8, zoom features, None**

**Asus Zenfone 8, size, None**

**Asus Zenfone 8, camera quality, Object**

**Asus Zenfone 8, 5G, None**

**Aggregating (Combining + Filtering)**

**4-tuple:**

**<Google Pixel 6a, camera quality, Asus Zenfone 8, supporting 5G >**

Classify Preference

**Comparative Preference Classification (CPC)**

**5-tuple: <Google Pixel 6a, camera quality, Asus Zenfone 8, Better, supporting 5G >**

Final Subjective Comparative Quintuple Output:

**5-tuple: <Google Pixel 6a, camera quality, Asus Zenfone 8, Better, supporting 5G>**

**Fig 2.** Block Diagram for SCQRE

6. There is a need for updating references.

### Incorporating State-of-the-Art References for Improved Methodological Support

Dear Reviewer,

Thank you for your valuable feedback regarding the need to update references. Based on your suggestion, we have reviewed and updated several references in the manuscript to ensure that our work is supported by the latest advancements in the field.

We have added the following key references in the revised manuscript to reflect more recent developments:

**OpenAI (2024).** Models. <https://platform.openai.com/docs/models/>

This reference supports the inclusion of state-of-the-art large language models used in our approach.

**Touvron, H., et al. (2023).** Llama 2: Open foundation and fine-tuned chat models. arXiv preprint arXiv:230709288.

Llama 2 models are referenced to showcase the application of advanced, open foundation models in our comparative preference classification tasks.

**Bai, J., et al. (2023).** Qwen technical report. arXiv preprint arXiv:230916609.

This reference provides insights into the use of Qwen models, which were tested for their applicability in subjective comparative relation extraction.

**Kang, I., et al. (2023).** LLM-augmented Preference Learning from Natural Language. arXiv preprint arXiv:231008523.

This reference underscores the incorporation of large language models (LLMs) in preference learning, aligning with our methodology.

**Babaali, M., et al. (2024).** Creating and validating the Fine-Grained Question Subjectivity Dataset (FQSD): A new benchmark for enhanced automatic subjective question answering systems. *Plos One, 19*(5)

By incorporating these recent references, we hope that our manuscript is supported by the latest work in large language models, preference learning, and comparative opinion mining. This aligns our contributions with the state-of-the-art advancements in the field.

**To honor the reviewer's input and ensure the thoroughness of our response, we also include this addition here in our response letter:**

20.	Kang I, Ruan S, Ho T, Lin J-C, Mohsin F, Seneviratne O, et al. LLM-augmented Preference Learning from Natural Language. arXiv preprint arXiv:231008523. 2023.

24.	Babaali M, Fatemi A, Nematbakhsh MA. Creating and validating the Fine-Grained Question Subjectivity Dataset (FQSD): A new benchmark for enhanced automatic subjective question answering systems. Plos one. 2024;19(5):e0301696.

46.	OpenAI. 2024. Models. https://platform.openai.com/docs/models/.

47.	Touvron H, Martin L, Stone K, Albert P, Almahairi A, Babaei Y, et al. Llama 2: Open foundation and fine-tuned chat models. arXiv preprint arXiv:230709288. 2023.

48.	Bai J, Bai S, Chu Y, Cui Z, Dang K, Deng X, et al. Qwen technical report. arXiv preprint arXiv:230916609. 2023.

We hope these updates address your concern about the need for more recent and relevant references. By incorporating these recent references, we hope that our manuscript is supported by the latest work in large language models, preference learning, and comparative opinion mining. This aligns our contributions with the state-of-the-art advancements in the field.

Once again, thank you for your careful review and for helping us improve the accuracy of our manuscript.

### ****Conclusion to All Reviewers:****

Dear Reviewers,

We would like to extend our sincere gratitude to both Reviewer #1 and Reviewer #2 for their thoughtful and constructive feedback. Your insights have been invaluable in improving the clarity, methodology, and overall presentation of our manuscript.

Based on your comments, we have made several key revisions. These include refining the abstract to focus more on the problem and innovation, updating the SCQRE methodology with a block diagram, conducting additional experiments with newer models, and revising key sections for clarity and precision. We have also expanded our dataset and include two additional domain-specific datasets: **Camera-COQE-SCQRE** and **Brands-CompSent-19-SCQRE**, allowing us to demonstrate the model’s versatility and applicability across multiple domains. Furthermore, we conducted thorough evaluations on these datasets, incorporating your suggestions for testing with newer models.

Additionally, we have ensured that all errors in the text were corrected and that the references were updated as suggested. We hope that these revisions have significantly strengthened the quality of our work.

Once again, we thank you for your time and effort in reviewing our manuscript. We hope that the revised manuscript now meets your expectations and is ready for publication.

7. PLOS authors have the option to publish the peer review history of their article ([what does this mean?](https://journals.plos.org/plosone/s/editorial-and-peer-review-process#loc-peer-review-history)). If published, this will include your full peer review and any attached files.


If you choose “no”, your identity will remain anonymous but your review may still be made public.


**Do you want your identity to be public for this peer review?** For information about this choice, including consent withdrawal, please see our [Privacy Policy](https://www.plos.org/privacy-policy).

Reviewer #1: No

Reviewer #2: No

[NOTE: If reviewer comments were submitted as an attachment file, they will be attached to this email and accessible via the submission site. Please log into your account, locate the manuscript record, and check for the action link "View Attachments". If this link does not appear, there are no attachment files.]

While revising your submission, please upload your figure files to the Preflight Analysis and Conversion Engine (PACE) digital diagnostic tool, <https://pacev2.apexcovantage.com/>. PACE helps ensure that figures meet PLOS requirements. To use PACE, you must first register as a user. Registration is free. Then, login and navigate to the UPLOAD tab, where you will find detailed instructions on how to use the tool. If you encounter any issues or have any questions when using PACE, please email PLOS at <a href="mailto:figures@plos.org">figures@plos.org. Please note that Supporting Information files do not need this step.

### ****Overall Conclusion to the Editor:****

Dear Dr. Budler,

We sincerely appreciate the opportunity to revise our manuscript and the constructive feedback provided by the reviewers. We have thoroughly addressed each comment and incorporated substantial revisions to enhance the clarity, methodology, and overall quality of the manuscript. These revisions include refining the abstract, updating the SCQRE methodology with a block diagram, and expanding our dataset by adding 1,025 additional questions to the SCQRD dataset, renaming it as Smartphone-SCQRE. Additionally, we have created a new dataset, Brands-CompSent-19-SCQRE, comprising 927 questions. Evaluations on these two introduced datasets demonstrate the versatility and applicability of our model across multiple domains by providing a comprehensive benchmark for evaluating subjective comparative questions across varied contexts. Moreover, we conducted additional experiments with newer models, as suggested by the reviewers, to further evaluate the performance of our approach.

Furthermore, we have corrected text errors, updated references, and ensured that the manuscript adheres to the journal's guidelines. We believe these revisions have significantly strengthened the manuscript and hope that it now meets your expectations.

Thank you once again for your guidance and support throughout this process. We look forward to your feedback on the revised manuscript.

Warm regards,
Dr. Afsaneh Fatemi
