# Supplementary material for: SCRQE: Subjective comparative relation quintuple extraction from questions in product domain
Source: PLoS One. 2025 May 27;20(5):e0319824. doi: 10.1371/journal.pone.0319824 (PMC12112347; doi:10.1371/journal.pone.0319824)
Supplement: Attachment — Submitted filename: Detailed_Response_to_Reviewers_auresp_3.docx [file pone.0319824.s006.docx]

### View Letter

| **Date:** | Jan 10 2025 06:13AM |
| --- | --- |
| **To:** | "Afsaneh Fatemi" a_fatemi@eng.ui.ac.ir |
| **From:** | "PLOS ONE" plosone@plos.org |
| **Subject:** | PLOS ONE Decision: Revision required [PONE-D-23-39359R2] |

PONE-D-23-39359R2

SCRQE: Subjective Comparative Relation Quintuple Extraction from Questions in Product Domain

PLOS ONE

Dear Dr. Fatemi,

Thank you for submitting your manuscript to PLOS ONE. After careful consideration, we feel that it has merit but does not fully meet PLOS ONE’s publication criteria as it currently stands. Therefore, we invite you to submit a revised version of the manuscript that addresses the points raised during the review process.

Please submit your revised manuscript by Feb 24 2025 11:59PM. If you will need more time than this to complete your revisions, please reply to this message or contact the journal office at [plosone@plos.org](mailto:plosone@plos.org). When you're ready to submit your revision, log on to https://www.editorialmanager.com/pone/ and select the 'Submissions Needing Revision' folder to locate your manuscript file.

Please include the following items when submitting your revised manuscript:

- A rebuttal letter that responds to each point raised by the academic editor and reviewer(s). You should upload this letter as a separate file labeled 'Response to Reviewers'.
- A marked-up copy of your manuscript that highlights changes made to the original version. You should upload this as a separate file labeled 'Revised Manuscript with Track Changes'.
- An unmarked version of your revised paper without tracked changes. You should upload this as a separate file labeled 'Manuscript'.

If you would like to make changes to your financial disclosure, please include your updated statement in your cover letter. Guidelines for resubmitting your figure files are available below the reviewer comments at the end of this letter.

If applicable, we recommend that you deposit your laboratory protocols in protocols.io to enhance the reproducibility of your results. Protocols.io assigns your protocol its own identifier (DOI) so that it can be cited independently in the future. For instructions see: <https://journals.plos.org/plosone/s/submission-guidelines#loc-laboratory-protocols>. Additionally, PLOS ONE offers an option for publishing peer-reviewed Lab Protocol articles, which describe protocols hosted on protocols.io. Read more information on sharing protocols at <https://plos.org/protocols?utm_medium=editorial-email&utm_source=authorletters&utm_campaign=protocols>.

We look forward to receiving your revised manuscript.

Kind regards,

Leona Cilar Budler
Academic Editor
PLOS ONE

### Response to Revision Request for Manuscript ID [PONE-D-23-39359R2]

Dear Dr. Leona Cilar Budler,

Thank you for your detailed feedback and the opportunity to revise our manuscript titled "SCRQE: Subjective Comparative Relation Quintuple Extraction from Questions in Product Domain," with ID [PONE-D-23-39359R2]. We greatly appreciate the constructive comments from the reviewers and recognize the significant value they add to our study.

We are diligently addressing the points raised during the review process and are confident that our revisions will meet PLOS ONE’s publication criteria. We aim to submit the revised manuscript by the set deadline of February 24, 2025. Should we require additional time to complete our revisions, we will notify you promptly.

As requested, we will include the following items in our submission:

1. **Rebuttal Letter:** A document labeled 'Response to Reviewers', addressing each point raised by the academic editor and reviewers.
2. **Marked-Up Copy of the Manuscript:** This document will highlight changes made to the original version and will be labeled 'Revised Manuscript with Track Changes'.
3. **Unmarked Version of the Revised Paper:** This document will be uploaded as 'Manuscript' and will not contain tracked changes.

Additionally, we have included a comprehensive title page within the main manuscript document, listing all authors, their affiliations, and clearly indicating the corresponding author, as per PLOS ONE’s author instructions. All references have been meticulously converted to **Vancouver style** and checked for accuracy, resulting in the addition of References [4] and [6], the replacement of the former Reference [22] with Reference [25], and the renumbering of existing references to maintain consistency and adherence to the journal’s formatting guidelines. In response to Reviewer #1, we have introduced the **RoBERTa_base_go_emotions** model earlier in the manuscript, corrected its citation to include the fine-tuned **GoEmotions** resource, and clarified our focus on prompt-based evaluations over fine-tuning generative models. Specifically, while these generative models can indeed be fine-tuned with dedicated classification layers, we chose to focus on prompt-based evaluations to provide a straightforward zero-/few-shot comparison across different model families, with fine-tuning being a promising direction for future work. These changes enhance the clarity and quality of our manuscript, aligning it with the journal’s requirements.

We will also review our financial disclosure statement and update it if necessary. Although our study does not involve laboratory protocols, we have ensured that our data preparation, model implementation, and experimental setups are described in sufficient detail to ensure reproducibility. Additionally, all datasets and relevant code have been made publicly available as stated in our Data Availability section.

Thank you once again for considering our work. We look forward to the opportunity to enhance our manuscript and contribute to PLOS ONE.

**Sincerely,**

Afsaneh Fatemi
Corresponding Author on behalf of all co-authors

**Journal Requirements:**

Please review your reference list to ensure that it is complete and correct. If you have cited papers that have been retracted, please include the rationale for doing so in the manuscript text, or remove these references and replace them with relevant current references. Any changes to the reference list should be mentioned in the rebuttal letter that accompanies your revised manuscript. If you need to cite a retracted article, indicate the article’s retracted status in the References list and also include a citation and full reference for the retraction notice.

### Response to the Editor

**Dear Editor,**

We appreciate your careful review of our manuscript and the opportunity to address the journal’s requirements. Below, please find our point-by-point response regarding our updated reference list:

1. **Review and Update of Reference List**
   - We have thoroughly examined our entire reference list to ensure it is complete, accurate, and current.
   - **No Retracted References:** We confirm that none of the sources cited in our revised manuscript are retracted. However, we replaced the former Reference 22 with a new Reference 25 to include a more current and relevant source.
   - **Conversion to Vancouver Style:** All references have been converted to Vancouver style and have been checked for accuracy to ensure they adhere to the journal’s formatting requirements.
   - **Newly Added References:** We have introduced three new references in this revision:
     1. **References 4 and 6:** These pertain to the **roberta-base_go_emotions** model and the **GoEmotions** dataset, respectively. Initially, we only included a Hugging Face footnote for *SamLowe/roberta-base-go_emotions*, but we now explicitly cite both the specialized model and **GoEmotions** to clarify the lineage from **RoBERTa** to the fine-tuned variant.
     2. **Reference 25:** This replaces the previous Reference 22 in the earlier version of our manuscript. We opted for a more recent source that provides updated findings relevant to our research topic.
   - As a result of adding these references, the numbering of subsequent items in the list has shifted accordingly. Additionally, **Reference 30** in the previous manuscript has been moved forward to **Reference 5** as it is now mentioned earlier in the revised manuscript.
2. **Rationale for Retractions or Replacements**
   - As noted above, no citations in our manuscript are retracted; thus, we did not need to provide any retraction notices.
   - The only replacement involves the introduction of **Reference 25** in place of the former **Reference 22**, due to its more comprehensive and up-to-date information.
3. **Mention of Reference List Changes in Rebuttal Letter**
   - In accordance with the journal’s guidelines, we confirm here that our **only changes** involve adding these three new references (4, 6, and 25), thereby causing a renumbering of some existing entries.
   - **References 4 & 6:** In Section 3.3.1, we clarify the fine-tuning lineage by citing the original RoBERTa paper [Liu et al., 2019], the *SamLowe/roberta-base-go_emotions* model (including its Hugging Face link), and the **GoEmotions** dataset [Demszky et al., 2020]. We specifically highlight how the **roberta-base_go_emotions** variant differs from base RoBERTa in its multi-label emotion classification capabilities.
   - **Reference 25:** This reference replaces the old 22 in our earlier manuscript and reflects a more updated source with findings that are directly applicable to our study.
   - **Additional Renumbering:** Due to the earlier mention in the revised text, the **previous Reference 30** now appears **as Reference 5** in the new manuscript.
   - **Conversion to Vancouver Style:** We have converted all references to Vancouver style, ensuring consistency and adherence to the journal’s formatting guidelines. Each reference has been thoroughly checked for correctness.

**Additional Notes for Clarity**

- **Reference Additions and Replacements:**
  - **References 4 and 6** were added to provide direct citations for the **roberta-base_go_emotions** model and the **GoEmotions** dataset, respectively. These additions clarify the model’s lineage and its relevance to our study.
  - **Reference 25** was introduced to replace **Reference 22** with a more recent and relevant source that enhances the credibility and up-to-dateness of our research.
- **Conversion to Vancouver Style:**
  - All references have been meticulously converted to Vancouver style, adhering to the journal’s specific formatting guidelines. This includes proper author formatting, title capitalization, journal abbreviations, and accurate page numbering.
- **Renumbering Impact:**
  - The addition of new references 4 and 6, along with the replacement of 22 with 25, has resulted in a systematic renumbering of subsequent references. For example, the previous Reference 30 is now listed as Reference 5 due to its earlier mention in the revised manuscript.

We have ensured that these changes enhance the clarity and accuracy of our manuscript, aligning it with the journal’s standards and requirements. We trust that these revisions fulfill the journal’s requirements, and we remain available to provide any additional clarifications or materials as necessary. Once again, we appreciate your guidance and consideration.

**To honor the reviewer's input and ensure the thoroughness of our response, we have included references to both the previous version of the manuscript and the current revision in our response letter:**

**Previous version of the manuscript:**

**6. References**

1.	Moghaddam, S. and M. Ester. *AQA: aspect-based opinion question answering*. in *2011 IEEE 11th International Conference on Data Mining Workshops*. 2011. IEEE.

2.	Yu, J., Z.-J. Zha, and T.-S. Chua. *Answering opinion questions on products by exploiting hierarchical organization of consumer reviews*. in *Proceedings of the 2012 joint conference on empirical methods in natural language processing and computational natural language learning*. 2012.

3.	Bayoudhi, A., H. Ghorbel, and L. Hadrich Belguith. *Question answering system for dialogues: A new taxonomy of opinion questions*. in *Flexible Query Answering Systems: 10th International Conference, FQAS 2013, Granada, Spain, September 18-20, 2013. Proceedings 10*. 2013. Springer.

4.	Pavlopoulos, I., *Aspect based sentiment analysis.* Athens University of Economics and Business, 2014.

5.	Liu, Z., R. Xia, and J. Yu. *Comparative opinion quintuple extraction from product reviews*. in *Proceedings of the 2021 conference on empirical methods in natural language processing*. 2021.

6.	Panchenko, A., et al., *Categorizing comparative sentences.* arXiv preprint arXiv:1809.06152, 2018.

7.	Li, S., et al., *Comparable entity mining from comparative questions.* IEEE transactions on knowledge and data engineering, 2011. **25**(7): p. 1498-1509.

8.	REDDY, V. and B. KMahesh, *A Method for Comparable Mining from Comparative Questions.* 2014.

9.	Saelan, A., A. Purwarianti, and D. Widyantoro. *Question analysis for Indonesian comparative question*. in *Journal of Physics: Conference Series*. 2017. IOP Publishing.

10.	Saelan, A., A. Purwarianti, and D.H. Widyantoro, *Answering Comparison in Indonesian Question Answering System with Database.* International Journal on Electrical Engineering & Informatics, 2018. **10**(4).

11.	Liu, Z., C.-X. Qin, and Y.-J. Zhang, *Mining product competitiveness by fusing multisource online information.* Decision Support Systems, 2021. **143**: p. 113477.

12.	Jindal, N. and B. Liu. *Mining comparative sentences and relations*. in *Aaai*. 2006.

13.	Xu, K., et al., *Mining comparative opinions from customer reviews for competitive intelligence.* Decision support systems, 2011. **50**(4): p. 743-754.

14.	Arora, J., et al. *Extracting entities of interest from comparative product reviews*. in *Proceedings of the 2017 ACM on Conference on Information and Knowledge Management*. 2017.

15.	Yang Z, Xu F, Yu J, Xia R, editors. UniCOQE: Unified comparative opinion quintuple extraction as a set. Findings of the Association for Computational Linguistics: ACL 2023; 2023.

16.	Gao, F., et al., *End-to-end comparative opinion quintuple extraction as bipartite set prediction with dynamic structure pruning.* Expert Systems with Applications, 2024. **245**: p. 123058.

17.	Wang, H., et al., *Competitiveness analysis through comparative relation mining: evidence from restaurants’ online reviews.* Industrial Management & Data Systems, 2017. **117**(4): p. 672-687.

18.	Liu, D., L. Wang, and Y. Shao. *Multi-task learning neural networks for comparative elements extraction*. in *Chinese Lexical Semantics: 21st Workshop, CLSW 2020, Hong Kong, China, May 28–30, 2020, Revised Selected Papers 21*. 2021. Springer.

19.	Younis, U., et al., *Applying machine learning techniques for performing comparative opinion mining.* Open Computer Science, 2020. **10**(1): p. 461-477.

20.	Kang I, Ruan S, Ho T, Lin J-C, Mohsin F, Seneviratne O, et al. LLM-augmented Preference Learning from Natural Language. arXiv preprint arXiv:231008523. 2023.

21.	Ma, N., et al. *Entity-aware dependency-based deep graph attention network for comparative preference classification*. in *Proceedings of Annual Meeting of the Association for Computational Linguistics (ACL-2020)*. 2020.

22.	Kang, Y., *A Hierarchical Framework for Online Product Review Helpfulness Assessment.* 2017.

23.	Cruz, I., A.F. Gelbukh, and G. Sidorov, *Implicit Aspect Indicator Extraction for Aspect based Opinion Mining.* Int. J. Comput. Linguistics Appl., 2014. **5**(2): p. 135-152.

24.	Babaali M, Fatemi A, Nematbakhsh MA. Creating and validating the Fine-Grained Question Subjectivity Dataset (FQSD): A new benchmark for enhanced automatic subjective question answering systems. Plos one. 2024;19(5):e0301696.

25.	Augustyniak, Ł., T. Kajdanowicz, and P. Kazienko. *Aspect detection using word and char embeddings with (Bi) LSTM and CRF*. in *2019 IEEE second international conference on artificial intelligence and knowledge engineering (AIKE)*. 2019. IEEE.

26.	Fleiss, J.L., *Measuring nominal scale agreement among many raters.* Psychological bulletin, 1971. **76**(5): p. 378.

27.	Cohen, J., *Weighted kappa: Nominal scale agreement provision for scaled disagreement or partial credit.* Psychological bulletin, 1968. **70**(4): p. 213.

28.	Krippendorff, K., *Reliability in content analysis: Some common misconceptions and recommendations.* Human communication research, 2004. **30**(3): p. 411-433.

29.	Krippendorff, K., *Validity in content analysis*, in *Computerstrategien für die Kommunikationsanalyse*. 1980.

30.	Liu, Y., et al., *Roberta: A robustly optimized bert pretraining approach.* arXiv preprint arXiv:1907.11692, 2019.

31.	Worsham, J. and J. Kalita, *Multi-task learning for natural language processing in the 2020s: Where are we going?* Pattern Recognition Letters, 2020. **136**: p. 120-126.

32.	Sun, C., L. Huang, and X. Qiu, *Utilizing BERT for aspect-based sentiment analysis via constructing auxiliary sentence.* arXiv preprint arXiv:1903.09588, 2019.

33.	Tran, T.U., H.T.T. Hoang, and H.X. Huynh. *Aspect extraction with bidirectional GRU and CRF*. in *2019 IEEE-RIVF International Conference on Computing and Communication Technologies (RIVF)*. 2019. IEEE.

34.	Da’u, A. and N. Salim, *Aspect extraction on user textual reviews using multi-channel convolutional neural network.* PeerJ Computer Science, 2019. **5**: p. e191.

35.	Feng, C., et al., *Pre-trained language embedding-based contextual summary and multi-scale transmission network for aspect extraction.* Procedia Computer Science, 2020. **174**: p. 40-49.

36.	Wu, M., W. Wang, and S.J. Pan. *Deep weighted maxsat for aspect-based opinion extraction*. in *Proceedings of the 2020 conference on empirical methods in natural language processing (EMNLP)*. 2020.

37.	Zschornack Rodrigues Saraiva, F., T. Linhares Coelho da Silva, and J.A. Fernandes de Macêdo. *Aspect term extraction using deep learning model with minimal feature engineering*. in *Advanced Information Systems Engineering: 32nd International Conference, CAiSE 2020, Grenoble, France, June 8–12, 2020, Proceedings 32*. 2020. Springer.

38.	Chen, Z. and T. Qian. *Enhancing aspect term extraction with soft prototypes*. in *Proceedings of the 2020 Conference on Empirical Methods in Natural Language Processing (EMNLP)*. 2020.

39.	Wei, Z., et al. *Don’t eclipse your arts due to small discrepancies: Boundary repositioning with a pointer network for aspect extraction*. in *Proceedings of the 58th annual meeting of the association for computational linguistics*. 2020.

40.	Shi, J., et al., *BeeAE: effective aspect term extraction with artificial bee colony.* The Journal of Supercomputing, 2022. **78**(16): p. 17969-17991.

41.	Pour, A.A.M. and S. Jalili. *Aspects extraction for aspect level opinion analysis based on deep CNN*. in *2021 26th International Computer Conference, Computer Society of Iran (CSICC)*. 2021. IEEE.

42.	Wang, R., et al., *Post-processing method with aspect term error correction for enhancing aspect term extraction.* Applied Intelligence, 2022. **52**(14): p. 15751-15763.

43.	Ganapathibhotla, M. and B. Liu. *Mining opinions in comparative sentences*. in *Proceedings of the 22nd international conference on computational linguistics (Coling 2008)*. 2008.

44.	Li, Z., et al., *Powering comparative classification with sentiment analysis via domain adaptive knowledge transfer.* arXiv preprint arXiv:2109.03819, 2021.

45.	Xu Q, Hong Y, Zhao F, Song K, Chen J, Kang Y, et al., editors. GCN-based End-to-End Model for Comparative Opinion Quintuple Extraction. 2023 International Joint Conference on Neural Networks (IJCNN); 2023: IEEE.

46.	OpenAI. 2024. Models. https://platform.openai.com/docs/models/.

47.	Touvron H, Martin L, Stone K, Albert P, Almahairi A, Babaei Y, et al. Llama 2: Open foundation and fine-tuned chat models. arXiv preprint arXiv:230709288. 2023.

48.	Bai J, Bai S, Chu Y, Cui Z, Dang K, Deng X, et al. Qwen technical report. arXiv preprint arXiv:230916609. 2023.

**Current revision of the manuscript:**

**6. References**

1.	Moghaddam S, Ester M, editors. AQA: aspect-based opinion question answering. 2011 IEEE 11th International Conference on Data Mining Workshops; 2011: IEEE.

2.	Yu J, Zha Z-J, Chua T-S, editors. Answering opinion questions on products by exploiting hierarchical organization of consumer reviews. Proceedings of the 2012 joint conference on empirical methods in natural language processing and computational natural language learning; 2012.

3.	Bayoudhi A, Ghorbel H, Hadrich Belguith L, editors. Question answering system for dialogues: A new taxonomy of opinion questions. Flexible Query Answering Systems: 10th International Conference, FQAS 2013, Granada, Spain, September 18-20, 2013 Proceedings 10; 2013: Springer.

4.	Lowe S. roberta-base-go_emotions [Internet]. HuggingFace. 2021 [cited 2024 Sep 23]. Available from: <https://huggingface.co/SamLowe/roberta-base-go_emotions>

5.	Liu Y. Roberta: A robustly optimized bert pretraining approach. arXiv preprint arXiv:190711692. 2019;364.

6.	Demszky D, Movshovitz-Attias D, Ko J, Cowen A, Nemade G, Ravi S. GoEmotions: A dataset of fine-grained emotions. arXiv preprint arXiv:200500547. 2020.

7.	Pavlopoulos I. Aspect based sentiment analysis [Doctoral dissertation]. Athens (Greece): Athens University of Economics and Business; 2014.

8.	Liu Z, Xia R, Yu J, editors. Comparative opinion quintuple extraction from product reviews. Proceedings of the 2021 conference on empirical methods in natural language processing; 2021.

9.	Panchenko A, Bondarenko A, Franzek M, Hagen M, Biemann C. Categorizing comparative sentences. arXiv preprint arXiv:180906152. 2018.

10.	Li S, Lin C-Y, Song Y-I, Li Z. Comparable entity mining from comparative questions. IEEE transactions on knowledge and data engineering. 2011;25(7):1498-509.

11.	Reddy V, Babu KM. A method for comparable mining from comparative questions. Int J Sci Eng Technol Res. 2014 Oct;3(30):5942–5.

12.	Saelan A, Purwarianti A, Widyantoro D, editors. Question analysis for Indonesian comparative question. Journal of Physics: Conference Series; 2017: IOP Publishing.

13.	Saelan A, Purwarianti A, Widyantoro DH. Answering Comparison in Indonesian Question Answering System with Database. International Journal on Electrical Engineering & Informatics. 2018;10(4).

14.	Liu Z, Qin C-X, Zhang Y-J. Mining product competitiveness by fusing multisource online information. Decision Support Systems. 2021;143:113477.

15.	Jindal N, Liu B. Mining comparative sentences and relations. In: Proceedings of the AAAI Conference on Artificial Intelligence; 2006 Jul; p.9.

16.	Xu K, Liao SS, Li J, Song Y. Mining comparative opinions from customer reviews for competitive intelligence. Decision support systems. 2011;50(4):743-54.

17.	Arora J, Agrawal S, Goyal P, Pathak S, editors. Extracting entities of interest from comparative product reviews. Proceedings of the 2017 ACM on Conference on Information and Knowledge Management; 2017.

18.	Yang Z, Xu F, Yu J, Xia R, editors. UniCOQE: Unified comparative opinion quintuple extraction as a set. Findings of the Association for Computational Linguistics: ACL 2023; 2023.

19.	Gao F, Liu Y, Fu W, Zhang M, Ballard A, Zhao L. End-to-end comparative opinion quintuple extraction as bipartite set prediction with dynamic structure pruning. Expert Systems with Applications. 2024;245:123058.

20.	Wang H, Gao S, Yin P, Liu JN-K. Competitiveness analysis through comparative relation mining: evidence from restaurants’ online reviews. Industrial Management & Data Systems. 2017;117(4):672-87.

21.	Liu D, Wang L, Shao Y, editors. Multi-task learning neural networks for comparative elements extraction. Chinese Lexical Semantics: 21st Workshop, CLSW 2020, Hong Kong, China, May 28–30, 2020, Revised Selected Papers 21; 2021: Springer.

22.	Younis U, Asghar MZ, Khan A, Khan A, Iqbal J, Jillani N. Applying machine learning techniques for performing comparative opinion mining. Open Computer Science. 2020;10(1):461-77.

23.	Kang I, Ruan S, Ho T, Lin J-C, Mohsin F, Seneviratne O, et al. LLM-augmented Preference Learning from Natural Language. arXiv preprint arXiv:231008523. 2023.

24.	Ma N, Mazumder S, Wang H, Liu B, editors. Entity-aware dependency-based deep graph attention network for comparative preference classification. Proceedings of the 58th annual meeting of the association for computational linguistics; 2020.

25.	Kathuria A, Gupta A, Singla R. AOH-Senti: aspect-oriented hybrid approach to sentiment analysis of students’ feedback. SN Computer Science. 2023;4(2):152.

26.	Cruz I, Gelbukh AF, Sidorov G. Implicit Aspect Indicator Extraction for Aspect based Opinion Mining. Int J Comput Linguistics Appl. 2014;5(2):135-52.

27.	Babaali M, Fatemi A, Nematbakhsh MA. Creating and validating the Fine-Grained Question Subjectivity Dataset (FQSD): A new benchmark for enhanced automatic subjective question answering systems. Plos one. 2024;19(5):e0301696.

28.	Augustyniak Ł, Kajdanowicz T, Kazienko P, editors. Aspect detection using word and char embeddings with (Bi) LSTM and CRF. 2019 IEEE second international conference on artificial intelligence and knowledge engineering (AIKE); 2019: IEEE.

29.	Fleiss JL. Measuring nominal scale agreement among many raters. Psychological bulletin. 1971;76(5):378.

30.	Cohen J. Weighted kappa: Nominal scale agreement provision for scaled disagreement or partial credit. Psychological bulletin. 1968;70(4):213.

31.	Krippendorff K. Reliability in content analysis: Some common misconceptions and recommendations. Human communication research. 2004;30(3):411-33.

32.	Krippendorff K. Validity in content analysis. Computerstrategien für die Kommunikationsanalyse. 1980;69:45p.

33.	Worsham J, Kalita J. Multi-task learning for natural language processing in the 2020s: Where are we going? Pattern Recognition Letters. 2020;136:120-6.

34.	Sun C, Huang L, Qiu X. Utilizing BERT for aspect-based sentiment analysis via constructing auxiliary sentence. arXiv preprint arXiv:190309588. 2019.

35.	Tran TU, Hoang HTT, Huynh HX, editors. Aspect extraction with bidirectional GRU and CRF. 2019 IEEE-RIVF International Conference on Computing and Communication Technologies (RIVF); 2019: IEEE.

36.	Da’u A, Salim N. Aspect extraction on user textual reviews using multi-channel convolutional neural network. PeerJ Computer Science. 2019;5:e191.

37.	Feng C, Rao Y, Nazir A, Wu L, He L. Pre-trained language embedding-based contextual summary and multi-scale transmission network for aspect extraction. Procedia Computer Science. 2020;174:40-9.

38.	Wu M, Wang W, Pan SJ, editors. Deep weighted maxsat for aspect-based opinion extraction. Proceedings of the 2020 conference on empirical methods in natural language processing (EMNLP); 2020.

39.	Zschornack Rodrigues Saraiva F, Linhares Coelho da Silva T, Fernandes de Macêdo JA, editors. Aspect term extraction using deep learning model with minimal feature engineering. Advanced Information Systems Engineering: 32nd International Conference, CAiSE 2020, Grenoble, France, June 8–12, 2020, Proceedings 32; 2020: Springer.

40.	Chen Z, Qian T, editors. Enhancing aspect term extraction with soft prototypes. Proceedings of the 2020 Conference on Empirical Methods in Natural Language Processing (EMNLP); 2020.

41.	Wei Z, Hong Y, Zou B, Cheng M, Yao J, editors. Don’t eclipse your arts due to small discrepancies: Boundary repositioning with a pointer network for aspect extraction. Proceedings of the 58th annual meeting of the association for computational linguistics; 2020.

42.	Shi J, Li W, Bai Q, Ito T. BeeAE: effective aspect term extraction with artificial bee colony. The Journal of Supercomputing. 2022;78(16):17969-91.

43.	Pour AAM, Jalili S, editors. Aspects extraction for aspect level opinion analysis based on deep CNN. 2021 26th International Computer Conference, Computer Society of Iran (CSICC); 2021: IEEE.

44.	Wang R, Liu C, Zhao R, Yang Z, Zhang P, Wu D. Post-processing method with aspect term error correction for enhancing aspect term extraction. Applied Intelligence. 2022;52(14):15751-63.

45.	Ganapathibhotla M, Liu B, editors. Mining opinions in comparative sentences. Proceedings of the 22nd international conference on computational linguistics (Coling 2008); 2008.

46.	Li Z, Qin Y, Liu Z, Wang W. Powering comparative classification with sentiment analysis via domain adaptive knowledge transfer. arXiv preprint arXiv:210903819. 2021.

47.	Xu Q, Hong Y, Zhao F, Song K, Chen J, Kang Y, et al., editors. GCN-based End-to-End Model for Comparative Opinion Quintuple Extraction. 2023 International Joint Conference on Neural Networks (IJCNN); 2023: IEEE.

48.	OpenAI. Models [Internet]. 2024 [cited 2024 Sep 23]. Available from: https://platform.openai.com/docs/models/

49.	Touvron H, Martin L, Stone K, Albert P, Almahairi A, Babaei Y, et al. Llama 2: Open foundation and fine-tuned chat models. arXiv preprint arXiv:230709288. 2023.

50.	Bai J, Bai S, Chu Y, Cui Z, Dang K, Deng X, et al. Qwen technical report. arXiv preprint arXiv:230916609. 2023.

**Additional Editor Comments:**

There are some minor issues listed that authors need to resolve to consider this paper for publication.

### Response to Additional Editor Comments:

Dear Editor,

We appreciate the Editor’s note regarding minor issues. We have thoroughly addressed all the minor issues listed in your comments to ensure our manuscript meets PLOS ONE’s standards for publication. These revisions include formatting adjustments, typographical corrections, and clarification of specific sections as needed. Additionally, to honor the reviewers' input and ensure the thoroughness of our response, we have included both previous manuscript references and the newly added references in our response letter. All references have been meticulously converted to **Vancouver style** and checked for accuracy, resulting in the addition of References [4] and [6], the replacement of the former Reference [22] with Reference [25] to include a more current and relevant source, and the renumbering of existing references to maintain consistency and adherence to the journal’s formatting guidelines. In response to Reviewer #1, we have introduced the **RoBERTa_base_go_emotions** model earlier in the manuscript, corrected its citation to include the fine-tuned **GoEmotions** resource, and clarified our focus on prompt-based evaluations over fine-tuning generative models. These changes enhance the clarity and quality of our manuscript, aligning it with the journal’s requirements. We trust that these revisions fulfill the journal’s criteria, and we remain available to provide any additional clarifications or materials as necessary. Once again, we appreciate your guidance and consideration.

Sincerely,
Dr. Afsaneh Fatemi,
Corresponding Author on behalf of all co-authors

[Note: HTML markup is below. Please do not edit.]

Reviewers' comments:

Reviewer's Responses to Questions

### Opening Response to the Reviewers

**Dear Reviewers,**

Thank you for your thorough evaluation and constructive feedback on our manuscript. We greatly appreciate the time and expertise each of you has dedicated to helping us improve our work. Your comments have enabled us to clarify the methodology, strengthen our findings, and enhance the overall coherence of the paper. Below, we provide point-by-point responses to each of your comments, detailing the corresponding revisions we have made to address your valuable insights.

Warm regards,
Dr. Afsaneh Fatemi,

Corresponding Author on behalf of all co-authors

**Comments to the Author**

1. If the authors have adequately addressed your comments raised in a previous round of review and you feel that this manuscript is now acceptable for publication, you may indicate that here to bypass the “Comments to the Author” section, enter your conflict of interest statement in the “Confidential to Editor” section, and submit your "Accept" recommendation.

Reviewer #1: All comments have been addressed

Reviewer #2: All comments have been addressed

2. Is the manuscript technically sound, and do the data support the conclusions?

The manuscript must describe a technically sound piece of scientific research with data that supports the conclusions. Experiments must have been conducted rigorously, with appropriate controls, replication, and sample sizes. The conclusions must be drawn appropriately based on the data presented.

Reviewer #1: Yes

Reviewer #2: Yes

3. Has the statistical analysis been performed appropriately and rigorously?

Reviewer #1: Yes

Reviewer #2: Yes

4. Have the authors made all data underlying the findings in their manuscript fully available?

The [PLOS Data policy](http://www.plosone.org/static/policies.action#sharing) requires authors to make all data underlying the findings described in their manuscript fully available without restriction, with rare exception (please refer to the Data Availability Statement in the manuscript PDF file). The data should be provided as part of the manuscript or its supporting information, or deposited to a public repository. For example, in addition to summary statistics, the data points behind means, medians and variance measures should be available. If there are restrictions on publicly sharing data—e.g. participant privacy or use of data from a third party—those must be specified.

Reviewer #1: Yes

Reviewer #2: Yes

5. Is the manuscript presented in an intelligible fashion and written in standard English?

PLOS ONE does not copyedit accepted manuscripts, so the language in submitted articles must be clear, correct, and unambiguous. Any typographical or grammatical errors should be corrected at revision, so please note any specific errors here.

Reviewer #1: Yes

Reviewer #2: Yes

6. Review Comments to the Author

Please use the space provided to explain your answers to the questions above. You may also include additional comments for the author, including concerns about dual publication, research ethics, or publication ethics. (Please upload your review as an attachment if it exceeds 20,000 characters)

**Reviewer #1:** The authors have again substantially extended the article text (56 pages vs
32 pages in version 1) with detailed reactions to reviewers comments and
further work of the authors. The presented SCQRD dataset has been expanded
almost three times offering its enlarged version denoted as
Smartphone-SCQRE and a version adapted from the public CompSent-19 dataset
named here as the Brands-CompSent-19-SCQRE dataset. This has led to
improved coverage of previously underrepresented question types. Both
datasets have undergone a newly adapted annotation process with more
annotators and improved Inter-Annotator Agreement evaluation.

In the previous round, the authors have based the model architecture on the
RoBERTa base model. In the current text, the proposed system exploits
a specific model of RoBERTa_base_go_emotions, which is mentioned more than
40 times in the text (even used as one of the article keywords) but not
properly introduced until Section 3.3.1 at page 22. Moreover, the citation
with the model is not a correct one, as it refers to the original RoBERTa
model (without fine-tuning for emotions). If the model choice is so
crucial, for the task, it should be briefly introduced near its first
mention.

The RoBERTa_base_go_emotions model is evaluated together with newer
generative models (GPT-3.5-turbo-0613, Llama 2 70B Chat, and Qwen 1.5 7B
Chat) that were employed using their native prompt-based tasks. However,
for the purpose of Text Classification (like RoBERTa_base_go_emotions), the
generative models can be standardly fine-tuned with extra classification
layer(s). This usually offers better results than just prompting. But even
evaluating the prompt-based approaches (with the level of details as
offered in the article) is a valuable experiment.

The presented SCRQE model has been newly trained with the extended datasets
and with augmented reversed comparative statements which improved the
generalization capabilities and the resulting scores of the proposed model
so that it now outperforms a previously better model.


Overall, I believe the article is acceptable, with the correction of the
late introduction of the RoBERTa_base_go_emotions model.

### Response to Reviewer #1

We sincerely appreciate your thoughtful review of our revised manuscript and the positive assessment of our work. Thank you for highlighting the strengths of our expanded dataset and the improvements to the Inter-Annotator Agreement evaluation. Below, we address each of your comments in detail:

1. **Late Introduction of the RoBERTa_base_go_emotions Model**
   - **Issue:** We agree with your observation that the **RoBERTa_base_go_emotions** model was introduced too late in the manuscript, despite its frequent appearance in the text.
   - **Revision Action:**
     - In the Introduction, we have now included a concise, standalone subsection that explains:
       1. What **RoBERTa_base_go_emotions** is,
       2. Why it is particularly suitable for our task, and
       3. How it differs from the original RoBERTa.
     - The revised text reads as follows:

*“This paper introduces a multi-phase extraction pipeline leveraging a specialized variant of the RoBERTa architecture, called RoBERTa_base_go_emotions [4]. Unlike the original RoBERTa [5], this model has been fine-tuned on the GoEmotions dataset [6], which comprises 58k Reddit comments annotated for 27 distinct emotional categories plus a neutral category. By leveraging its ability to capture nuanced emotional undertones, our approach gains enhanced sensitivity to subjective expressions of preference, intensities, and user constraints—elements that are especially crucial for subjective comparative questions. Detailed information about the RoBERTa_base_go_emotions model and its application in our study is thoroughly discussed in Section 3.3.1.”*

- This ensures readers understand why **RoBERTa_base_go_emotions** plays a central role in our approach from the outset.

2. **Correct Citation**
   - **Issue:** The citation for **RoBERTa_base_go_emotions** previously referred only to the original RoBERTa paper, rather than acknowledging the fine-tuned GoEmotions resource.
   - **Revision Action:**
     - Although we initially provided the **Hugging Face link** to “https://huggingface.co/SamLowe/roberta-base-go_emotions” as a footnote, we now recognize that a **direct citation** of this specialized variant—and the GoEmotions dataset—is essential for clarity.
     - To correct this, we have updated our references to include both the **original RoBERTa** publication [Liu et al., 2019] and the **SamLowe/roberta-base-go_emotions** model [4.	Lowe S. roberta-base-go_emotions [Internet]. HuggingFace. 2021 [cited 2024 Sep 23]. Available from: https://huggingface.co/SamLowe/roberta-base-go_emotions], as well as the **GoEmotions** dataset [Demszky et al., 2020].
     - In Section 3.3.1, we now clarify the model’s lineage and cite each relevant source. The revised text includes:

*“The roberta-base-go_emotions [4] model was chosen for this task due to its robust ability to handle emotional nuances in subjective comparative questions. Unlike the original RoBERTa [5], which was not specifically fine-tuned for emotion-related tasks, roberta-base_go_emotions has been fine-tuned on the GoEmotions dataset [6], a collection of over 58,000 English-language Reddit comments annotated for 28 distinct emotional categories (27 emotions plus a neutral category). …”*

- This explicitly distinguishes the **roberta-base_go_emotions** model from the base RoBERTa, clarifying that it is a specialized variant for multi-label emotion classification and ensuring the citation now points to both the original architecture and the fine-tuned model.

**3. Evaluation of Generative Models vs. Fine-Tuned Classification Approaches**

- **Issue:** We acknowledge your point that large generative models (e.g., GPT-3.5-turbo-0613, Llama 2 70B Chat, Qwen 1.5 7B Chat) can be fine-tuned with classification layers, potentially yielding stronger performance than simple prompting.
- **Revision Action:**
  - We have revised the manuscript to **clarify our focus** on prompt-based evaluations, in Section 4.5.3, “Comparison with Prompt-Based Models”, where we explain:

“While these generative models can indeed be fine-tuned with dedicated classification layers, we chose to focus on prompt-based evaluations to provide a straightforward zero-/few-shot comparison across different model families. Fine-tuning GPT-3.5-turbo, for example, necessitates commercial API access, while Llama-2-70B and Qwen-1.5-7B require significant computational resources to fine-tune locally. Consequently, exploring the fine-tuning route for these models is a promising direction for future work, but lies beyond the scope of this study.”

- Additionally, in **Section 5** (“Conclusion and Future Work”), we note that:

“While we focused on prompt-based evaluations in this work, exploring fine-tuning approaches for large generative models (e.g., GPT-3.5, Llama 2, and Qwen) is a promising future direction that may yield further improvements for complex comparative tasks.”

- These additions clarify that our current study centers on prompt-based methods for practical comparability across model families, while also **acknowledging** that fine-tuning these models remains a **promising avenue** for future research.

We believe these revisions comprehensively address your comments and improve the manuscript’s clarity, particularly regarding the introduction of **RoBERTa_base_go_emotions**, the distinction between the base RoBERTa model and its fine-tuned variant, and our rationale for using prompt-based approaches. Thank you again for your insightful feedback and for recommending our article for publication. We greatly appreciate the time and effort you have invested in reviewing our work.

**To honor the reviewer's input and ensure the thoroughness of our response, we have also included these additions and revisions here in our response letter:**

**1. Introduction**

This paper introduces a multi-phase extraction pipeline leveraging a specialized variant of the RoBERTa architecture, called RoBERTa_base_go_emotions [4]. Unlike the original RoBERTa [5], this model has been fine-tuned on the GoEmotions dataset [6], which comprises 58k Reddit comments annotated for 27 distinct emotional categories plus a neutral category. By leveraging its ability to capture nuanced emotional undertones, our approach gains enhanced sensitivity to subjective expressions of preference, intensities, and user constraints—elements that are especially crucial for subjective comparative questions. Detailed information about the RoBERTa_base_go_emotions model and its application in our study is thoroughly discussed in Section 3.3.1.

**3.3.1. Justification for Using RoBERTa_base_go_emotions**

The roberta-base-go_emotions^[1]^ [4] model was chosen for this task due to its robust ability to handle emotional nuances in subjective comparative questions. Unlike the original RoBERTa [5], which was not specifically fine-tuned for emotion-related tasks, roberta-base-go_emotions has been fine-tuned on the GoEmotions dataset [6], a collection of over 58,000 English-language Reddit comments annotated for 28 distinct emotional categories (27 emotions plus a neutral category). This crucial distinction enables the model to detect and assign multiple emotional labels to a single text input, making it especially effective for tasks where subjective and emotionally nuanced comparisons are central. Developed by SamLowe, this specialized variant inherits the Transformer architecture of the roberta-base model while integrating multi-label classification capabilities to capture subtle emotional undertones.

The roberta-base-go_emotions model is built upon the RoBERTa-base architecture, which features a 12-layer Transformer, 12 self-attention heads, a 768-dimensional hidden layer, and around 125 million parameters. Unlike BERT, RoBERTa introduced dynamic masking, removed the next sentence prediction (NSP) task, and leveraged a pretraining corpus approximately ten times larger than that of BERT (i.e., 160 GB vs. 16 GB). As a result of this extensive pretraining, RoBERTa-base yields robust representations well suited for a variety of complex NLP tasks. The roberta-base-go_emotions variant then fine-tunes this architecture on the GoEmotions dataset—over 58,000 Reddit comments annotated for 28 emotional categories—enabling it to capture nuanced emotional undertones critical for tasks involving subjective and emotionally charged content.

In the realm of subjective comparative analysis, the interpretation of preferences and evaluations between entities is often influenced by personal opinions imbued with subtle emotional undertones. The GoEmotions dataset, which underpins the roberta-base-go_emotions model, provides a rich tapestry of emotional contexts, equipping the model with the necessary tools to adeptly navigate and interpret nuanced expressions. This capability is crucial for subjective comparative relation extraction tasks. By accurately identifying these emotional nuances, the model enhances the extraction of entities, aspects, and preferences—components where emotional contexts significantly impact user comparisons.

The fine-tuning of the roberta-base-go_emotions model on the GoEmotions dataset equips it to excel in scenarios where user preferences are inherently tied to emotional content. Its multi-label classification capability allows the model to recognize overlapping and co-occurring emotional states, providing a detailed representation of the input’s sentiment. This added layer of precision enables the model to remain highly sensitive to emotional subtleties that are often overlooked by traditional NLP models. As a result, roberta-base-go_emotions is particularly well-suited for tasks involving subjective comparative questions, where the ability to detect and incorporate emotional nuances significantly enhances the accuracy, relevance, and contextual appropriateness of the extracted relations.

**4.5.3. Comparison with Prompt-Based Models**

While these generative models can indeed be fine-tuned with dedicated classification layers, we chose to focus on prompt-based evaluations to provide a straightforward zero-/few-shot comparison across different model families. Fine-tuning GPT-3.5-turbo, for example, necessitates commercial API access, while Llama-2-70B and Qwen-1.5-7B require significant computational resources to fine-tune locally. Consequently, exploring the fine-tuning route for these models is a promising direction for future work, but lies beyond the scope of this study.

**5. Conclusion and Future Work**

While we focused on prompt-based evaluations in this work, exploring fine-tuning approaches for large generative models (e.g., GPT-3.5, Llama 2, and Qwen) is a promising future direction that may yield further improvements for complex comparative tasks.

**Reviewer #2:** (No Response)

7. PLOS authors have the option to publish the peer review history of their article ([what does this mean?](https://journals.plos.org/plosone/s/editorial-and-peer-review-process#loc-peer-review-history)). If published, this will include your full peer review and any attached files.


If you choose “no”, your identity will remain anonymous but your review may still be made public.


**Do you want your identity to be public for this peer review?** For information about this choice, including consent withdrawal, please see our [Privacy Policy](https://www.plos.org/privacy-policy).

Reviewer #1: No

Reviewer #2: No

[NOTE: If reviewer comments were submitted as an attachment file, they will be attached to this email and accessible via the submission site. Please log into your account, locate the manuscript record, and check for the action link "View Attachments". If this link does not appear, there are no attachment files.]

While revising your submission, please upload your figure files to the Preflight Analysis and Conversion Engine (PACE) digital diagnostic tool, <https://pacev2.apexcovantage.com/>. PACE helps ensure that figures meet PLOS requirements. To use PACE, you must first register as a user. Registration is free. Then, login and navigate to the UPLOAD tab, where you will find detailed instructions on how to use the tool. If you encounter any issues or have any questions when using PACE, please email PLOS at [figures@plos.org](mailto:figures@plos.org). Please note that Supporting Information files do not need this step.

We will upload our figure files to the Preflight Analysis and Conversion Engine (PACE) at <https://pacev2.apexcovantage.com/> and follow the platform’s guidelines to ensure our figures meet PLOS requirements. After registering for free and logging in, we will navigate to the “UPLOAD” tab for detailed instructions. If we encounter any issues or have questions about the process, we will contact PLOS at figures@plos.org.

*In compliance with data protection regulations, you may request that we remove your personal registration details at any time.* [*(Remove my information/details)*](https://www.editorialmanager.com/pone/login.asp?a=r)*. Please contact the publication office if you have any questions.*

### Conclusion to All Reviewers

**Dear Reviewers,**

We wish to extend our sincere gratitude for your constructive critiques and insightful recommendations. Your thorough evaluations have been instrumental in refining our manuscript, prompting us to improve the clarity, rigor, and overall quality of our research. We believe that the revisions made in response to your feedback have substantially strengthened the manuscript and enhanced its contribution to the field.

Thank you once again for your time, expertise, and dedication in reviewing our work.

Sincerely,
Dr. Afsaneh Fatemi,
Corresponding Author on behalf of all co-authors

### Overall Conclusion to the Editor

**Dear Dr. Budler,**

We appreciate the opportunity to revise our submission based on the reviewers’ comments. Through the detailed point-by-point responses provided, we have addressed each concern and revised the manuscript accordingly. We have also ensured that our references are up-to-date, with any newly added references properly noted, and we have confirmed that none of the sources cited have been retracted.

We remain available to answer any further questions or make additional modifications as needed. Thank you for your consideration, and we hope this revised manuscript meets the criteria for publication in your esteemed journal.

Sincerely,
Dr. Afsaneh Fatemi,
Corresponding Author on behalf of all co-authors

1. https://huggingface.co/SamLowe/roberta-base-go_emotions
